# Preconception health among migrant women in England: A cross-sectional analysis of maternity services data 2018–2019

**DOI:** 10.1016/j.jmh.2024.100250

**Published:** 2024-07-27

**Authors:** Majel McGranahan, Elizabeth Augarde, Danielle Schoenaker, Helen Duncan, Sue Mann, Debra Bick, Felicity Boardman, Oyinlola Oyebode

**Affiliations:** a University of Warwick Medical School, Medical School Building, Coventry CV4 7HL, UK; b Office for Health Improvement and Disparities (OHID), 39 Victoria Street, Westminster, London SW1H 0EU, UK; c School of Primary Care, Population Sciences and Medical Education, Faculty of Medicine, University of Southampton, Tremona Road SO16 6YD, Southampton, UK; d NIHR Southampton Biomedical Research Centre, University of Southampton and University Hospital Southampton NHS Foundation Trust, Tremona Road, SO16 6YD, Southampton, UK; e NHS England and Improvement, Wellington House, London SE1 8UG, UK; f Warwick Clinical Trials Unit, University of Warwick, Gibbet Hill Road, Coventry CV4 7AL, UK; g Wolfson Institute of Population Health, Barts and The London School of Medicine and Dentistry, Queen Mary University of London, EC1M 6BQ, UK

**Keywords:** Preconception health, Reproductive health, Migration, Asylum seeker, Refugee

## Abstract

**Background:**

Perinatal outcomes are poor among migrant women in vulnerable situations, but little is known about their health preconception. We investigated preconception health inequalities between migrant women in vulnerable situations and non-migrant women.

**Methods:**

This national cross-sectional study used data from the NHS Maternity Services Data Set (MSDS) version 1.5, incorporating NHS maternity services in England. All 652,880 women with an antenatal booking appointment between 1/4/2018 and 31/3/2019 were included. Migration category data were available for 66.2 % (*n* = 432,022). Odds ratios were calculated comparing preconception indicators among probable migrants in vulnerable situations (English not their first language with complex social factors (CSF)), probable migrants not in vulnerable situations (English not their first language without CSF), probable non-migrants in vulnerable situations (English their first language with CSF) and probable non-migrants not in vulnerable situations (English their first language without CSF). CSF include recent migrants, asylum seekers, refugees, difficulty reading/speaking English; alcohol and/or drugs misuse; aged under 20; and/or experiencing domestic abuse.

**Findings:**

We identified 3.8 % (25,070 women) of the study population as probable migrants in vulnerable situations, 10.2 % (66,783 women) as probable migrants not in vulnerable situations, 5.6 % (36,433 women) as probable non-migrants in vulnerable situations, 46.5 % (303,737 women) as probable non-migrants not in vulnerable situations, and 33.8 % as having missing migration category data. Probable migrants in vulnerable situations (*n* = 25,070) had over twice the odds of not taking folic acid preconception compared to probable non-migrants not in vulnerable situations (odds ratio 2.15, 95 % confidence interval 2.06–2.25). They had increased odds of previous obstetric complications and being underweight, but lower odds of physical and mental health conditions (apart from diabetes and hepatitis b), smoking and overweight or obesity.

**Interpretation:**

Inequalities exist across many preconception indicators, highlighting opportunities to improve preconception health in this population to reduce health inequalities and improve perinatal and neonatal outcomes.

**Funding:**

Medical Research Council.

## Highlights

• Preconception health inequalities exist for migrant women in vulnerable situations.
• Areas of inequality include folic acid use preconception, diabetes and underweight.
• Improvements in the preconception health of this population are needed.

## 1 Introduction

Nearly a third (28.8 %) of women giving birth in England and Wales in 2021 were migrants (born abroad themselves) (Office for National Statistics, 2022). Maternal mortality for women from certain countries is higher than for UK-born women; for example, between 2018 and 2020, women born in Bangladesh were over three times more likely to die during or after childbirth than UK-born women (Knight et al., 2022). Certain migrant groups, such as asylum seekers and refugees, have particularly complex social and health needs, resulting from poverty, trauma, and financial and structural barriers to healthcare (Heslehurst et al., 2018), and migrant women are sometimes victims of forced labour, trafficking and sexual assault (Abubakar et al., 2018).

Recent umbrella systematic reviews have highlighted the link between preconception risks and health behaviours with maternal and neonatal outcomes (Caut et al., 2022; Daly et al., 2022). For example, overweight or obesity preconception increases risks of miscarriage and adverse maternal and neonatal outcomes such as pre-eclampsia and stillbirth (Caut et al., 2022), whilst maternal folic acid supplementation preconception is associated with fewer anomaly-related terminations and neural tube defects (Daly et al., 2022).

Migrant women are at particular risk of poor preconception and maternal health. Structural barriers, institutional barriers, and fear of deportation and charging prevent some women from accessing antenatal care (Phillimore, 2016; Jones et al., 2022). Refugees from certain countries have higher rates of tuberculosis, hepatitis B and HIV (Crawshaw et al., 2018), and may not be fully vaccinated (Mipatrini et al., 2017). A Doctors of the World (DOTW) report showed approximately one in twenty (6 %) migrant women accessing the London DOTW clinic were taking folic acid preconception (Jones et al., 2022), compared to 25.9 % in the general population nationally (NHS Maternity Statistics, 2023). Nearly half (45 %) had their first antenatal appointment after 16 weeks (Jones et al., 2022), compared to 10 % in the general population (NHS Maternity Statistics, 2023). National Institute for Health and Care Excellence (NICE) guidelines recommend that the first antenatal appointment occurs by week 10 of pregnancy (NICE, 2021).

Despite potential for higher preconception risks among migrant women, and known links between preconception risks and pregnancy outcomes, no previous studies have compared migrant and non-migrant women's preconception health in England. It is therefore not known where the biggest preconception health inequalities lie, and where policy or health interventions could be targeted to improve preconception health in this population, and ultimately reduce maternal health inequalities.

Our objective was to examine inequalities in preconception health between migrant women in vulnerable situations and non-migrant women. Our definition of migrant women in vulnerable situations includes asylum seekers and refugees, recent migrants and those with difficulty speaking or reading English.

*The terms women/woman are used throughout this manuscript, but it is recognised that not all pregnant people identify as women.

## 2 Materials and methods

### 2.1 Setting

This national cross-sectional study used data from the National Health Service (NHS) Maternity Services Data Set (MSDS) version 1.5. MSDS re-uses operational and clinical data for examining health inequalities, monitoring health outcomes, commissioning and planning services (NHS Digital 2019). All NHS-funded maternity units in England are required to submit their data to MSDS, with data available at patient-level, from the first antenatal appointment to discharge from maternity services (NHS Digital 2019). Some MSDS data items are mandatory and must be reported, some are required, others are optional. Only data collected at the first antenatal appointment (the ‘booking appointment’) were accessed. Women with a booking appointment between 1/4/2018 and 31/3/2019 were included.

As per the Health and Social Care Act 2012, participant consent was not required. Data were accessed through the Office for Health Improvement and Disparities (OHID). Data were anonymised and provided to OHID by NHS Digital, for the purposes of population health surveillance, with a relevant data sharing agreement.

### 2.2 Participants

Inclusion Criteria: all women with a booking appointment between 1/4/2018 and 31/3/2019 were included.

Exclusion Criteria: there were no exclusion criteria. Women contributed data for all the analyses that they had complete data for.

A binary variable—‘complex social factors’ (CSF)—was used to help identify probable migrants. This variable, based on NICE Guidance CG110, is collected at the booking appointment, and includes women who are recent migrants, asylum seekers or refugees, have difficulty reading or speaking English; women who misuse alcohol and/or drugs; women aged under 20; and/or women who experience domestic abuse (National Institute of Health and Clinical Excellence, 2010). It was not possible to identify why a woman was assigned CSF from available data. CSF was used alongside the variable for English as a first language to allow preconception and maternal health indicators to be compared between:

1. Probable migrants in vulnerable situations (do not speak English as their first language with CSF)
2. Probable migrants not in vulnerable situations (do not speak English as their first language without CSF)
3. Probable non-migrants in vulnerable situations (speak English as their first language with CSF)
4. Probable non-migrants not in vulnerable situations (speak English as their first language without CSF).

By combining information on English not being a first language with CSF, the exposure group is likely to be a group of migrant women in potentially vulnerable situations: either asylum seekers or refugees (Vulnerable Migrants: Migrant Health Guide - GOV.UK, 2022), recent migrants, or those who have difficulty reading or speaking English (National Institute of Health and Clinical Excellence, 2010). Some women in this group may not be migrants; however, most (99 %) UK-born people speak English as a first language (English Language use and Proficiency of Migrants in the UK - Migration Observatory - The Migration Observatory, 2022). There are few countries outside the UK where the majority speak English as a native language, as even in countries where English is an official language most people will have another language as their mother tongue (Immigration Rules - Immigration Rules Appendix English Language - Guidance - GOV.UK, 2023). The second group is likely to include some migrants, but may not include those in the most vulnerable situations as they do not have CSF. The third group may include some migrants (for example, CSF due to being an asylum seeker) but since this group speak English as a first language, people may be more likely to have CSF for reasons other than migration. The fourth group is unlikely to include many migrants as they speak English as a first language and do not have CSF.

### 2.3 Variables examined

Preconception indicators identified from a previous review and report card of priority preconception indicators for national surveillance using MSDS were included (Schoenaker et al., 2021; Schoenaker et al., 2023) (Table 2). Preconception indicators referred to the current pregnancy. All preconception indicators were converted to binary variables for analysis. We also included data regarding late antenatal booking (after 10 weeks, after 16 weeks and after 20 weeks as three binary variables), because this has been identified as an issue for migrant women in previous studies (Jones et al., 2022; Higginbottom et al., 2019) and may reflect service engagement preconception. Alcohol consumption and substance use were considered relevant to migrant category allocation (because they would lead to a woman having CSF recorded) so were not included.

Mother's age at booking (years), ethnicity (as a categorical variable: white, mixed, Asian, black, other or ‘missing’), number of previous live births and Index of Multiple Deprivation 2015 (IMD decile) were obtained to address possible confounding.

Data on women's preconception health and behaviour available from the booking antenatal appointment within MSDS are obtained retrospectively, including self-reported folic acid use and substance use, and medical history. During the first face-to-face appointment, height and weight are measured, and Body Mass Index (BMI) calculated (NICE, 2021). IMD relates to the lower super output area (LSOA) of residence, determined from a woman's postcode.

Some variables within MSDS record previous pregnancy and delivery events. For the previous caesarean sections variable, values were recoded as ‘missing’ if more than 10 caesarean sections were reported for a single individual (based on discussion with experts who considered values above this number must be spurious). In total, 3791 records had more than 10 caesarean sections reported: these values were recorded as ‘missing’. For the previous live births variable, values were recoded as ‘missing’ if more than 25 live births were reported (based on an upper bound of 38 years of fertility with one baby born per 18 months). In total, there were 494 records with the number of live births recorded as above 25. BMI was recoded as ‘missing’ if it was reported as above 80 or below 13, or if the booking appointment was after 14 weeks because pregnancy affects BMI (in line with OHID methodology for national Child and Maternal Health statistics Child and Maternal Health - Data - OHID, 2022). Overall, 2079 records had a recorded BMI above 80 or below 13, and 57,445 records had a booking appointment after 14 weeks. For preconception indicators related to previous obstetric conditions, only women with at least one previous pregnancy were included in analyses.

### 2.4 Statistical analysis

Descriptive statistics were used to describe women's age at booking, number of previous live births, ethnicity, and deprivation decile (IMD) across the study population, and by migration category. Prevalence of each preconception indicator was calculated, with 95 % confidence intervals for the study population, and by migration category.

Odds ratios and 95 % confidence intervals were calculated using binary logistic regression for each preconception indicator according to migration category, with probable non-migrants not in vulnerable situations as the reference category. The outcome for each model consisted of individuals with the preconception indicator (e.g. those with diabetes) compared to individuals without the preconception indicator (e.g. those without diabetes).

We included two binary logistic regression models for BMI because we were interested in knowing if there were increased/decreased odds of being just underweight in the probable migrant group, and if there were increased/decreased odds of being overweight or obese in this group (and because a multinomial or ordered logit model would not be possible in this context). In the first BMI model, we combined the overweight and obese categories to examine the odds of being ‘overweight or obese’ versus ‘underweight or normal’. For the second BMI model, we combined overweight, obese and normal to examine the odds of being ‘underweight’ versus overweight, obese or normal.

Age (years), ethnicity (white, mixed, Asian, black, other or ‘missing’), and most deprived IMD decile (as a binary variable) were included as covariates in multivariable models because they were considered to be potential confounding factors. Ethnicity was included because ethnicity is linked to maternal outcomes (Knight et al., 2022) and may have a relationship with migration (Zhang et al., 2022); mother's age and deprivation were included as covariates because they are linked to maternal outcomes (Knight et al., 2022) and may be associated with migration. Number of previous live births was only included as a covariate in models for which it was considered a potential confounder, namely: previous obstetric complications, health behaviours (women may have received more pregnancy-related health behaviour advice through previous pregnancies), underweight, overweight or obesity (weight gain frequently occurs between pregnancies Ziauddeen et al., 2019) and late antenatal booking (women with previous live births may be more aware of the antenatal care system, or may have childcare commitments precluding early booking). Preconception indicators which were not considered to be confounded by number of previous live births (and so number of live births was not included as a covariate) were: pre-existing mental or physical health conditions, inherited conditions, family history of diabetes, ‘Unemployed and seeking work’, and ‘No adequate support available during and after pregnancy’.

Five models were calculated for each preconception indicator, using forced entry model fitting. Model 1 included migration category as the exposure variable and the preconception indicator as the outcome variable. Participants with missing ‘CSF’ and/or ‘English as a first language’ were categorised as ‘missing’ migration category and included in the model. Model 2 adjusted for most deprived IMD decile, mother's age and ethnicity, and number of previous live births (in models for which number of live births was considered a potential confounder as described above).

To examine the contribution of adjustment for ethnicity, Model 3 replicated Model 2 but without adjusting for ethnicity. To examine the effect of excluding individuals with missing data, Model 4 excluded participants with missing data for migration category and/or ethnicity (because these variables had missing data of 33.8 % and 15.8 % respectively). To assess potential bias due to misclassification of migration category due to age or substance use resulting in CSF being recorded, Model 5 replicated Model 2, but excluded under 20 s and those with recorded history of substance use.

Data management and analysis were undertaken using R version 4.1.3.

### 2.5 Ethical approval

Ethical approval was obtained from the Biomedical and Scientific Research Ethics Committee of the University of Warwick Research Governance and Ethics Committee (Reference BSREC 158/21-22).

### 2.6 Role of the funding source

The funder had no role in the study design or implementation of the study, the writing of the report or the decision to submit for publication.

## 3 Results

### 3.1 Participants

Migration category data were available for 66.2 % of the study population (432,022 women), with 3.8 % (25,070 women) identified as probable migrants in vulnerable situations, 10.2 % (66,783 women) as probable migrants not in vulnerable situations, 5.6 % (36,433 women) as probable non-migrants in vulnerable situations, 46.5 % (303,737 women) as probable non-migrants not in vulnerable situations, and 33.8 % as having missing migration data (Table 1).

**Table 1:** Participant characteristics. *Complex social factors include women who misuse alcohol and/or drugs; women who are recent migrants, asylum seekers or refugees, or have difficulty reading or speaking English; all women aged under 20; and/or women who experience domestic abuse. SD: standard deviation. IQR: interquartile range.*

Characteristic	Overall *N* = 652,880	Probable migrants in vulnerable situations *N* = 25,070	Probable migrants not in vulnerable situations *N* = 66,783	Probable non-migrants in vulnerable situations *N* = 36,433	Probable non-migrants not in vulnerable situations *N* = 303,737	Missing migration data (first language and/or complex social factor data missing) *N* = 220,858
Mother's age at booking (years)						
Mean (SD)	29.8 (5.7)	28.1 (6.4)	30.9 (5.2)	24.4 (6.9)	30.4 (5.3)	29.8 (5.6)
Median (IQR)	30 (26 to 34)	28 (23 to 32)	31 (27 to 34)	22 (19 to 30)	30 (27 to 34)	30 (26 to 34)
Missing, *n*	9	0	0	0	0	9
Number of previous live births						
Mean (SD)	0.9 (1.1)	1.0 (1.3)	0.9 (1.1)	0.8 (1.3)	0.9 (1.1)	0.9 (1.2)
Median (IQR)	1 (0 to 1)	1 (0 to 1)	1 (0 to 1)	0 (0 to 1)	1 (0 to 1)	1 (0 to 1)
Missing, *n*	128,308	1733	4293	4757	30,311	87,214
Ethnicity of mother, *n* (%)						
White	424,453 (77.2)	8944 (45.0)	28,261 (52.0)	27,656 (85.2)	219,683 (82.4)	139,909 (79.4)
Mixed	11,695 (2.1)	438 (2.2)	1094 (2.0)	848 (2.6)	5547 (2.1)	3768 (2.1)
Asian	66,444 (12.1)	5638 (28.4)	16,299 (30.0)	1685 (5.2)	23,932 (9.0)	18,890 (10.7)
Black	26,172 (4.8)	1876 (9.4)	3691 (6.8)	1514 (4.7)	11,100 (4.2)	7991 (4.5)
Other	20,788 (3.8)	2987 (15.0)	4994 (9.2)	776 (2.4)	6404 (2.4)	5627 (3.2)
Missing, *n*	103,328	5187	12,443	3954	37,071	44,673
Index of multiple deprivation 2015 (IMD), *n* (%)						
IMD 1	92,528 (14.2)	7130 (28.4)	11,408 (17.1)	8280 (22.7)	31,389 (10.3)	34,321 (15.5)
IMD 2	82,397 (12.6)	5011 (20.0)	9876 (14.8)	5834 (16.0)	33,844 (11.1)	27,832 (12.6)
IMD 3	77,043 (11.8)	3900 (15.6)	9402 (14.1)	4975 (13.7)	33,866 (11.1)	24,900 (11.3)
IMD 4	70,7697 (10.8)	2705 (10.8)	8046 (12.0)	3995 (11.0)	33,484 (11.0)	20,689 (10.2)
IMD 5	63,917 (9.8)	2026 (8.1)	6672 (10.0)	3255 (8.9)	31,275 (10.3)	20,689 (9.4)
IMD 6	60,930 (9.3)	1561 (6.2)	6038 (9.0)	2686 (7.4)	31,238 (10.3)	19,407 (8.8)
IMD 7	56.858 (8.7)	1089 (4.3)	4854 (7.3)	2442 (6.7)	29,558 (9.7)	18,917 (8.6)
IMD 8	54,125 (8.3)	786 (3.1)	4262 (6.4)	2035 (5.6)	28,391 (9.3)	18,651 (8.4)
IMD 9	50,586 (7.7)	564 (2.2)	3588 (5.4)	1788 (4.9)	27,267 (9.0)	17,379 (7.9)
IMD 10	43,799 (6.7)	298 (1.2)	2638 (4.0)	1143 (3.1)	23,425 (7.7)	16,295 (7.4)
Missing, *n*	0	0	0	0	0	0

The mean age overall was 29.8 (standard deviation (SD) 5.7). Most (77.8 %) participants were of white ethnicity, whereas among probable migrants in vulnerable situations, 45.0 % were of white ethnicity. Probable migrants in vulnerable situations had the highest proportion in the most deprived decile (28.4 %).

### 3.2 Preconception indicators

Table 2 shows preconception indicators according to migration category. There were proportionally more probable migrants in vulnerable situations with known previous obstetric complications (34.4 %, 95 % Confidence Interval (CI) 33.6–35.2) compared to probable non-migrants not in vulnerable situations (26.9 %, 95 % CI 26.7–27.1). Proportionally more probable migrants in vulnerable situations did not take folic acid supplementation preconception (87.6 %, 95 % CI 87.2–88.1) than other groups. A higher proportion of probable migrants in vulnerable situations were underweight (BMI < 18.5), fewer were obese or had diagnosed mental or physical health conditions, although more had diabetes and hepatitis b. Late antenatal booking occurred more frequently among probable migrants in vulnerable situations.

**Table 2:** Preconception indicators by migration category. *Complex social factors include women who misuse alcohol and/or drugs; women who are recent migrants, asylum seekers or refugees, or have difficulty reading or speaking English; all women aged under 20; and/or women who experience domestic abuse. Missing migration category: first language and/or complex social factor data missing.*

Preconception indicator	Overall *N* = 652,880	Probable migrants in vulnerable situations *N* = 25,070	Probable migrants not in vulnerable situations *N* = 66,783	Probable non-migrants in vulnerable situations *N* = 36,433	Probable non-migrants not in vulnerable situations *N* = 303,737	Missing migration category *N* = 220,858
	*n* % (95 % CI)	*n* % (95 % CI)	*n* % (95 % CI)	*n* % (95 % CI)	*n* % (95 % CI)	*n* % (95 % CI)
Unemployed and seeking work (*n* = 472,181)	26,849	2295	2770	5601	11,897	4286
	5.7 (5.6–5.8)	10.6 (10.2–11.0)	4.5 (4.4–4.7 %)	19.0 (18.6–19.5)	4.4 (4.3–4.5)	4.8 (4.6–4.9)
Living in most deprived area (bottom 10 %) (*n* = 652,880)	92,528	7130	11,408	8280	31,389	34,321
	14.2 (14.1–14.3)	28.4 (27.9–29.0)	17.1 (16.8–17.4)	22.7 (22.3–23.2)	10.3 (10.2–10.4)	15.5 (15.4–15.7)
No adequate support available during and after pregnancy (*n* = 449,884)	26,590	686	3027	1463	9191	12,223
	5.9 (5.8–6.0)	3.2 (3.0–3.5)	5.2 (5.0–5.4)	4.9 (4.6–5.1)	3.6 (3.5–3.7)	14.3 (14.1–14.5)
Advanced maternal age (greater than or equal to 35 years) (*n* = 652,871)	139,661	4181	16,501	3713	69,901	45,365
	21.4 (21.3–21.5)	16.7 (16.2–17.1)	24.7 (24.4–25.0)	10.2 (9.9–10.5)	23.0 (22.9–23.2)	20.5 (20.4–20.7)
Teenage pregnancy (less than 20 years) (*n* = 652,871)	24,675	2820	0	15,612	0	6243
	3.8 (3.7–3.8)	11.2 (10.9–11.6)	0.0 (0.0–0.0)	42.9 (42.3–43.4)	0.0 (0.0–0.0)	2.8 (2.8–2.9)
Known previous obstetric complication (*n* = 329,228)	80,694	4722	13,136	4136	46,885	11,815
	24.5 (24.4–24.7)	34.4 (33.6–35.2)	32.9 (32.5–33.4)	24.3 (23.7–25.0)	26.9 (26.7–27.1)	14.0 (13.7–14.2)
Previous pre-eclampsia, eclampsia, gestational proteinuria, HELLP (*n* = 329,228)	3469	127	458	141	2054	689
	1.1 (1.0–1.1)	0.9 (0.8–1.1)	1.1 (1.0–1.3)	0.8 (0.7–1.0)	1.2 (1.1–1.2)	0.8 (0.8–0.9)
Previous gestational hypertension (*n* = 329,228)	5208	255	662	339	3170	782
	1.6 (1.5–1.6)	1.9 (1.6–2.1)	1.7 (1.5–1.8)	2.0 (1.8–2.2)	1.8 (1.8–1.9)	0.9 (0.9–1.0)
Previous gestational diabetes mellitus (*n* = 329,228)	7506	500	1735	243	3918	1110
	2.3 (2.2–2.3)	3.6 (3.3–4.0)	4.3 (4.2–4.6)	1.4 (1.3–1.6)	2.3 (2.2–2.3)	1.3 (1.2–1.4)
Previous caesarean section (*n* = 306,429)	69,989	3542	10,132	3226	37,446	15,643
	22.8 (22.7–23.0)	26.3 (25.5–27.0)	25.9 (25.5–26.3)	19.9 (19.3–20.5)	22.5 (22.3–22.7)	22.1 (21.8–22.4)
Previous pregnancy loss (*n* = 301,168)	117,258	4389	11,941	7459	60,274	33,195
	38.9 (38.8–39.1)	34.8 (33.9–35.6)	31.6 (31.2–32.1)	48.8 (48.0–49.6)	36.9 (36.7–37.1)	46.0 (45.6–46.3)
Previous live birth (*n* = 524,572)	288,073	12,293	35,677	14,045	152,511	73,547
	54.9 (54.8–55.1)	52.7 (52.0–52.3)	57.1 (56.7–57.5)	44.3 (43.8–44.9)	55.8 (55.6–56.0)	55.0 (54.8–55.3)
Not taken folic acid supplementation before pregnancy (488,987)	355,648	19,673	46,049	27,737	186,997	75,192
	72.7 (72.6–72.9)	87.6 (87.2–88.1)	75.1 (74.8–75.4)	84.8 (84.4–85.1)	70.0 (69.8–70.2)	71.3 (71.0–71.6)
Smoking around conception (*n* = 604,514)	117,602	4068	9051	14,538	53,559	36,386
	19.5 (19.5–19.7)	16.5 (16.0–16.9)	13.9 (13.7–14.2)	40.9 (40.4–41.4)	18.1 (18.0–18.3)	19.8 (19.6–20.0)
Smokers who did not quit smoking during year before pregnancy (*n* = 138,422)	117,602	4068	9051	14,538	53,559	36,386
	85.0 (84.9–85.2)	88.3 (87.3–89.2)	82.2 (81.5–82.9)	89.7 (89.2–90.1)	82.2 (81.9–82.5)	87.8 (87.5–88.1)
Underweight at booking (BMI less than 18.5 kg per m^2^) (*n* = 496,267)	15,345	871	1929	1846	6792	3907
	3.1 (3.0–3.1)	5.2 (4.9–5.6)	3.5 (3.3–3.6)	6.4 (6.2–6.7)	2.6 (2.5–2.7)	2.9 (2.8–3.0)
Overweight at booking (BMI 25 to 29.9 kg per m^2^) (*n* = 496,267)	138,155	4622	15,602	7204	72,797	37,930
	27.8 (27.7–28.0)	27.8 (27.1–28.5)	28.2 (27.9–28.6)	25.1 (24.6–25.7)	27.9 (27.7–28.1)	28.1 (27.9–28.3)
Obesity at booking (BMI greater than or equal to 30 kg per m^2^) (*n* = 496,267)	110,596	2866	9537	6654	59,884	31,655
	22.3 (22.2–22.4)	17.2 (16.7–17.8)	17.3 (16.9–17.6)	23.2 (22.7–23.7)	23.0 (22.8–23.1)	23.5 (23.2–23.7)
At least one mental or physical health condition (*n* = 652,880)	158,839	4424	17,361	11,761	99,456	25,837
	24.3 (24.2–24.4)	17.6 (17.2–18.1)	26.0 (25.7–26.3)	32.3 (31.8–32.8)	32.7 (32.6–32.9)	11.7 (11.6–11.8)
Mental health condition (*n* = 652,880)	60,973	1079	3890	7244	38,831	9929
	9.3 (9.3–9.4)	4.3 (4.1–4.6)	5.8 (5.6–6.0)	19.9 (19.5–20.3)	12.8 (12.7–12.9)	4.5 (4.4–4.6)
Physical health condition (*n* = 652,880)	124,705	3743	15,289	7064	78,817	19,792
	19.1 (19.0–19.2)	14.9 (14.5–15.4)	22.9 (22.6–23.2)	19.4 (19.0–19.8)	25.9 (25.8–26.1)	9.0 (8.8–9.1)
Diabetes (*n* = 652,880)	6343	411	1073	383	3434	1042
	1.0 (0.9–1.0)	1.6 (1.5–1.8)	1.6 (1.5–1.7)	1.1 (1.0–1.2)	1.1 (1.1–1.2)	0.5 (0.4–0.5)
Hypertension (*n* = 652,880)	6696	216	850	326	4358	946
	1.0 (1.0–1.1)	0.9 (0.8–1.0)	1.3 (1.2–1.4)	0.9 (0.8–1.0)	1.4 (1.4–1.5)	0.4 (0.4–0.5)
Cardiac disease (*n* = 652,880)	5184	132	579	323	3201	949
	0.8 (0.8–0.8)	0.5 (0.4–0.6)	0.9 (0.8–0.9)	0.9 (0.8–1.0)	1.1 (1.0–1.1)	0.4 (0.4–0.5)
Thromboembolic condition (*n* = 652,880)	3885	120	519	205	2473	568
	0.6 (0.6–0.6)	0.5 (0.4–0.6)	0.8 (0.7–0.8)	0.6 (0.5–0.6)	0.8 (0.8–0.8)	0.3 (0.2–0.3)
Renal disease (*n* = 652,880)	5126	183	625	291	2791	1236
	0.8 (0.8–0.8)	0.7 (0.6–0.8)	0.9 (0.9–1.0)	0.8 (0.7–0.9)	0.9 (0.9–1.0)	0.6 (0.5–0.6)
Hepatitis B (*n* = 652,880)	998	177	376	35	292	118
	0.2 (0.1–0.2)	0.7 (0.6–0.8)	0.6 (0.5–0.6)	0.1 (0.1–0.1)	0.1 (0.1–0.1)	0.1 (0.1–0.1)
Cancer (*n* = 652,880)	1085	20	102	52	742	169
	0.2 (0.2–0.2)	0.1 (0.1–0.1)	0.2 (0.1–0.2)	0.1 (0.1–0.2)	0.2 (0.2–0.3)	0.1 (0.1–0.1)
Inherited conditions (*n* = 652,880)	13,323	228	1192	1040	7592	3271
	2.0 (2.0–2.1)	0.9 (0.8–1.0)	1.8 (1.7–1.9)	2.9 (2.7–3.0)	2.5 (2.4–2.6)	1.5 (1.4–1.5)
Family history of diabetes (*n* = 652,880)	134,398	7124	20,513	9213	78,802	18,746
	20.6 (20.5–20.7)	28.4 (27.9–29.0)	30.7 (30.4–31.1)	25.3 (24.8–25.7)	25.9 (25.8–26.1)	8.5 (8.4–8.6)
Booking after 10 weeks gestation (*n* = 652,309)	275,191	15,665	30,676	17,282	117,063	94,505
	42.2 (42.1–42.3)	62.5 (61.9–63.1)	46.0 (45.6–46.3)	47.5 (47.0–48.0)	38.6 (38.4–38.7)	42.9 (42.6–43.1)
Booking after 16 weeks gestation (*n* = 652,309)	52,516	5483	5506	4332	15,970	21,225
	8.1 (8.0–8.1)	21.9 (21.4–22.4)	8.2 (8.0–8.5)	11.9 (11.6–12.2)	5.3 (5.2–5.3)	9.6 (9.5–9.7)
Booking after 20 weeks gestation (*n* = 652,309)	36,459	3630	3320	2893	10,590	16,026
	5.6 (5.5–5.6)	14.5 (14.1–14.9)	5.0 (4.8–5.1)	7.9 (7.7–8.2)	3.5 (3.4–3.6)	7.3 (7.2–7.4)

### 3.3 Logistic regression models

Table 3 shows the unadjusted and fully adjusted logistic regression models (Models 1 and 2 respectively). Models including sensitivity analyses and odds ratios for all covariates are included in Supplementary File 1.

**Table 3:** Logistic regression models for preconception indicators. Complex social factors include women who misuse alcohol and/or drugs; women who are recent migrants, asylum seekers or refugees, or have difficulty reading or speaking English; all women aged under 20; and/or women who experience domestic abuse. Missing migration category: first language and/or complex social factor data missing. Model 1 is unadjusted. Model 2 adjusts for mother's age at booking (years), most deprived Index of Multiple Deprivation (IMD) decile and ethnicity; Models marked with ‘^’ additionally adjust for number of previous live births. *p<0.001.

Indicator	Model	Probable non-migrant not in vulnerable situations	Probable migrants in vulnerable situations Odds ratio (95 % confidence interval)	Probable migrants not in vulnerable situations Odds ratio (95 % confidence interval)	Probable non-migrants in vulnerable situations Odds ratio (95 % confidence interval)	Missing migration category Odds ratio (95 % confidence interval)
Unemployed and seeking work	Model 1 (*n* = 472,181)	Reference	2.58 (2.46–2.70)*	1.03 (0.98–1.07)	5.10 (4.92–5.28)*	1.09 (1.05–1.13)*
	Model 2 (*n* = 472,175)	Reference	1.74 (1.66–1.83)*	0.96 (0.92–1.01)	2.84 (2.73–2.95)*	1.03 (0.99–1.07)
No adequate support available during and after pregnancy	Model 1 (*n* = 449,884)	Reference	0.89 (0.82–0.96)	1.47 (1.41–1.53)*	1.36 (1.29–1.44)*	4.46 (4.33–4.59)*
	Model 2 (*n* = 449,877)	Reference	1.10 (1.02–1.19)	1.75 (1.67–1.82)*	1.37 (1.29–1.45)*	4.56 (4.43–4.69)*
Known previous obstetric complication	Model 1 (*n* = 329,228)	Reference	1.42 (1.37–1.47)*	1.33 (1.30–1.36)*	0.87 (0.84–0.90)*	0.44 (0.43–0.45)*
	Model 2^ (*n* = 325,619)	Reference	1.26 (1.21–1.31)*	1.18 (1.15–1.21)*	0.91 (0.88–0.95)*	0.43 (0.42–0.44)*
Previous pre-eclampsia, eclampsia, gestational proteinuria, HELLP	Model 1 (*n* = 329,228)	Reference	0.78 (0.65–0.93)	0.97 (0.88–1.08)	0.70 (0.59–0.83)*	0.69 (0.63–0.75)*
	Model 2^ (*n* = 325,619)	Reference	0.69 (0.57–0.83)*	0.94 (0.84–1.04)	0.64 (0.54–0.76)*	0.67 (0.62–0.73)*
Previous gestational hypertension	Model 1 (*n* = 329,228)	Reference	1.02 (0.89–1.16)	0.91 (0.83–0.99)	1.10 (0.98–1.22)	0.50 (0.47–0.54)*
	Model 2^ (*n* = 325,619)	Reference	0.97 (0.85–1.11)	0.89 (0.81–0.97)	1.07 (0.95–1.20)	0.50 (0.47–0.55)*
Previous gestational diabetes mellitus	Model 1 (*n* = 329,228)	Reference	1.64 (1.49–1.80)*	1.97 (1.86–2.09)*	0.63 (0.55–0.71)*	0.58 (0.54–0.62)*
	Model 2^ (*n* = 325,619)	Reference	1.21 (1.01–1.24)	1.36 (1.28–1.45)*	0.68 (0.59–0.77)*	0.55 (0.51–0.59)*
Previous caesarean section	Model 1 (*n* = 306,429)	Reference	1.23 (1.18–1.28)*	1.21 (1.18–1.24)*	0.86 (0.82–0.89)*	0.98 (0.96–1.00)
	Model 2^ (*n* = 305,932)	Reference	1.13 (1.08–1.18)*	1.07 (1.04–1.10)*	1.02 (0.98–1.07)	0.97 (0.95–0.99)
Previous loss	Model 1 (*n* = 301,168)	Reference	0.91 (0.88–0.95)*	0.79 (0.77–0.21)*	1.63 (1.58–1.69)*	1.45 (1.43–1.48)*
	Model 2^ (*n* = 297,559)	Reference	1.06 (1.02–1.11)	0.85 (0.83–0.87)*	1.78 (1.72–1.86)*	1.45 (1.42–1.47)*
Not taken folic acid supplementation before pregnancy	Model 1 (*n* = 488,987)	Reference	3.04 (2.92–3.16)*	1.29 (1.27–1.32)*	2.38 (2.31–2.46)*	1.06 (1.05–1.08)*
	Model 2^ (*n* = 431,990)	Reference	2.15 (2.06–2.25)*	1.22 (1.19–1.25)*	1.49 (1.44–1.55)*	1.09 (1.07–1.11)*
Smoking around conception	Model 1 (*n* = 604,514)	Reference	0.89 (0.86–0.92)*	0.73 (0.71–0.75)*	3.12 (3.05–3.20)*	1.11 (1.10–1.13)*
	Model 2^ (*n* = 526,809)	Reference	0.86 (0.82–0.90)*	1.03 (1.00–1.06)	1.80 (1.74–1.87)*	1.05 (1.03–1.07)*
Smokers who did not quit smoking during year before pregnancy	Model 1 (*n* = 138,422)	Reference	1.63 (1.49–1.79)*	1.00 (0.95–1.05)	1.88 (1.78–1.99)*	1.56 (1.50–1.61)*
	Model 2^ (*n* = 121,676)	Reference	1.56 (1.41–1.73)*	1.21 (1.15–1.28)*	1.33 (1.23–1.44)*	1.32 (1.27–1.38)*
Underweight at booking (BMI less than 18.5 kg per m^2^)	Model 1 (*n* = 496,267)	Reference	2.07 (1.92–2.22)*	1.35 (1.28–1.42)*	2.58 (2.44–2.71)*	1.11 (1.07–1.16)*
	Model 2^ (*n* = 440,681)	Reference	1.55 (1.44–1.68)*	1.26 (1.19–1.33)*	1.60 (1.51–1.70)*	1.07 (1.03–1.12)
Overweight or obese at booking (BMI greater or equal to 25 kg per m^2^)	Model 1 (*n* = 496,267)	Reference	0.79 (0.77–0.82)*	0.81 (0.79–0.82)*	0.90 (0.88–0.93)*	1.03 (1.01–1.04)*
	Model 2^ (*n* = 440,681)	Reference	0.73 (0.71–0.75)*	0.77 (0.76–0.79)*	0.89 (0.87–0.92)*	1.01 (1.00–1.03)
At least one mental or physical health condition	Model 1 (*n* = 652,880)	Reference	0.44 (0.43–0.45)*	0.72 (0.71–0.74)*	0.98 (0.96–1.00)	0.27 (0.27–0.28)*
	Model 2 (*n* = 652,871)	Reference	0.54 (0.52–0.56)*	0.83 (0.82–0.85)*	1.06 (1.03–1.08)*	0.28 (0.28–0.28)*
At least one mental health condition	Model 1 (*n* = 652,880)	Reference	0.31 (0.29–0.33)*	0.42 (0.41–0.44)*	1.69 (1.65–1.74)*	0.32 (0.31–0.33)*
	Model 2 (*n* = 652,871)	Reference	0.36 (0.34–0.39)*	0.53 (0.51–0.54)*	1.41 (1.37–1.46)*	0.32 (0.31–0.33)*
At least one physical health condition	Model 1 (*n* = 652,880)	Reference	0.50 (0.48–0.52)*	0.85 (0.83–0.86)*	0.69 (0.67–0.71)*	0.28 (0.28–0.29)*
	Model 2 (*n* = 652,871)	Reference	0.62 (0.60–0.64)*	0.94 (0.92–0.96)*	0.82 (0.80–0.84)*	0.29 (0.29–0.30)*
Diabetes	Model 1 (*n* = 652,880)	Reference	1.46 (1.31–1.61)*	1.43 (1.33–1.53)*	0.93 (0.83–1.03)	0.41 (0.39–0.44)*
	Model 2 (*n* = 652,871)	Reference	1.30 (1.17–1.45)*	1.18 (1.10–1.27)*	1.21 (1.09–1.35)*	0.41 (0.39–0.44)*
Hypertension	Model 1 (*n* = 652,880)	Reference	0.60 (0.52–0.68)*	0.89 (0.82–0.95)	0.62 (0.55–0.69)*	0.30 (0.28–0.32)*
	Model 2 (*n* = 652,871)	Reference	0.67 (0.58–0.77)*	0.89 (0.82–0.95)	0.83 (0.74–0.93)	0.31 (0.29–0.33)*
Cardiac disease	Model 1 (*n* = 652,880)	Reference	0.50 (0.42–0.59)*	0.82 (0.75–0.90)*	0.84 (0.75–0.94)	0.41 (0.38–0.44)*
	Model 2 (*n* = 652,871)	Reference	0.61 (0.51–0.72)*	1.00 (0.91–1.09)	0.79 (0.70–0.89)*	0.42 (0.39–0.45)*
Thromboembolic condition	Model 1 (*n* = 652,880)	Reference	0.59 (0.49–0.70)*	0.95 (0.87–1.05)	0.69 (0.60–0.79)*	0.31 (0.29–0.34)*
	Model 2 (*n* = 652,871)	Reference	0.80 (0.66–0.96)	1.12 (1.02–1.24)	0.88 (0.76–1.02)	0.34 (0.31–0.37)*
Renal disease	Model 1 (*n* = 652,880)	Reference	0.79 (0.68–0.92)	1.02 (0.93–1.11)	0.87 (0.77–0.98)	0.61 (0.57–0.65)*
	Model 2 (*n* = 652,871)	Reference	0.97 (0.83–1.13)	1.25 (1.14–1.36)*	0.79 (0.69–0.89)*	0.63 (0.59–0.68)*
Hepatitis B	Model 1 (*n* = 652,880)	Reference	7.39 (6.12–8.90)*	5.88 (5.05–6.86)*	1.00 (0.69–1.40)	0.56 (0.45–0.69)*
	Model 2 (*n* = 652,871)	Reference	5.40 (4.43–6.58)*	4.44 (3.78–5.21)*	1.20 (0.83–1.69)	0.54 (0.44–0.67)*
Cancer	Model 1 (*n* = 652,880)	Reference	0.33 (0.20–0.49)*	0.62 (0.50–0.76)*	0.58 (0.44–0.76)*	0.31 (0.26–0.37)*
	Model 2 (*n* = 652,871)	Reference	0.53 (0.33–0.81)	0.77 (0.62–0.95)	0.87 (0.64–1.14)	0.35 (0.29–0.41)*
Inherited conditions	Model 1 (*n* = 652,880)	Reference	0.36 (0.31–0.41)*	0.71 (0.67–0.75)*	1.15 (1.07–1.22)*	0.59 (0.56–0.61)*
	Model 2 (*n* = 652,871)	Reference	0.44 (0.39–0.51)*	0.85 (0.80–0.91)*	1.08 (1.00–1.15)	0.61 (0.58–0.64)*
Family history of diabetes	Model 1 (*n* = 652,880)	Reference	1.13 (1.10–1.17)*	1.27 (1.24–1.29)*	0.97 (0.94–0.99)	0.26 (0.26–0.27)*
	Model 2 (*n* = 652,871)	Reference	0.89 (0.86–0.92)*	1.01 (0.99–1.03)	1.00 (0.97–1.02)	0.25 (0.25–0.26)*
Booking after 10 weeks gestation	Model 1 (*n* = 652,309)	Reference	2.66 (2.59–2.73)*	1.35 (1.33–1.38)*	1.44 (1.41–1.47)*	1.19 (1.18–1.21)*
	Model 2^ (*n* = 548,368)	Reference	2.25 (2.18–2.32)*	1.22 (1.20–1.24)*	1.38 (1.34–1.42)*	1.15 (1.14–1.17)*
Booking after 16 weeks gestation	Model 1 (*n* = 652,309)	Reference	5.04 (4.88–5.22)*	1.62 (1.57–1.67)*	2.43 (2.35–2.52)*	1.92 (1.88–1.96)*
	Model 2^ (*n* = 548,368)	Reference	3.69 (3.54–3.83)*	1.43 (1.38–1.48)*	2.23 (2.12–2.34)*	1.65 (1.61–1.70)*
Booking after 20 weeks gestation	Model 1 (*n* = 652,309)	Reference	4.69 (4.50–4.88)*	1.45 (1.39–1.51)*	2.39 (2.29–2.49)*	2.17 (2.11–2.22)*
	Model 2^ (*n* = 548,368)	Reference	3.69 (3.53–3.86)*	1.30 (1.24–1.35)*	2.13 (2.03–2.23)*	1.84 (1.78–1.89)*

### 3.4 Social factors

Probable migrants in vulnerable situations had higher odds of being unemployed and seeking work compared to probable non-migrants not in vulnerable situations in all models. Probable migrants in vulnerable situations had slightly lower odds of not having adequate support available during and after pregnancy in the unadjusted model (odds ratio (OR) 0.89, 95 % CI 0.82–0.96) but slightly increased odds in the fully adjusted model (adjusted OR 1.10, 95 % CI 1.02–1.19).

### 3.5 Reproductive health

Probable migrants in vulnerable situations had higher odds of previous obstetric complications (fully adjusted OR (aOR) 1.26, 95 % CI 1.21–1.31). They had lower odds of previous pregnancy loss in the unadjusted model (OR 0.91 95 % CI 0.88–0.95), but this difference reversed in the fully adjusted model (aOR 1.06, 95 % CI 1.02–1.11).

Probable migrants in vulnerable situations had higher odds of previous gestational diabetes mellitus and caesarean section in unadjusted and adjusted models, and lower odds of previous pre-eclampsia in unadjusted and adjusted models.

### 3.6 Health behaviours

Probable migrants in vulnerable situations had over double the odds of not taking folic acid supplements preconception compared to probable non-migrants not in vulnerable situations (aOR 2.15, 95 % CI 2.06–2.25).

Probable migrants in vulnerable situations had lower odds of smoking around conception in all models (aOR 0.86, 95 % CI 0.82–0.90), but those who smoked had higher odds of not quitting smoking during the year before pregnancy (aOR 1.56, 95 % CI 1.41–1.73).

### 3.7 Weight and pre-existing conditions

Probable migrants in vulnerable situations had increased odds of being underweight (aOR 1.55, 95 % CI 1.44–1.68). They had lower odds of overweight or obesity in all models (aOR 0.73, 95 % CI 0.71–0.75), and were less likely to have at least one mental health condition (aOR 0.36, 95 % CI 0.34–0.39) or physical health condition (aOR 0.62, 95 % CI 0.60–0.64).

Probable migrants in vulnerable situations had higher odds of diabetes (aOR 1.30, 95 % CI 1.17–1.45), and hepatitis b (aOR 5.40, 95 % CI 4.43–5.58), but lower odds of hypertension, cardiac disease, thromboembolic conditions, renal disease, cancer and inherited conditions in all models. Probable migrants in vulnerable situations had higher odds of family history of diabetes in the unadjusted model (OR 1.13, 95 % CI 1.10–1.17) but slightly reduced odds in the fully adjusted model (aOR 0.89, 95 % CI 0.86–0.92).

### 3.8 Late antenatal booking

Probable migrants in vulnerable situations had over double the odds of booking after 10 weeks gestation compared to probable non-migrants not in vulnerable situations (aOR 2.25, 95 % CI 2.18–2.32), and over three times the odds of booking after 16- and 20-weeks gestation (aOR 3.69, 95 % CI 3.54–3.83 and aOR 3.69, 95 % CI 3.53–3.86 respectively).

### 3.9 Sensitivity analysis

Excluding ethnicity as a covariate, participants with missing migration category and/or ethnicity, and participants who were under 20 and/or substance users (Models 3, 4 and 5 respectively in Supplementary Table 1) from regression analyses did not have a substantial influence on most preconception indicators. The exception to this included the preconception indicator of ‘no adequate support available during and after pregnancy’, for which model 2 (including adjustment for ethnicity) found very slightly higher odds for probable migrants in vulnerable situations, model 3 (without adjustment for ethnicity) found slightly lower odds, and models 4 (fully adjusted model excluding participants with missing migration category and/or ethnicity) and 5 (fully adjusted model excluding under 20 s and substance misusers) found no significant difference. Similarly, for ‘family history of diabetes’, only the models not adjusting for ethnicity (models 1 and 3) showed slightly higher odds of family history of diabetes for probable migrants in vulnerable situations, whereas those adjusting for ethnicity (models 2, 4 and 5) showed slightly lower odds. Slightly lower odds of previous pregnancy loss in unadjusted analysis reversed in adjusted analyses with increased odds in models adjusting for ethnicity (Models 2, 4 and 5) and no difference in odds in Model 3b. Lower odds of renal disease among probable migrants in unadjusted analysis and Model 3a became non-significant in models 2, 4 and 5.

## 4 Discussion

This is the first study to describe preconception indicators according to migration category in England. Women identified as probable migrants in vulnerable situations had over twice the odds of not taking folic acid before pregnancy and of having their first antenatal booking appointment after the recommended 10 weeks gestation (NICE, 2021) compared to probable non-migrants not in vulnerable situations, even after adjusting for confounding factors. This suggests that migration category has an independent association with these indicators. Probable migrants in vulnerable situations had increased odds of previous obstetric complications and of being underweight at booking, but lower odds of recorded physical and mental health conditions (apart from diabetes and hepatitis b), smoking and overweight or obesity. Findings highlight areas of interest for policy and practice aiming to reduce inequalities and improve migrant women's preconception health.

Higher odds of pre-existing diabetes among probable migrants indicates that diabetes is an area of particular importance for migrant women's preconception health. Ensuring women have good blood glucose control preconception and during pregnancy reduces risks of miscarriage, congenital anomalies, neonatal death and stillbirth (Tennant et al., 2014; Recommendations, 2022), and NICE guidelines emphasise the importance of pregnancy planning for women with diabetes (Recommendations, 2022). Just 17.5 % of women with type 1 diabetes and 38.4 % of women with type 2 diabetes have recommended HbA1c (blood glucose) levels in the first trimester, indicating inadequate diabetic control preconception (National Pregnancy in Diabetes Audit Report 2020 - NHS Digital, 2022). However, little is known about pregnancy planning among migrant women with diabetes.

Lower odds of folic acid supplementation preconception have also been identified among migrants to Ireland (Palmer et al., 2019) and Norway (Nilsen et al., 2019) and is a serious concern for migrant maternal health: maternal folate supplementation preconception is associated with fewer anomaly-related terminations and reduced neural tube defects (Daly et al., 2022). Moreover, being underweight preconception increases odds of small for gestational age, low birth weight and preterm birth (Caut et al., 2022).

Lower odds of many physical and mental health conditions among probable migrants may not be a positive finding: it could indicate lack of diagnosis due to, for example, lower primary care access rates among migrants compared to non-migrants (Zhang et al., 2022); lack of diagnosis could mean preconception care that could support a healthy pregnancy does not happen.

In contrast to our findings of lower odds of overweight or obesity among probable migrants, a Swedish population-based study found that individuals born in North Africa, Middle East and Sub-Saharan Africa were more likely to be obese during pregnancy than Swedish-born women (Henriksson et al., 2020). Migrants from countries with lower levels of obesity often gain unhealthy weight after migration (Murphy et al., 2017), which may explain lower levels of obesity among probable migrants in this study. Moreover, lower BMI levels are associated with type 2 diabetes among south Asian, black, Chinese and Arab populations (Caleyachetty et al., 2021). Thus, overweight and obesity remain important preconception risk factors in this population.

### 4.1 Strengths

A major strength of this study is the comprehensive nature of MSDS, being largely representative of the English population of birthing people (NHS Maternity Statistics, 2023). Migration is rarely recorded within electronic health records (Abubakar et al., 2018), so the presence of a CSF variable and ‘English as a first language’ within MSDS creates a unique opportunity to examine the influence of migration on preconception indicators, focusing on migrants in vulnerable situations.

### 4.2 Limitations

The main limitation of this study is missing data for migration category. However, sensitivity analysis undertaken during logistic regression analyses indicated that missing data did not have a substantial impact on outcomes. Missing data for preconception indicators, ranging from 0 % of records for pre-existing health conditions to 38.9 % of records with missing data for alcohol drinking at booking, may have impacted results. The true level of missing data relating to pre-existing conditions is likely to be higher because those with missing diagnosis data were recorded in the dataset as not having the diagnosis, so it was not possible for us to differentiate between missing data or true absence of a diagnosis.

For pre-existing health conditions, it is unclear whether lower prevalence rates in the probable migrant group for many conditions are because of true differences, underdiagnosis or non-disclosure (missing data were assumed to be ‘no’). Those with ‘missing’ migration category had the lowest odds of having mental or physical health conditions, indicating that in some participants’ records, there may be low reporting of variables not just related to migration category (either at an individual level, or systematic under-reporting at hospital, NHS Trust or IT-system level). Participants with the most deprived IMD decile had over double the proportion of missing migration category data compared to those in the least deprived, suggesting possible geographical variation in reporting. Further research is needed to further explore differences in mental and physical health conditions preconception between migrant women and UK-born women.

Uncertainty due to the method of identifying migrant women is a risk; for example, women who experience domestic abuse and do not speak English as their first language, but were born in the UK, would be identified as probable migrants in vulnerable situations. Similarly, UK-born women who do not speak English as their first language would be identified as ‘probable migrants in vulnerable situations’ if they were under 20 or misused alcohol and/or drugs. Sensitivity analysis was undertaken to exclude most of these individuals in logistic regression analyses, with no substantial effect on outcomes. Moreover, 21.3 % were identified as migrants, compared to 28.8 % of birthing people nationally (Office for National Statistics, 2022), indicating that we have likely identified many of the migrant women in this dataset. Finally, by including multiple preconception indicators and a large sample size, there is a risk of identifying false-positive (or negative) findings due to multiple testing. However, due to the large sample size of this study, levels of precision are high, with often considerable effect sizes that are clinically significant.

## 5 Conclusions

We have shown that migrant women in vulnerable situations are less likely to take folic acid during the preconception period than non-migrants, are more likely to have diabetes, hepatitis b and be underweight at booking, and are more likely to have had previous obstetric complications. They are also less likely to have their first antenatal appointment within the recommended 10 weeks. These findings highlight the opportunity for more comprehensive preconception care for migrant women in potentially vulnerable situations, who are already known to have worse perinatal outcomes (Heslehurst et al., 2018). Further research could explore inequalities at more granular levels of migration status (e.g. asylum seeker, refugee or region of origin), and identify barriers and facilitators to improving preconception health in this population.

## Data sharing statement

Data used within this study were collected for the national Maternity Services Dataset. Data are available on request to NHS Digital (https://digital.nhs.uk/services/data-access-request-service-dars).

## Funding

MM is supported by the 10.13039/501100007155Medical Research Council [Grant number MR/W01498X/1]. For the purpose of open access, the author has applied a Creative Commons Attribution (CC BY) licence. DS is supported by the National Institute for Health and Social Care Research (NIHR) Southampton Biomedical Research Centre [IS-BRC-1215-20004]. The views expressed are those of the author(s) and not necessarily those of MRC, UKRI, NIHR or the Department of Health and Social Care.

## CRediT authorship contribution statement

**Majel McGranahan:** Writing – review & editing, Writing – original draft, Validation, Project administration, Methodology, Investigation, Funding acquisition, Formal analysis, Data curation, Conceptualization. **Elizabeth Augarde:** Writing – review & editing, Formal analysis, Conceptualization. **Danielle Schoenaker:** Writing – review & editing, Formal analysis. **Helen Duncan:** Writing – review & editing, Validation, Formal analysis. **Sue Mann:** Writing – review & editing, Validation. **Debra Bick:** Writing – review & editing, Supervision. **Felicity Boardman:** Writing – review & editing, Supervision. **Oyinlola Oyebode:** Writing – review & editing, Validation, Supervision, Methodology, Funding acquisition, Conceptualization.

## Declaration of competing interest

The authors declare the following financial interests/personal relationships which may be considered as potential competing interests:

Majel McGranahan reports financial support was provided by UKRI Medical Research Council. Danielle Schoenaker reports financial support was provided by NIHR Southampton Biomedical Research Centre. Helen Duncan reports a relationship with Office for Health Improvement and Disparities that includes: employment. Debra Bick reports a relationship with MASIC foundation that includes: board membership. HD is National lead for lifecourse intelligence at the Office for Health Improvement and Disparities, Department of Health and Social Care If there are other authors, they declare that they have no known competing financial interests or personal relationships that could have appeared to influence the work reported in this paper.

## Appendix Supplementary materials

Image, application 1
