# Supplementary material for: Preconception health among migrant women in England: A cross-sectional analysis of maternity services data 2018–2019
Source: J Migr Health. 2024 Jul 27;10:100250. doi: 10.1016/j.jmh.2024.100250 (PMC11327935; doi:10.1016/j.jmh.2024.100250)
Supplement: Supplementary file 1 [file mmc1.docx]

**Supplementary Table 1: Full logistic regression models for all preconception health indicators**

Model 1 is unadjusted. Model 2 adjusts for most deprived Index of Multiple Deprivation (IMD) decile, mother’s age at booking and ethnicity. Model 3 adjusts for most deprived IMD decile and mother’s age at booking. Model 4 adjusts for most deprived IMD decile, mother’s age at booking and ethnicity but excludes those with missing data for complex social factors and/or English as a first language and/or ethnicity. Model 5 adjusts for most deprived IMD decile, mother’s age at booking and ethnicity but excludes those under age 20 and those with recorded substance misuse. Models marked with ‘^’ additionally adjust for number of previous live births. LCI = lower confidence interval. UCI = upper confidence interval. Complex social factors include women who misuse alcohol and/or drugs; women who are recent migrants, asylum seekers or refugees, or have difficulty reading or speaking English; women aged under 20; and/or women who experience domestic abuse.

| **Outcome: mother unemployed and seeking work** | | | | | | |
| --- | --- | --- | --- | --- | --- | --- |
| **Migration status** | **Odds ratio** | **95% LCI** | **95% UCI** | **P value** | **Number of observations** | **Pseudo R squared (Hosmer-Lemeshow)** |
| **Model 1** | | | | | | |
| Probable non-migrant not in vulnerable situations (English as first language without complex social factors) | Reference | Reference | Reference | Reference | 472,181 | 0.04 |
| Probable migrants in vulnerable situations (English not their first language and complex social factors) | 2.58 | 2.46 | 2.70 | 0.000 |  |  |
| Probable migrants not in vulnerable situations (only English not their first language) | 1.03 | 0.98 | 1.07 | 0.235 |  |  |
| Probable non-migrants in vulnerable situations (only complex social factors) | 5.10 | 4.92 | 5.28 | 0.000 |  |  |
| Missing migration status (first language or complex social factor data missing) | 1.09 | 1.05 | 1.13 | 0.000 |  |  |
| **Model 2** | | | | | | |
| Probable non-migrant not in vulnerable situations (English as first language without complex social factors) | Reference | Reference | Reference | Reference | 472,175 | 0.084 |
| Probable migrants in vulnerable situations (English not their first language and complex social factors) | 1.74 | 1.66 | 1.83 | 0.000 |  |  |
| Probable migrants not in vulnerable situations (only English not their first language) | 0.96 | 0.92 | 1.01 | 0.093 |  |  |
| Probable non-migrants in vulnerable situations (only complex social factors) | 2.84 | 2.73 | 2.95 | 0.000 |  |  |
| Missing migration status (first language or complex social factor data missing) | 1.03 | 0.99 | 1.07 | 0.116 |  |  |
| Not most deprived | Reference | Reference | Reference | Reference |  |  |
| Most deprived | 2.39 | 2.32 | 2.46 | 0.000 |  |  |
| Mother's age at booking (years) | 0.92 | 0.92 | 0.92 | 0.000 |  |  |
| White | Reference | Reference | Reference | Reference |  |  |
| Mixed | 1.56 | 1.44 | 1.68 | 0.000 |  |  |
| Asian | 1.07 | 1.02 | 1.12 | 0.003 |  |  |
| Black | 1.70 | 1.61 | 1.79 | 0.000 |  |  |
| Other | 1.25 | 1.16 | 1.33 | 0.000 |  |  |
| Missing ethnicity | 0.87 | 0.84 | 0.91 | 0.000 |  |  |
| **Model 3** | | | | | | |
| Probable non-migrant not in vulnerable situations (English as first language without complex social factors) | Reference | Reference | Reference | Reference | 472,175 | 0.082 |
| Probable migrants in vulnerable situations (English not their first language and complex social factors) | 1.81 | 1.72 | 1.90 | 0.000 |  |  |
| Probable migrants not in vulnerable situations (only English not their first language) | 0.99 | 0.95 | 1.03 | 0.570 |  |  |
| Probable non-migrants in vulnerable situations (only complex social factors) | 2.87 | 2.76 | 2.98 | 0.000 |  |  |
| Missing migration status (first language or complex social factor data missing) | 1.04 | 1.00 | 1.08 | 0.037 |  |  |
| Not most deprived | Reference | Reference | Reference | Reference |  |  |
| Most deprived | 2.44 | 2.37 | 2.51 | 0.000 |  |  |
| Mother's age at booking (years) | 0.92 | 0.92 | 0.93 | 0.000 |  |  |
| **Model 4** | | | | | | |
| Probable non-migrant not in vulnerable situations (English as first language without complex social factors) | Reference | Reference | Reference | Reference | 330,963 | 0.091 |
| Probable migrants in vulnerable situations (English not their first language and complex social factors) | 1.79 | 1.69 | 1.89 | 0.000 |  |  |
| Probable migrants not in vulnerable situations (only English not their first language) | 0.99 | 0.95 | 1.04 | 0.772 |  |  |
| Probable non-migrants in vulnerable situations (only complex social factors) | 2.86 | 2.75 | 2.98 | 0.000 |  |  |
| Not most deprived | Reference | Reference | Reference | Reference |  |  |
| Most deprived | 2.34 | 2.26 | 2.43 | 0.000 |  |  |
| Mother's age at booking (years) | 0.92 | 0.92 | 0.93 | 0.000 |  |  |
| White | Reference | Reference | Reference | Reference |  |  |
| Mixed | 1.56 | 1.43 | 1.70 | 0.000 |  |  |
| Asian | 0.94 | 0.89 | 0.98 | 0.011 |  |  |
| Black | 1.63 | 1.53 | 1.72 | 0.000 |  |  |
| Other | 1.14 | 1.06 | 1.23 | 0.001 |  |  |
| **Model 5** | | | | | | |
| Probable non-migrant not in vulnerable situations (English as first language without complex social factors) | Reference | Reference | Reference | Reference | 450,819 | 0.065 |
| Probable migrants in vulnerable situations (English not their first language and complex social factors) | 1.74 | 1.65 | 1.84 | 0.000 |  |  |
| Probable migrants not in vulnerable situations (only English not their first language) | 0.97 | 0.93 | 1.01 | 0.156 |  |  |
| Probable non-migrants in vulnerable situations (only complex social factors) | 3.11 | 2.96 | 3.27 | 0.000 |  |  |
| Missing migration status (first language or complex social factor data missing) | 0.95 | 0.91 | 0.98 | 0.006 |  |  |
| Not most deprived | Reference | Reference | Reference | Reference |  |  |
| Most deprived | 2.50 | 2.42 | 2.58 | 0.000 |  |  |
| Mother's age at booking (years) | 0.91 | 0.91 | 0.92 | 0.000 |  |  |
| White | Reference | Reference | Reference | Reference |  |  |
| Mixed | 1.66 | 1.52 | 1.80 | 0.000 |  |  |
| Asian | 1.07 | 1.02 | 1.12 | 0.006 |  |  |
| Black | 1.77 | 1.68 | 1.87 | 0.000 |  |  |
| Other | 1.29 | 1.20 | 1.39 | 0.000 |  |  |
| Missing ethnicity | 0.88 | 0.85 | 0.92 | 0.000 |  |  |

| **Outcome: no adequate support available during and after pregnancy** | | | | | | |
| --- | --- | --- | --- | --- | --- | --- |
| **Migration status** | **Odds ratio** | **95% LCI** | **95% UCI** | **P value** | **Number of observations** | **Pseudo R squared (Hosmer-Lemeshow)** |
| **Model 1** | | | | | | |
| Probable non-migrant not in vulnerable situations (English as first language without complex social factors) | Reference | Reference | Reference | Reference | 449,884 | 0.055 |
| Probable migrants in vulnerable situations (English not their first language and complex social factors) | 0.89 | 0.82 | 0.96 | 0.003 |  |  |
| Probable migrants not in vulnerable situations (only English not their first language) | 1.47 | 1.41 | 1.53 | 0.000 |  |  |
| Probable non-migrants in vulnerable situations (only complex social factors) | 1.36 | 1.29 | 1.44 | 0.000 |  |  |
| Missing migration status (first language or complex social factor data missing) | 4.46 | 4.33 | 4.59 | 0.000 |  |  |
| **Model 2** | | | | | | |
| Probable non-migrant not in vulnerable situations (English as first language without complex social factors) | Reference | Reference | Reference | Reference | 449,877 | 0.062 |
| Probable migrants in vulnerable situations (English not their first language and complex social factors) | 1.10 | 1.02 | 1.19 | 0.018 |  |  |
| Probable migrants not in vulnerable situations (only English not their first language) | 1.75 | 1.67 | 1.82 | 0.000 |  |  |
| Probable non-migrants in vulnerable situations (only complex social factors) | 1.37 | 1.29 | 1.45 | 0.000 |  |  |
| Missing migration status (first language or complex social factor data missing) | 4.56 | 4.43 | 4.69 | 0.000 |  |  |
| Not most deprived | Reference | Reference | Reference | Reference |  |  |
| Most deprived | 0.85 | 0.82 | 0.88 | 0.000 |  |  |
| Mother's age at booking (years) | 1.00 | 1.00 | 1.00 | 0.678 |  |  |
| White | Reference | Reference | Reference | Reference |  |  |
| Mixed | 0.73 | 0.66 | 0.81 | 0.000 |  |  |
| Asian | 0.44 | 0.41 | 0.46 | 0.000 |  |  |
| Black | 0.57 | 0.53 | 0.61 | 0.000 |  |  |
| Other | 0.63 | 0.58 | 0.68 | 0.000 |  |  |
| Missing ethnicity | 0.82 | 0.79 | 0.85 | 0.000 |  |  |
| **Model 3** | | | | | | |
| Probable non-migrant not in vulnerable situations (English as first language without complex social factors) | Reference | Reference | Reference | Reference | 449,877 | 0.056 |
| Probable migrants in vulnerable situations (English not their first language and complex social factors) | 0.91 | 0.84 | 0.99 | 0.022 |  |  |
| Probable migrants not in vulnerable situations (only English not their first language) | 1.49 | 1.43 | 1.55 | 0.000 |  |  |
| Probable non-migrants in vulnerable situations (only complex social factors) | 1.37 | 1.29 | 1.45 | 0.000 |  |  |
| Missing migration status (first language or complex social factor data missing) | 4.47 | 4.35 | 4.60 | 0.000 |  |  |
| Not most deprived | Reference | Reference | Reference | Reference |  |  |
| Most deprived | 0.82 | 0.78 | 0.85 | 0.000 |  |  |
| Mother's age at booking (years) | 1.00 | 0.99 | 1.00 | 0.005 |  |  |
| **Model 4** | | | | | | |
| Probable non-migrant not in vulnerable situations (English as first language without complex social factors) | Reference | Reference | Reference | Reference | 316,493 | 0.014 |
| Probable migrants in vulnerable situations (English not their first language and complex social factors) | 1.05 | 0.96 | 1.15 | 0.264 |  |  |
| Probable migrants not in vulnerable situations (only English not their first language) | 1.81 | 1.73 | 1.90 | 0.000 |  |  |
| Probable non-migrants in vulnerable situations (only complex social factors) | 1.45 | 1.36 | 1.54 | 0.000 |  |  |
| Not most deprived | Reference | Reference | Reference | Reference |  |  |
| Most deprived | 0.58 | 0.54 | 0.61 | 0.000 |  |  |
| Mother's age at booking (years) | 1.02 | 1.02 | 1.02 | 0.000 |  |  |
| White | Reference | Reference | Reference | Reference |  |  |
| Mixed | 0.86 | 0.76 | 0.98 | 0.020 |  |  |
| Asian | 0.50 | 0.47 | 0.53 | 0.000 |  |  |
| Black | 0.74 | 0.68 | 0.80 | 0.000 |  |  |
| Other | 0.60 | 0.54 | 0.66 | 0.000 |  |  |
| **Model 5** | | | | | | |
| Probable non-migrant not in vulnerable situations (English as first language without complex social factors) | Reference | Reference | Reference | Reference | 431,079 | 0.062 |
| Probable migrants in vulnerable situations (English not their first language and complex social factors) | 1.02 | 0.93 | 1.11 | 0.638 |  |  |
| Probable migrants not in vulnerable situations (only English not their first language) | 1.75 | 1.67 | 1.82 | 0.000 |  |  |
| Probable non-migrants in vulnerable situations (only complex social factors) | 1.33 | 1.23 | 1.43 | 0.000 |  |  |
| Missing migration status (first language or complex social factor data missing) | 4.49 | 4.36 | 4.62 | 0.000 |  |  |
| Not most deprived | Reference | Reference | Reference | Reference |  |  |
| Most deprived | 0.84 | 0.81 | 0.88 | 0.000 |  |  |
| Mother's age at booking (years) | 1.00 | 1.00 | 1.01 | 0.000 |  |  |
| White | Reference | Reference | Reference | Reference |  |  |
| Mixed | 0.74 | 0.66 | 0.82 | 0.000 |  |  |
| Asian | 0.44 | 0.41 | 0.46 | 0.000 |  |  |
| Black | 0.56 | 0.52 | 0.60 | 0.000 |  |  |
| Other | 0.63 | 0.58 | 0.68 | 0.000 |  |  |
| Missing ethnicity | 0.81 | 0.78 | 0.84 | 0.000 |  |  |

| **Outcome: previous obstetric complication** | | | | | | |
| --- | --- | --- | --- | --- | --- | --- |
| **Migration status** | **Odds ratio** | **95% LCI** | **95% UCI** | **P value** | **Number of observations** | **Pseudo R squared (Hosmer-Lemeshow)** |
| **Model 1** | | | | | | |
| Probable non-migrant not in vulnerable situations (English as first language without complex social factors) | Reference | Reference | Reference | Reference | 329,228 | 0.023 |
| Probable migrants in vulnerable situations (English not their first language and complex social factors) | 1.42 | 1.37 | 1.47 | 0.000 |  |  |
| Probable migrants not in vulnerable situations (only English not their first language) | 1.33 | 1.30 | 1.36 | 0.000 |  |  |
| Probable non-migrants in vulnerable situations (only complex social factors) | 0.87 | 0.84 | 0.90 | 0.000 |  |  |
| Missing migration status (first language or complex social factor data missing) | 0.44 | 0.43 | 0.45 | 0.000 |  |  |
| **Model 2^** | | | | | | |
| Probable non-migrant not in vulnerable situations (English as first language without complex social factors) | Reference | Reference | Reference | Reference | 325,619 | 0.041 |
| Probable migrants in vulnerable situations (English not their first language and complex social factors) | 1.26 | 1.21 | 1.31 | 0.000 |  |  |
| Probable migrants not in vulnerable situations (only English not their first language) | 1.18 | 1.15 | 1.21 | 0.000 |  |  |
| Probable non-migrants in vulnerable situations (only complex social factors) | 0.91 | 0.88 | 0.95 | 0.000 |  |  |
| Missing migration status (first language or complex social factor data missing) | 0.43 | 0.42 | 0.44 | 0.000 |  |  |
| Not most deprived | Reference | Reference | Reference | Reference |  |  |
| Most deprived | 1.09 | 1.06 | 1.11 | 0.000 |  |  |
| Mother's age at booking (years) | 1.03 | 1.03 | 1.03 | 0.000 |  |  |
| Number of previous live births | 1.22 | 1.21 | 1.23 | 0.000 |  |  |
| White | Reference | Reference | Reference | Reference |  |  |
| Mixed | 1.12 | 1.06 | 1.19 | 0.000 |  |  |
| Asian | 1.46 | 1.42 | 1.50 | 0.000 |  |  |
| Black | 1.21 | 1.17 | 1.25 | 0.000 |  |  |
| Other | 0.99 | 0.95 | 1.04 | 0.807 |  |  |
| Missing ethnicity | 0.94 | 0.91 | 0.96 | 0.000 |  |  |
| **Model 3^** | | | | | | |
| Probable non-migrant not in vulnerable situations (English as first language without complex social factors) | Reference | Reference | Reference | Reference | 325,619 | 0.039 |
| Probable migrants in vulnerable situations (English not their first language and complex social factors) | 1.35 | 1.30 | 1.40 | 0.000 |  |  |
| Probable migrants not in vulnerable situations (only English not their first language) | 1.27 | 1.24 | 1.31 | 0.000 |  |  |
| Probable non-migrants in vulnerable situations (only complex social factors) | 0.90 | 0.87 | 0.94 | 0.000 |  |  |
| Missing migration status (first language or complex social factor data missing) | 0.44 | 0.43 | 0.44 | 0.000 |  |  |
| Not most deprived | Reference | Reference | Reference | Reference |  |  |
| Most deprived | 1.11 | 1.08 | 1.14 | 0.000 |  |  |
| Mother's age at booking (years) | 1.03 | 1.03 | 1.03 | 0.000 |  |  |
| Number of previous live births | 1.23 | 1.22 | 1.23 | 0.000 |  |  |
| **Model 4^** | | | | | | |
| Probable non-migrant not in vulnerable situations (English as first language without complex social factors) | Reference | Reference | Reference | Reference | 216,001 | 0.028 |
| Probable migrants in vulnerable situations (English not their first language and complex social factors) | 1.22 | 1.17 | 1.27 | 0.000 |  |  |
| Probable migrants not in vulnerable situations (only English not their first language) | 1.17 | 1.14 | 1.20 | 0.000 |  |  |
| Probable non-migrants in vulnerable situations (only complex social factors) | 0.94 | 0.90 | 0.98 | 0.002 |  |  |
| Not most deprived | Reference | Reference | Reference | Reference |  |  |
| Most deprived | 1.15 | 1.12 | 1.19 | 0.000 |  |  |
| Mother's age at booking (years) | 1.03 | 1.03 | 1.03 | 0.000 |  |  |
| Number of previous live births | 1.24 | 1.23 | 1.25 | 0.000 |  |  |
| White | Reference | Reference | Reference | Reference |  |  |
| Mixed | 1.22 | 1.14 | 1.30 | 0.000 |  |  |
| Asian | 1.54 | 1.50 | 1.58 | 0.000 |  |  |
| Black | 1.32 | 1.27 | 1.37 | 0.000 |  |  |
| Other | 1.02 | 0.96 | 1.07 | 0.551 |  |  |
| **Model 5^** | | | | | | |
| Probable non-migrant not in vulnerable situations (English as first language without complex social factors) | Reference | Reference | Reference | Reference | 317,443 | 0.039 |
| Probable migrants in vulnerable situations (English not their first language and complex social factors) | 1.27 | 1.22 | 1.32 | 0.000 |  |  |
| Probable migrants not in vulnerable situations (only English not their first language) | 1.17 | 1.14 | 1.20 | 0.000 |  |  |
| Probable non-migrants in vulnerable situations (only complex social factors) | 0.97 | 0.93 | 1.01 | 0.168 |  |  |
| Missing migration status (first language or complex social factor data missing) | 0.44 | 0.43 | 0.45 | 0.000 |  |  |
| Not most deprived | Reference | Reference | Reference | Reference |  |  |
| Most deprived | 1.10 | 1.08 | 1.13 | 0.000 |  |  |
| Mother's age at booking (years) | 1.03 | 1.03 | 1.03 | 0.000 |  |  |
| Number of previous live births | 1.22 | 1.21 | 1.23 | 0.000 |  |  |
| White | Reference | Reference | Reference | Reference |  |  |
| Mixed | 1.13 | 1.06 | 1.20 | 0.000 |  |  |
| Asian | 1.47 | 1.43 | 1.51 | 0.000 |  |  |
| Black | 1.22 | 1.17 | 1.26 | 0.000 |  |  |
| Other | 0.99 | 0.95 | 1.04 | 0.754 |  |  |
| Missing ethnicity | 0.94 | 0.91 | 0.96 | 0.000 |  |  |

| **Outcome: previous pre-eclampsia, HELLP, eclampsia or gestational proteinuria** | | | | | | |
| --- | --- | --- | --- | --- | --- | --- |
| **Migration status** | **Odds ratio** | **95% LCI** | **95% UCI** | **P value** | **Number of observations** | **Pseudo R squared (Hosmer-Lemeshow)** |
| **Model 1** | | | | | | |
| Probable non-migrant not in vulnerable situations (English as first language without complex social factors) | Reference | Reference | Reference | Reference | 329,228 | 0.002 |
| Probable migrants in vulnerable situations (English not their first language and complex social factors) | 0.78 | 0.65 | 0.93 | 0.007 |  |  |
| Probable migrants not in vulnerable situations (only English not their first language) | 0.97 | 0.88 | 1.08 | 0.585 |  |  |
| Probable non-migrants in vulnerable situations (only complex social factors) | 0.70 | 0.59 | 0.83 | 0.000 |  |  |
| Missing migration status (first language or complex social factor data missing) | 0.69 | 0.63 | 0.75 | 0.000 |  |  |
| **Model 2^** | | | | | | |
| Probable non-migrant not in vulnerable situations (English as first language without complex social factors) | Reference | Reference | Reference | Reference | 325,619 | 0.012 |
| Probable migrants in vulnerable situations (English not their first language and complex social factors) | 0.69 | 0.57 | 0.83 | 0.000 |  |  |
| Probable migrants not in vulnerable situations (only English not their first language) | 0.94 | 0.84 | 1.04 | 0.250 |  |  |
| Probable non-migrants in vulnerable situations (only complex social factors) | 0.64 | 0.54 | 0.76 | 0.000 |  |  |
| Missing migration status (first language or complex social factor data missing) | 0.67 | 0.62 | 0.73 | 0.000 |  |  |
| Not most deprived | Reference | Reference | Reference | Reference |  |  |
| Most deprived | 1.32 | 1.20 | 1.44 | 0.000 |  |  |
| Mother's age at booking (years) | 1.00 | 0.99 | 1.01 | 0.827 |  |  |
| Number of previous live births | 1.21 | 1.18 | 1.24 | 0.000 |  |  |
| White | Reference | Reference | Reference | Reference |  |  |
| Mixed | 1.12 | 0.88 | 1.41 | 0.330 |  |  |
| Asian | 0.93 | 0.82 | 1.04 | 0.205 |  |  |
| Black | 1.58 | 1.39 | 1.79 | 0.000 |  |  |
| Other | 0.67 | 0.52 | 0.84 | 0.001 |  |  |
| Missing ethnicity | 1.09 | 0.98 | 1.21 | 0.119 |  |  |
| **Model 3^** | | | | | | |
| Probable non-migrant not in vulnerable situations (English as first language without complex social factors) | Reference | Reference | Reference | Reference | 325,619 | 0.01 |
| Probable migrants in vulnerable situations (English not their first language and complex social factors) | 0.68 | 0.57 | 0.82 | 0.000 |  |  |
| Probable migrants not in vulnerable situations (only English not their first language) | 0.93 | 0.84 | 1.03 | 0.161 |  |  |
| Probable non-migrants in vulnerable situations (only complex social factors) | 0.64 | 0.54 | 0.76 | 0.000 |  |  |
| Missing migration status (first language or complex social factor data missing) | 0.67 | 0.62 | 0.74 | 0.000 |  |  |
| Not most deprived | Reference | Reference | Reference | Reference |  |  |
| Most deprived | 1.34 | 1.23 | 1.46 | 0.000 |  |  |
| Mother's age at booking (years) | 1.00 | 1.00 | 1.01 | 0.560 |  |  |
| Number of previous live births | 1.21 | 1.18 | 1.24 | 0.000 |  |  |
| **Model 4^** | | | | | | |
| Probable non-migrant not in vulnerable situations (English as first language without complex social factors) | Reference | Reference | Reference | Reference | 216,001 | 0.013 |
| Probable migrants in vulnerable situations (English not their first language and complex social factors) | 0.70 | 0.57 | 0.85 | 0.000 |  |  |
| Probable migrants not in vulnerable situations (only English not their first language) | 0.95 | 0.85 | 1.07 | 0.410 |  |  |
| Probable non-migrants in vulnerable situations (only complex social factors) | 0.67 | 0.56 | 0.80 | 0.000 |  |  |
| Not most deprived | Reference | Reference | Reference | Reference |  |  |
| Most deprived | 1.33 | 1.20 | 1.48 | 0.000 |  |  |
| Mother's age at booking (years) | 1.00 | 1.00 | 1.01 | 0.213 |  |  |
| Number of previous live births | 1.21 | 1.17 | 1.24 | 0.000 |  |  |
| White | Reference | Reference | Reference | Reference |  |  |
| Mixed | 1.29 | 1.00 | 1.64 | 0.045 |  |  |
| Asian | 0.99 | 0.87 | 1.12 | 0.868 |  |  |
| Black | 1.82 | 1.59 | 2.08 | 0.000 |  |  |
| Other | 0.69 | 0.52 | 0.88 | 0.005 |  |  |
| **Model 5^** | | | | | | |
| Probable non-migrant not in vulnerable situations (English as first language without complex social factors) | Reference | Reference | Reference | Reference | 317,443 | 0.012 |
| Probable migrants in vulnerable situations (English not their first language and complex social factors) | 0.70 | 0.58 | 0.84 | 0.000 |  |  |
| Probable migrants not in vulnerable situations (only English not their first language) | 0.94 | 0.84 | 1.04 | 0.223 |  |  |
| Probable non-migrants in vulnerable situations (only complex social factors) | 0.58 | 0.47 | 0.70 | 0.000 |  |  |
| Missing migration status (first language or complex social factor data missing) | 0.69 | 0.63 | 0.75 | 0.000 |  |  |
| Not most deprived | Reference | Reference | Reference | Reference |  |  |
| Most deprived | 1.32 | 1.21 | 1.44 | 0.000 |  |  |
| Mother's age at booking (years) | 1.00 | 0.99 | 1.01 | 0.722 |  |  |
| Number of previous live births | 1.21 | 1.18 | 1.23 | 0.000 |  |  |
| White | Reference | Reference | Reference | Reference |  |  |
| Mixed | 1.14 | 0.89 | 1.43 | 0.272 |  |  |
| Asian | 0.94 | 0.83 | 1.05 | 0.271 |  |  |
| Black | 1.61 | 1.41 | 1.82 | 0.000 |  |  |
| Other | 0.67 | 0.52 | 0.84 | 0.001 |  |  |
| Missing ethnicity | 1.08 | 0.97 | 1.20 | 0.149 |  |  |

| **Outcome: previous gestational hypertension** | | | | | | |
| --- | --- | --- | --- | --- | --- | --- |
| **Migration status** | **Odds ratio** | **95% LCI** | **95% UCI** | **P value** | **Number of observations** | **Pseudo R squared (Hosmer-Lemeshow)** |
| **Model 1** | | | | | | |
| Probable non-migrant not in vulnerable situations (English as first language without complex social factors) | Reference | Reference | Reference | Reference | 329,228 | 0.007 |
| Probable migrants in vulnerable situations (English not their first language and complex social factors) | 1.02 | 0.89 | 1.16 | 0.768 |  |  |
| Probable migrants not in vulnerable situations (only English not their first language) | 0.91 | 0.83 | 0.99 | 0.027 |  |  |
| Probable non-migrants in vulnerable situations (only complex social factors) | 1.10 | 0.98 | 1.22 | 0.115 |  |  |
| Missing migration status (first language or complex social factor data missing) | 0.50 | 0.47 | 0.54 | 0.000 |  |  |
| **Model 2^** | | | | | | |
| Probable non-migrant not in vulnerable situations (English as first language without complex social factors) | Reference | Reference | Reference | Reference | 325,619 | 0.017 |
| Probable migrants in vulnerable situations (English not their first language and complex social factors) | 0.97 | 0.85 | 1.11 | 0.689 |  |  |
| Probable migrants not in vulnerable situations (only English not their first language) | 0.89 | 0.81 | 0.97 | 0.008 |  |  |
| Probable non-migrants in vulnerable situations (only complex social factors) | 1.07 | 0.95 | 1.20 | 0.243 |  |  |
| Missing migration status (first language or complex social factor data missing) | 0.50 | 0.47 | 0.55 | 0.000 |  |  |
| Not most deprived | Reference | Reference | Reference | Reference |  |  |
| Most deprived | 1.17 | 1.09 | 1.26 | 0.000 |  |  |
| Mother's age at booking (years) | 1.02 | 1.01 | 1.02 | 0.000 |  |  |
| Number of previous live births | 1.21 | 1.18 | 1.23 | 0.000 |  |  |
| White | Reference | Reference | Reference | Reference |  |  |
| Mixed | 0.98 | 0.80 | 1.19 | 0.827 |  |  |
| Asian | 1.02 | 0.93 | 1.11 | 0.676 |  |  |
| Black | 1.12 | 1.00 | 1.25 | 0.051 |  |  |
| Other | 0.75 | 0.62 | 0.89 | 0.001 |  |  |
| Missing ethnicity | 0.70 | 0.63 | 0.77 | 0.000 |  |  |
| **Model 3^** | | | | | | |
| Probable non-migrant not in vulnerable situations (English as first language without complex social factors) | Reference | Reference | Reference | Reference | 325,619 | 0.016 |
| Probable migrants in vulnerable situations (English not their first language and complex social factors) | 1.08 | 0.95 | 1.23 | 0.257 |  |  |
| Probable migrants not in vulnerable situations (only English not their first language) | 1.15 | 1.06 | 1.25 | 0.001 |  |  |
| Probable non-migrants in vulnerable situations (only complex social factors) | 0.93 | 0.83 | 1.05 | 0.219 |  |  |
| Missing migration status (first language or complex social factor data missing) | 2.00 | 1.85 | 2.17 | 0.000 |  |  |
| Not most deprived | Reference | Reference | Reference | Reference |  |  |
| Most deprived | 0.85 | 0.79 | 0.92 | 0.000 |  |  |
| Mother's age at booking (years) | 0.98 | 0.98 | 0.99 | 0.000 |  |  |
| Number of previous live births | 0.82 | 0.81 | 0.84 | 0.000 |  |  |
| **Model 4^** | | | | | | |
| Probable non-migrant not in vulnerable situations (English as first language without complex social factors) | Reference | Reference | Reference | Reference | 216,001 | 0.011 |
| Probable migrants in vulnerable situations (English not their first language and complex social factors) | 0.96 | 0.83 | 1.11 | 0.620 |  |  |
| Probable migrants not in vulnerable situations (only English not their first language) | 0.89 | 0.81 | 0.98 | 0.019 |  |  |
| Probable non-migrants in vulnerable situations (only complex social factors) | 1.06 | 0.94 | 1.19 | 0.355 |  |  |
| Not most deprived | Reference | Reference | Reference | Reference |  |  |
| Most deprived | 1.21 | 1.11 | 1.31 | 0.000 |  |  |
| Mother's age at booking (years) | 1.02 | 1.01 | 1.02 | 0.000 |  |  |
| Number of previous live births | 1.21 | 1.18 | 1.24 | 0.000 |  |  |
| White | Reference | Reference | Reference | Reference |  |  |
| Mixed | 1.04 | 0.84 | 1.28 | 0.698 |  |  |
| Asian | 1.03 | 0.93 | 1.13 | 0.542 |  |  |
| Black | 1.17 | 1.03 | 1.32 | 0.013 |  |  |
| Other | 0.76 | 0.63 | 0.92 | 0.005 |  |  |
| **Model 5^** | | | | | | |
| Probable non-migrant not in vulnerable situations (English as first language without complex social factors) | Reference | Reference | Reference | Reference | 317,443 | 0.017 |
| Probable migrants in vulnerable situations (English not their first language and complex social factors) | 1.00 | 0.87 | 1.14 | 0.946 |  |  |
| Probable migrants not in vulnerable situations (only English not their first language) | 0.89 | 0.81 | 0.97 | 0.007 |  |  |
| Probable non-migrants in vulnerable situations (only complex social factors) | 1.17 | 1.04 | 1.32 | 0.009 |  |  |
| Missing migration status (first language or complex social factor data missing) | 0.52 | 0.48 | 0.56 | 0.000 |  |  |
| Not most deprived | Reference | Reference | Reference | Reference |  |  |
| Most deprived | 1.19 | 1.10 | 1.28 | 0.000 |  |  |
| Mother's age at booking (years) | 1.02 | 1.01 | 1.02 | 0.000 |  |  |
| Number of previous live births | 1.20 | 1.18 | 1.23 | 0.000 |  |  |
| White | Reference | Reference | Reference | Reference |  |  |
| Mixed | 0.99 | 0.81 | 1.20 | 0.936 |  |  |
| Asian | 1.02 | 0.93 | 1.11 | 0.673 |  |  |
| Black | 1.12 | 1.00 | 1.26 | 0.044 |  |  |
| Other | 0.75 | 0.63 | 0.90 | 0.002 |  |  |
| Missing ethnicity | 0.69 | 0.62 | 0.76 | 0.000 |  |  |

| **Outcome: previous gestational diabetes** | | | | | | |
| --- | --- | --- | --- | --- | --- | --- |
| **Migration status** | **Odds ratio** | **95% LCI** | **95% UCI** | **P value** | **Number of observations** | **Pseudo R squared (Hosmer-Lemeshow)** |
| **Model 1** | | | | | | |
| Probable non-migrant not in vulnerable situations (English as first language without complex social factors) | Reference | Reference | Reference | Reference | 329,228 | 0.017 |
| Probable migrants in vulnerable situations (English not their first language and complex social factors) | 1.64 | 1.49 | 1.80 | 0.000 |  |  |
| Probable migrants not in vulnerable situations (only English not their first language) | 1.97 | 1.86 | 2.09 | 0.000 |  |  |
| Probable non-migrants in vulnerable situations (only complex social factors) | 0.63 | 0.55 | 0.71 | 0.000 |  |  |
| Missing migration status (first language or complex social factor data missing) | 0.58 | 0.54 | 0.62 | 0.000 |  |  |
| **Model 2^** | | | | | | |
| Probable non-migrant not in vulnerable situations (English as first language without complex social factors) | Reference | Reference | Reference | Reference | 325,619 | 0.053 |
| Probable migrants in vulnerable situations (English not their first language and complex social factors) | 1.12 | 1.01 | 1.24 | 0.024 |  |  |
| Probable migrants not in vulnerable situations (only English not their first language) | 1.36 | 1.28 | 1.45 | 0.000 |  |  |
| Probable non-migrants in vulnerable situations (only complex social factors) | 0.68 | 0.59 | 0.77 | 0.000 |  |  |
| Missing migration status (first language or complex social factor data missing) | 0.55 | 0.51 | 0.59 | 0.000 |  |  |
| Not most deprived | Reference | Reference | Reference | Reference |  |  |
| Most deprived | 1.12 | 1.05 | 1.19 | 0.000 |  |  |
| Mother's age at booking (years) | 1.04 | 1.03 | 1.04 | 0.000 |  |  |
| Number of previous live births | 1.24 | 1.22 | 1.26 | 0.000 |  |  |
| White | Reference | Reference | Reference | Reference |  |  |
| Mixed | 1.26 | 1.06 | 1.49 | 0.008 |  |  |
| Asian | 2.97 | 2.80 | 3.15 | 0.000 |  |  |
| Black | 1.51 | 1.37 | 1.66 | 0.000 |  |  |
| Other | 1.37 | 1.21 | 1.55 | 0.000 |  |  |
| Missing ethnicity | 1.09 | 1.01 | 1.19 | 0.030 |  |  |
| **Model 3^** | | | | | | |
| Probable non-migrant not in vulnerable situations (English as first language without complex social factors) | Reference | Reference | Reference | Reference | 325,619 | 0.035 |
| Probable migrants in vulnerable situations (English not their first language and complex social factors) | 1.49 | 1.35 | 1.64 | 0.000 |  |  |
| Probable migrants not in vulnerable situations (only English not their first language) | 1.86 | 1.75 | 1.97 | 0.000 |  |  |
| Probable non-migrants in vulnerable situations (only complex social factors) | 0.65 | 0.56 | 0.73 | 0.000 |  |  |
| Missing migration status (first language or complex social factor data missing) | 0.57 | 0.53 | 0.61 | 0.000 |  |  |
| Not most deprived | Reference | Reference | Reference | Reference |  |  |
| Most deprived | 1.20 | 1.13 | 1.28 | 0.000 |  |  |
| Mother's age at booking (years) | 1.04 | 1.04 | 1.05 | 0.000 |  |  |
| Number of previous live births | 1.24 | 1.22 | 1.26 | 0.000 |  |  |
| **Model 4^** | | | | | | |
| Probable non-migrant not in vulnerable situations (English as first language without complex social factors) | Reference | Reference | Reference | Reference | 216,001 | 0.051 |
| Probable migrants in vulnerable situations (English not their first language and complex social factors) | 1.11 | 0.99 | 1.23 | 0.065 |  |  |
| Probable migrants not in vulnerable situations (only English not their first language) | 1.36 | 1.27 | 1.45 | 0.000 |  |  |
| Probable non-migrants in vulnerable situations (only complex social factors) | 0.69 | 0.60 | 0.79 | 0.000 |  |  |
| Not most deprived | Reference | Reference | Reference | Reference |  |  |
| Most deprived | 1.10 | 1.03 | 1.18 | 0.007 |  |  |
| Mother's age at booking (years) | 1.04 | 1.03 | 1.04 | 0.000 |  |  |
| Number of previous live births | 1.24 | 1.22 | 1.27 | 0.000 |  |  |
| White | Reference | Reference | Reference | Reference |  |  |
| Mixed | 1.34 | 1.11 | 1.60 | 0.002 |  |  |
| Asian | 3.01 | 2.82 | 3.21 | 0.000 |  |  |
| Black | 1.58 | 1.43 | 1.75 | 0.000 |  |  |
| Other | 1.30 | 1.13 | 1.49 | 0.000 |  |  |
| **Model 5^** | | | | | | |
| Probable non-migrant not in vulnerable situations (English as first language without complex social factors) | Reference | Reference | Reference | Reference | 317,443 | 0.052 |
| Probable migrants in vulnerable situations (English not their first language and complex social factors) | 1.13 | 1.02 | 1.24 | 0.019 |  |  |
| Probable migrants not in vulnerable situations (only English not their first language) | 1.36 | 1.28 | 1.45 | 0.000 |  |  |
| Probable non-migrants in vulnerable situations (only complex social factors) | 0.68 | 0.59 | 0.78 | 0.000 |  |  |
| Missing migration status (first language or complex social factor data missing) | 0.57 | 0.53 | 0.61 | 0.000 |  |  |
| Not most deprived | Reference | Reference | Reference | Reference |  |  |
| Most deprived | 1.13 | 1.06 | 1.20 | 0.000 |  |  |
| Mother's age at booking (years) | 1.04 | 1.03 | 1.04 | 0.000 |  |  |
| Number of previous live births | 1.24 | 1.22 | 1.26 | 0.000 |  |  |
| White | Reference | Reference | Reference | Reference |  |  |
| Mixed | 1.27 | 1.07 | 1.50 | 0.006 |  |  |
| Asian | 2.98 | 2.81 | 3.16 | 0.000 |  |  |
| Black | 1.52 | 1.38 | 1.67 | 0.000 |  |  |
| Other | 1.37 | 1.21 | 1.55 | 0.000 |  |  |
| Missing ethnicity | 1.10 | 1.01 | 1.19 | 0.028 |  |  |

| **Outcome: previous caesarean section** | | | | | | |
| --- | --- | --- | --- | --- | --- | --- |
| **Migration status** | **Odds ratio** | **95% LCI** | **95% UCI** | **P value** | **Number of observations** | **Pseudo R squared (Hosmer-Lemeshow)** |
| **Model 1** | | | | | | |
| Probable non-migrant not in vulnerable situations (English as first language without complex social factors) | Reference | Reference | Reference | Reference | 306,429 | 0.001 |
| Probable migrants in vulnerable situations (English not their first language and complex social factors) | 1.23 | 1.18 | 1.28 | 0.000 |  |  |
| Probable migrants not in vulnerable situations (only English not their first language) | 1.21 | 1.18 | 1.24 | 0.000 |  |  |
| Probable non-migrants in vulnerable situations (only complex social factors) | 0.86 | 0.82 | 0.89 | 0.000 |  |  |
| Missing migration status (first language or complex social factor data missing) | 0.98 | 0.96 | 1.00 | 0.055 |  |  |
| **Model 2^** | | | | | | |
| Probable non-migrant not in vulnerable situations (English as first language without complex social factors) | Reference | Reference | Reference | Reference | 298,229 | 0.02 |
| Probable migrants in vulnerable situations (English not their first language and complex social factors) | 1.13 | 1.08 | 1.18 | 0.000 |  |  |
| Probable migrants not in vulnerable situations (only English not their first language) | 1.07 | 1.04 | 1.10 | 0.000 |  |  |
| Probable non-migrants in vulnerable situations (only complex social factors) | 1.02 | 0.98 | 1.07 | 0.363 |  |  |
| Missing migration status (first language or complex social factor data missing) | 0.97 | 0.95 | 0.99 | 0.002 |  |  |
| Not most deprived | Reference | Reference | Reference | Reference |  |  |
| Most deprived | 0.98 | 0.95 | 1.00 | 0.068 |  |  |
| Mother's age at booking (years) | 1.05 | 1.04 | 1.05 | 0.000 |  |  |
| Number of previous live births | 1.14 | 1.13 | 1.15 | 0.000 |  |  |
| White | Reference | Reference | Reference | Reference |  |  |
| Mixed | 1.03 | 0.97 | 1.10 | 0.372 |  |  |
| Asian | 1.36 | 1.32 | 1.40 | 0.000 |  |  |
| Black | 1.42 | 1.36 | 1.47 | 0.000 |  |  |
| Other | 1.22 | 1.16 | 1.28 | 0.000 |  |  |
| Missing ethnicity | 1.05 | 1.02 | 1.08 | 0.000 |  |  |
| **Model 3^** | | | | | | |
| Probable non-migrant not in vulnerable situations (English as first language without complex social factors) | Reference | Reference | Reference | Reference | 298,229 | 0.017 |
| Probable migrants in vulnerable situations (English not their first language and complex social factors) | 1.24 | 1.19 | 1.29 | 0.000 |  |  |
| Probable migrants not in vulnerable situations (only English not their first language) | 1.16 | 1.13 | 1.19 | 0.000 |  |  |
| Probable non-migrants in vulnerable situations (only complex social factors) | 1.02 | 0.97 | 1.06 | 0.472 |  |  |
| Missing migration status (first language or complex social factor data missing) | 0.98 | 0.96 | 1.00 | 0.037 |  |  |
| Not most deprived | Reference | Reference | Reference | Reference |  |  |
| Most deprived | 1.01 | 0.98 | 1.03 | 0.637 |  |  |
| Mother's age at booking (years) | 1.05 | 1.05 | 1.05 | 0.000 |  |  |
| Number of previous live births | 1.14 | 1.13 | 1.15 | 0.000 |  |  |
| **Model 4^** | | | | | | |
| Probable non-migrant not in vulnerable situations (English as first language without complex social factors) | Reference | Reference | Reference | Reference | 208,684 | 0.021 |
| Probable migrants in vulnerable situations (English not their first language and complex social factors) | 1.11 | 1.06 | 1.16 | 0.000 |  |  |
| Probable migrants not in vulnerable situations (only English not their first language) | 1.07 | 1.04 | 1.10 | 0.000 |  |  |
| Probable non-migrants in vulnerable situations (only complex social factors) | 0.97 | 0.93 | 1.01 | 0.165 |  |  |
| Not most deprived | Reference | Reference | Reference | Reference |  |  |
| Most deprived | 1.00 | 0.97 | 1.03 | 0.883 |  |  |
| Mother's age at booking (years) | 1.05 | 1.04 | 1.05 | 0.000 |  |  |
| Number of previous live births | 1.14 | 1.13 | 1.15 | 0.000 |  |  |
| White | Reference | Reference | Reference | Reference |  |  |
| Mixed | 1.02 | 0.95 | 1.10 | 0.498 |  |  |
| Asian | 1.35 | 1.31 | 1.39 | 0.000 |  |  |
| Black | 1.29 | 1.23 | 1.34 | 0.000 |  |  |
| Other | 1.17 | 1.11 | 1.24 | 0.000 |  |  |
| **Model 5^** |  |  |  |  |  |  |
| Probable non-migrant not in vulnerable situations (English as first language without complex social factors) | Reference | Reference | Reference | Reference | 298,229 | 0.02 |
| Probable migrants in vulnerable situations (English not their first language and complex social factors) | 1.13 | 1.08 | 1.18 | 0.000 |  |  |
| Probable migrants not in vulnerable situations (only English not their first language) | 1.07 | 1.04 | 1.10 | 0.000 |  |  |
| Probable non-migrants in vulnerable situations (only complex social factors) | 1.02 | 0.98 | 1.07 | 0.363 |  |  |
| Missing migration status (first language or complex social factor data missing) | 0.97 | 0.95 | 0.99 | 0.002 |  |  |
| Not most deprived | Reference | Reference | Reference | Reference |  |  |
| Most deprived | 0.98 | 0.95 | 1.00 | 0.068 |  |  |
| Mother's age at booking (years) | 1.05 | 1.04 | 1.05 | 0.000 |  |  |
| Number of previous live births | 1.14 | 1.13 | 1.15 | 0.000 |  |  |
| White | Reference | Reference | Reference | Reference |  |  |
| Mixed | 1.03 | 0.97 | 1.10 | 0.372 |  |  |
| Asian | 1.36 | 1.32 | 1.40 | 0.000 |  |  |
| Black | 1.42 | 1.36 | 1.47 | 0.000 |  |  |
| Other | 1.22 | 1.16 | 1.28 | 0.000 |  |  |
| Missing ethnicity | 1.05 | 1.02 | 1.08 | 0.000 |  |  |

| **Outcome: previous pregnancy loss** | | | | | | |
| --- | --- | --- | --- | --- | --- | --- |
| **Migration status** | **Odds ratio** | **95% LCI** | **95% UCI** | **P value** | **Number of observations** | **Pseudo R squared (Hosmer-Lemeshow)** |
| **Model 1** | | | | | | |
| Probable non-migrant not in vulnerable situations (English as first language without complex social factors) | Reference | Reference | Reference | Reference | 301,168 | 0.008 |
| Probable migrants in vulnerable situations (English not their first language and complex social factors) | 0.91 | 0.88 | 0.95 | 0.000 |  |  |
| Probable migrants not in vulnerable situations (only English not their first language) | 0.79 | 0.77 | 0.81 | 0.000 |  |  |
| Probable non-migrants in vulnerable situations (only complex social factors) | 1.63 | 1.58 | 1.69 | 0.000 |  |  |
| Missing migration status (first language or complex social factor data missing) | 1.45 | 1.43 | 1.48 | 0.000 |  |  |
| **Model 2^** | | | | | | |
| Probable non-migrant not in vulnerable situations (English as first language without complex social factors) | Reference | Reference | Reference | Reference | 289,853 | 0.032 |
| Probable migrants in vulnerable situations (English not their first language and complex social factors) | 1.06 | 1.02 | 1.11 | 0.004 |  |  |
| Probable migrants not in vulnerable situations (only English not their first language) | 0.85 | 0.83 | 0.87 | 0.000 |  |  |
| Probable non-migrants in vulnerable situations (only complex social factors) | 1.78 | 1.72 | 1.86 | 0.000 |  |  |
| Missing migration status (first language or complex social factor data missing) | 1.45 | 1.42 | 1.47 | 0.000 |  |  |
| Not most deprived | Reference | Reference | Reference | Reference |  |  |
| Most deprived | 1.13 | 1.11 | 1.16 | 0.000 |  |  |
| Mother's age at booking (years) | 1.03 | 1.02 | 1.03 | 0.000 |  |  |
| Number of previous live births | 0.69 | 0.69 | 0.70 | 0.000 |  |  |
| White | Reference | Reference | Reference | Reference |  |  |
| Mixed | 1.23 | 1.16 | 1.29 | 0.000 |  |  |
| Asian | 0.75 | 0.73 | 0.77 | 0.000 |  |  |
| Black | 1.16 | 1.12 | 1.20 | 0.000 |  |  |
| Other | 0.93 | 0.89 | 0.97 | 0.002 |  |  |
| Missing ethnicity | 0.87 | 0.85 | 0.89 | 0.000 |  |  |
| **Model 3^** | | | | | | |
| Probable non-migrant not in vulnerable situations (English as first language without complex social factors) | Reference | Reference | Reference | Reference | 289,853 | 0.03 |
| Probable migrants in vulnerable situations (English not their first language and complex social factors) | 1.00 | 0.96 | 1.04 | 0.923 |  |  |
| Probable migrants not in vulnerable situations (only English not their first language) | 0.80 | 0.78 | 0.82 | 0.000 |  |  |
| Probable non-migrants in vulnerable situations (only complex social factors) | 1.80 | 1.73 | 1.88 | 0.000 |  |  |
| Missing migration status (first language or complex social factor data missing) | 1.43 | 1.40 | 1.46 | 0.000 |  |  |
| Not most deprived | Reference | Reference | Reference | Reference |  |  |
| Most deprived | 1.13 | 1.10 | 1.15 | 0.000 |  |  |
| Mother's age at booking (years) | 1.02 | 1.02 | 1.03 | 0.000 |  |  |
| Number of previous live births | 0.69 | 0.69 | 0.70 | 0.000 |  |  |
| **Model 4^** | | | | | | |
| Probable non-migrant not in vulnerable situations (English as first language without complex social factors) | Reference | Reference | Reference | Reference | 197,699 | 0.033 |
| Probable migrants in vulnerable situations (English not their first language and complex social factors) | 1.13 | 1.08 | 1.18 | 0.000 |  |  |
| Probable migrants not in vulnerable situations (only English not their first language) | 0.85 | 0.83 | 0.88 | 0.000 |  |  |
| Probable non-migrants in vulnerable situations (only complex social factors) | 1.86 | 1.79 | 1.94 | 0.000 |  |  |
| Not most deprived | Reference | Reference | Reference | Reference |  |  |
| Most deprived | 1.20 | 1.17 | 1.24 | 0.000 |  |  |
| Mother's age at booking (years) | 1.03 | 1.03 | 1.03 | 0.000 |  |  |
| Number of previous live births | 0.67 | 0.66 | 0.67 | 0.000 |  |  |
| White | Reference | Reference | Reference | Reference |  |  |
| Mixed | 1.28 | 1.20 | 1.36 | 0.000 |  |  |
| Asian | 0.77 | 0.75 | 0.80 | 0.000 |  |  |
| Black | 1.18 | 1.13 | 1.23 | 0.000 |  |  |
| Other | 0.91 | 0.87 | 0.96 | 0.001 |  |  |
| **Model 5^** | | | | | | |
| Probable non-migrant not in vulnerable situations (English as first language without complex social factors) | Reference | Reference | Reference | Reference | 289,853 | 0.032 |
| Probable migrants in vulnerable situations (English not their first language and complex social factors) | 1.06 | 1.02 | 1.11 | 0.004 |  |  |
| Probable migrants not in vulnerable situations (only English not their first language) | 0.85 | 0.83 | 0.87 | 0.000 |  |  |
| Probable non-migrants in vulnerable situations (only complex social factors) | 1.78 | 1.72 | 1.86 | 0.000 |  |  |
| Missing migration status (first language or complex social factor data missing) | 1.45 | 1.42 | 1.47 | 0.000 |  |  |
| Not most deprived | Reference | Reference | Reference | Reference |  |  |
| Most deprived | 1.13 | 1.11 | 1.16 | 0.000 |  |  |
| Mother's age at booking (years) | 1.03 | 1.02 | 1.03 | 0.000 |  |  |
| Number of previous live births | 0.69 | 0.69 | 0.70 | 0.000 |  |  |
| White | Reference | Reference | Reference | Reference |  |  |
| Mixed | 1.23 | 1.16 | 1.29 | 0.000 |  |  |
| Asian | 0.75 | 0.73 | 0.77 | 0.000 |  |  |
| Black | 1.16 | 1.12 | 1.20 | 0.000 |  |  |
| Other | 0.93 | 0.89 | 0.97 | 0.002 |  |  |
| Missing ethnicity | 0.87 | 0.85 | 0.89 | 0.000 |  |  |

| **Outcome: not taking folic acid before conception** | | | | | | |
| --- | --- | --- | --- | --- | --- | --- |
| **Migration status** | **Odds ratio** | **95% LCI** | **95% UCI** | **P value** | **Number of observations** | **Pseudo R squared (Hosmer-Lemeshow)** |
| **Model 1** | | | | | | |
| Probable non-migrant not in vulnerable situations (English as first language without complex social factors) | Reference | Reference | Reference | Reference | 488,987 | 0.012 |
| Probable migrants in vulnerable situations (English not their first language and complex social factors) | 3.04 | 2.92 | 3.16 | 0.000 |  |  |
| Probable migrants not in vulnerable situations (only English not their first language) | 1.29 | 1.27 | 1.32 | 0.000 |  |  |
| Probable non-migrants in vulnerable situations (only complex social factors) | 2.38 | 2.31 | 2.46 | 0.000 |  |  |
| Missing migration status (first language or complex social factor data missing) | 1.06 | 1.05 | 1.08 | 0.000 |  |  |
| **Model 2^** |  |  |  |  |  |  |
| Probable non-migrant not in vulnerable situations (English as first language without complex social factors) | Reference | Reference | Reference | Reference | 422,527 | 0.073 |
| Probable migrants in vulnerable situations (English not their first language and complex social factors) | 2.15 | 2.06 | 2.25 | 0.000 |  |  |
| Probable migrants not in vulnerable situations (only English not their first language) | 1.22 | 1.19 | 1.25 | 0.000 |  |  |
| Probable non-migrants in vulnerable situations (only complex social factors) | 1.49 | 1.44 | 1.55 | 0.000 |  |  |
| Missing migration status (first language or complex social factor data missing) | 1.09 | 1.07 | 1.11 | 0.000 |  |  |
| Not most deprived | Reference | Reference | Reference | Reference |  |  |
| Most deprived | 1.67 | 1.63 | 1.71 | 0.000 |  |  |
| Mother's age at booking (years) | 0.91 | 0.91 | 0.92 | 0.000 |  |  |
| Number of previous live births | 1.54 | 1.52 | 1.55 | 0.000 |  |  |
| White | Reference | Reference | Reference | Reference |  |  |
| Mixed | 1.36 | 1.29 | 1.44 | 0.000 |  |  |
| Asian | 1.43 | 1.40 | 1.47 | 0.000 |  |  |
| Black | 1.95 | 1.87 | 2.03 | 0.000 |  |  |
| Other | 1.39 | 1.33 | 1.45 | 0.000 |  |  |
| Missing ethnicity | 1.18 | 1.15 | 1.20 | 0.000 |  |  |
| **Model 3^** | | | | | | |
| Probable non-migrant not in vulnerable situations (English as first language without complex social factors) | Reference | Reference | Reference | Reference | 422,527 | 0.069 |
| Probable migrants in vulnerable situations (English not their first language and complex social factors) | 2.41 | 2.30 | 2.51 | 0.000 |  |  |
| Probable migrants not in vulnerable situations (only English not their first language) | 1.32 | 1.30 | 1.35 | 0.000 |  |  |
| Probable non-migrants in vulnerable situations (only complex social factors) | 1.50 | 1.45 | 1.56 | 0.000 |  |  |
| Missing migration status (first language or complex social factor data missing) | 1.09 | 1.07 | 1.11 | 0.000 |  |  |
| Not most deprived | Reference | Reference | Reference | Reference |  |  |
| Most deprived | 1.74 | 1.69 | 1.78 | 0.000 |  |  |
| Mother's age at booking (years) | 0.92 | 0.92 | 0.92 | 0.000 |  |  |
| Number of previous live births | 1.54 | 1.52 | 1.55 | 0.000 |  |  |
| **Model 4^** |  |  |  |  |  |  |
| Probable non-migrant not in vulnerable situations (English as first language without complex social factors) | Reference | Reference | Reference | Reference | 289,190 | 0.073 |
| Probable migrants in vulnerable situations (English not their first language and complex social factors) | 2.18 | 2.07 | 2.29 | 0.000 |  |  |
| Probable migrants not in vulnerable situations (only English not their first language) | 1.20 | 1.18 | 1.23 | 0.000 |  |  |
| Probable non-migrants in vulnerable situations (only complex social factors) | 1.23 | 1.18 | 1.28 | 0.000 |  |  |
| Not most deprived | Reference | Reference | Reference | Reference |  |  |
| Most deprived | 1.62 | 1.57 | 1.67 | 0.000 |  |  |
| Mother's age at booking (years) | 0.91 | 0.91 | 0.91 | 0.000 |  |  |
| Number of previous live births | 1.57 | 1.55 | 1.58 | 0.000 |  |  |
| White | Reference | Reference | Reference | Reference |  |  |
| Mixed | 1.49 | 1.40 | 1.59 | 0.000 |  |  |
| Asian | 1.43 | 1.39 | 1.47 | 0.000 |  |  |
| Black | 2.11 | 2.02 | 2.21 | 0.000 |  |  |
| **Model 5^** |  |  |  |  |  |  |
| Probable non-migrant not in vulnerable situations (English as first language without complex social factors) | Reference | Reference | Reference | Reference | 405,365 | 0.067 |
| Probable migrants in vulnerable situations (English not their first language and complex social factors) | 2.20 | 2.10 | 2.30 | 0.000 |  |  |
| Probable migrants not in vulnerable situations (only English not their first language) | 1.22 | 1.19 | 1.25 | 0.000 |  |  |
| Probable non-migrants in vulnerable situations (only complex social factors) | 1.25 | 1.20 | 1.30 | 0.000 |  |  |
| Missing migration status (first language or complex social factor data missing) | 1.09 | 1.07 | 1.11 | 0.000 |  |  |
| Not most deprived | Reference | Reference | Reference | Reference |  |  |
| Most deprived | 1.69 | 1.64 | 1.73 | 0.000 |  |  |
| Mother's age at booking (years) | 0.92 | 0.92 | 0.92 | 0.000 |  |  |
| Number of previous live births | 1.54 | 1.53 | 1.55 | 0.000 |  |  |
| White | Reference | Reference | Reference | Reference |  |  |
| Mixed | 1.35 | 1.27 | 1.43 | 0.000 |  |  |
| Asian | 1.44 | 1.40 | 1.48 | 0.000 |  |  |
| Black | 1.95 | 1.87 | 2.04 | 0.000 |  |  |
| Other | 1.39 | 1.33 | 1.45 | 0.000 |  |  |
| Missing ethnicity | 1.18 | 1.15 | 1.20 | 0.000 |  |  |

| **Outcome: smoker at conception** | | | | | | |
| --- | --- | --- | --- | --- | --- | --- |
| **Migration status** | **Odds ratio** | **95% LCI** | **95% UCI** | **P value** | **Number of observations** | **Pseudo R squared (Hosmer-Lemeshow)** |
| **Model 1** | | | | | | |
| Probable non-migrant not in vulnerable situations (English as first language without complex social factors) | Reference | Reference | Reference | Reference | 604,514 | 0.018 |
| Probable migrants in vulnerable situations (English not their first language and complex social factors) | 0.89 | 0.86 | 0.92 | 0.000 |  |  |
| Probable migrants not in vulnerable situations (only English not their first language) | 0.73 | 0.71 | 0.75 | 0.000 |  |  |
| Probable non-migrants in vulnerable situations (only complex social factors) | 3.12 | 3.05 | 3.20 | 0.000 |  |  |
| Missing migration status (first language or complex social factor data missing) | 1.11 | 1.10 | 1.13 | 0.000 |  |  |
| **Model 2^** | | | | | | |
| Probable non-migrant not in vulnerable situations (English as first language without complex social factors) | Reference | Reference | Reference | Reference | 481,999 | 0.11 |
| Probable migrants in vulnerable situations (English not their first language and complex social factors) | 0.86 | 0.82 | 0.90 | 0.000 |  |  |
| Probable migrants not in vulnerable situations (only English not their first language) | 1.03 | 1.00 | 1.06 | 0.037 |  |  |
| Probable non-migrants in vulnerable situations (only complex social factors) | 1.80 | 1.74 | 1.87 | 0.000 |  |  |
| Missing migration status (first language or complex social factor data missing) | 1.05 | 1.03 | 1.07 | 0.000 |  |  |
| Not most deprived | Reference | Reference | Reference | Reference |  |  |
| Most deprived | 1.84 | 1.80 | 1.87 | 0.000 |  |  |
| Mother's age at booking (years) | 0.90 | 0.90 | 0.90 | 0.000 |  |  |
| Number of previous live births | 1.37 | 1.36 | 1.38 | 0.000 |  |  |
| White | Reference | Reference | Reference | Reference |  |  |
| Mixed | 0.80 | 0.76 | 0.85 | 0.000 |  |  |
| Asian | 0.12 | 0.11 | 0.13 | 0.000 |  |  |
| Black | 0.23 | 0.21 | 0.24 | 0.000 |  |  |
| Other | 0.43 | 0.41 | 0.46 | 0.000 |  |  |
| Missing ethnicity | 0.64 | 0.62 | 0.65 | 0.000 |  |  |
| **Model 3^** | | | | | | |
| Probable non-migrant not in vulnerable situations (English as first language without complex social factors) | Reference | Reference | Reference | Reference | 481,999 | 0.073 |
| Probable migrants in vulnerable situations (English not their first language and complex social factors) | 0.57 | 0.55 | 0.59 | 0.000 |  |  |
| Probable migrants not in vulnerable situations (only English not their first language) | 0.73 | 0.71 | 0.75 | 0.000 |  |  |
| Probable non-migrants in vulnerable situations (only complex social factors) | 1.76 | 1.70 | 1.82 | 0.000 |  |  |
| Missing migration status (first language or complex social factor data missing) | 1.01 | 0.99 | 1.03 | 0.423 |  |  |
| Not most deprived | Reference | Reference | Reference | Reference |  |  |
| Most deprived | 1.61 | 1.58 | 1.64 | 0.000 |  |  |
| Mother's age at booking (years) | 0.89 | 0.89 | 0.89 | 0.000 |  |  |
| Number of previous live births | 1.37 | 1.36 | 1.38 | 0.000 |  |  |
| **Model 4^** | | | | | | |
| Probable non-migrant not in vulnerable situations (English as first language without complex social factors) | Reference | Reference | Reference | Reference | 317,047 | 0.122 |
| Probable migrants in vulnerable situations (English not their first language and complex social factors) | 0.90 | 0.86 | 0.95 | 0.000 |  |  |
| Probable migrants not in vulnerable situations (only English not their first language) | 1.05 | 1.02 | 1.09 | 0.001 |  |  |
| Probable non-migrants in vulnerable situations (only complex social factors) | 1.80 | 1.73 | 1.87 | 0.000 |  |  |
| Not most deprived | Reference | Reference | Reference | Reference |  |  |
| Most deprived | 1.96 | 1.91 | 2.01 | 0.000 |  |  |
| Mother's age at booking (years) | 0.90 | 0.89 | 0.90 | 0.000 |  |  |
| Number of previous live births | 1.37 | 1.35 | 1.38 | 0.000 |  |  |
| White | Reference | Reference | Reference | Reference |  |  |
| Mixed | 0.80 | 0.75 | 0.86 | 0.000 |  |  |
| Asian | 0.11 | 0.10 | 0.12 | 0.000 |  |  |
| Black | 0.22 | 0.21 | 0.23 | 0.000 |  |  |
| Other | 0.43 | 0.41 | 0.46 | 0.000 |  |  |
| **Model 5^** | | | | | | |
| Probable non-migrant not in vulnerable situations (English as first language without complex social factors) | Reference | Reference | Reference | Reference | 481,999 | 0.11 |
| Probable migrants in vulnerable situations (English not their first language and complex social factors) | 0.86 | 0.82 | 0.90 | 0.000 |  |  |
| Probable migrants not in vulnerable situations (only English not their first language) | 1.03 | 1.00 | 1.06 | 0.037 |  |  |
| Probable non-migrants in vulnerable situations (only complex social factors) | 1.80 | 1.74 | 1.87 | 0.000 |  |  |
| Missing migration status (first language or complex social factor data missing) | 1.05 | 1.03 | 1.07 | 0.000 |  |  |
| Not most deprived | Reference | Reference | Reference | Reference |  |  |
| Most deprived | 1.84 | 1.80 | 1.87 | 0.000 |  |  |
| Mother's age at booking (years) | 0.90 | 0.90 | 0.90 | 0.000 |  |  |
| Number of previous live births | 1.37 | 1.36 | 1.38 | 0.000 |  |  |
| White | Reference | Reference | Reference | Reference |  |  |
| Mixed | 0.80 | 0.76 | 0.85 | 0.000 |  |  |
| Asian | 0.12 | 0.11 | 0.13 | 0.000 |  |  |
| Black | 0.23 | 0.21 | 0.24 | 0.000 |  |  |
| Other | 0.43 | 0.41 | 0.46 | 0.000 |  |  |
| Missing ethnicity | 0.64 | 0.62 | 0.65 | 0.000 |  |  |

| **Outcome: smokers who did not quit smoking during year before pregnancy** | | | | | | |
| --- | --- | --- | --- | --- | --- | --- |
| **Migration status** | **Odds ratio** | **95% LCI** | **95% UCI** | **P value** | **Number of observations** | **Pseudo R squared (Hosmer-Lemeshow)** |
| **Model 1** | | | | | | |
| Probable non-migrant not in vulnerable situations (English as first language without complex social factors) | Reference | Reference | Reference | Reference | 138,422 | 0.009 |
| Probable migrants in vulnerable situations (English not their first language and complex social factors) | 1.63 | 1.49 | 1.79 | 0.000 |  |  |
| Probable migrants not in vulnerable situations (only English not their first language) | 1.00 | 0.95 | 1.05 | 0.994 |  |  |
| Probable non-migrants in vulnerable situations (only complex social factors) | 1.88 | 1.78 | 1.99 | 0.000 |  |  |
| Missing migration status (first language or complex social factor data missing) | 1.56 | 1.50 | 1.61 | 0.000 |  |  |
| **Model 2^** | | | | | | |
| Probable non-migrant not in vulnerable situations (English as first language without complex social factors) | Reference | Reference | Reference | Reference | 104,675 | 0.047 |
| Probable migrants in vulnerable situations (English not their first language and complex social factors) | 1.56 | 1.41 | 1.73 | 0.000 |  |  |
| Probable migrants not in vulnerable situations (only English not their first language) | 1.21 | 1.15 | 1.28 | 0.000 |  |  |
| Probable non-migrants in vulnerable situations (only complex social factors) | 1.33 | 1.23 | 1.44 | 0.000 |  |  |
| Missing migration status (first language or complex social factor data missing) | 1.32 | 1.27 | 1.38 | 0.000 |  |  |
| Not most deprived | Reference | Reference | Reference | Reference |  |  |
| Most deprived | 1.96 | 1.86 | 2.07 | 0.000 |  |  |
| Mother's age at booking (years) | 0.95 | 0.94 | 0.95 | 0.000 |  |  |
| Number of previous live births | 1.45 | 1.42 | 1.47 | 0.000 |  |  |
| White | Reference | Reference | Reference | Reference |  |  |
| Mixed | 0.82 | 0.73 | 0.92 | 0.001 |  |  |
| Asian | 0.78 | 0.70 | 0.86 | 0.000 |  |  |
| Black | 0.66 | 0.59 | 0.74 | 0.000 |  |  |
| Other | 0.85 | 0.76 | 0.96 | 0.006 |  |  |
| Missing ethnicity | 0.80 | 0.77 | 0.85 | 0.000 |  |  |
| **Model 3^** | | | | | | |
| Probable non-migrant not in vulnerable situations (English as first language without complex social factors) | Reference | Reference | Reference | Reference | 104,675 | 0.046 |
| Probable migrants in vulnerable situations (English not their first language and complex social factors) | 1.52 | 1.37 | 1.68 | 0.000 |  |  |
| Probable migrants not in vulnerable situations (only English not their first language) | 1.19 | 1.12 | 1.26 | 0.000 |  |  |
| Probable non-migrants in vulnerable situations (only complex social factors) | 1.33 | 1.23 | 1.44 | 0.000 |  |  |
| Missing migration status (first language or complex social factor data missing) | 1.31 | 1.26 | 1.37 | 0.000 |  |  |
| Not most deprived | Reference | Reference | Reference | Reference |  |  |
| Most deprived | 1.94 | 1.84 | 2.05 | 0.000 |  |  |
| Mother's age at booking (years) | 0.94 | 0.94 | 0.95 | 0.000 |  |  |
| Number of previous live births | 1.46 | 1.43 | 1.48 | 0.000 |  |  |
| **Model 4^** | | | | | | |
| Probable non-migrant not in vulnerable situations (English as first language without complex social factors) | Reference | Reference | Reference | Reference | 70,106 | 0.049 |
| Probable migrants in vulnerable situations (English not their first language and complex social factors) | 1.51 | 1.34 | 1.70 | 0.000 |  |  |
| Probable migrants not in vulnerable situations (only English not their first language) | 1.23 | 1.15 | 1.31 | 0.000 |  |  |
| Probable non-migrants in vulnerable situations (only complex social factors) | 1.30 | 1.20 | 1.41 | 0.000 |  |  |
| Not most deprived | Reference | Reference | Reference | Reference |  |  |
| Most deprived | 2.06 | 1.93 | 2.20 | 0.000 |  |  |
| Mother's age at booking (years) | 0.94 | 0.94 | 0.95 | 0.000 |  |  |
| Number of previous live births | 1.45 | 1.42 | 1.48 | 0.000 |  |  |
| White | Reference | Reference | Reference | Reference |  |  |
| Mixed | 0.81 | 0.71 | 0.92 | 0.001 |  |  |
| Asian | 0.72 | 0.65 | 0.81 | 0.000 |  |  |
| Black | 0.61 | 0.54 | 0.69 | 0.000 |  |  |
| Other | 0.86 | 0.76 | 0.98 | 0.019 |  |  |
| **Model 5^** | | | | | | |
| Probable non-migrant not in vulnerable situations (English as first language without complex social factors) | Reference | Reference | Reference | Reference | 104,675 | 0.047 |
| Probable migrants in vulnerable situations (English not their first language and complex social factors) | 1.56 | 1.41 | 1.73 | 0.000 |  |  |
| Probable migrants not in vulnerable situations (only English not their first language) | 1.21 | 1.15 | 1.28 | 0.000 |  |  |
| Probable non-migrants in vulnerable situations (only complex social factors) | 1.33 | 1.23 | 1.44 | 0.000 |  |  |
| Missing migration status (first language or complex social factor data missing) | 1.32 | 1.27 | 1.38 | 0.000 |  |  |
| Not most deprived | Reference | Reference | Reference | Reference |  |  |
| Most deprived | 1.96 | 1.86 | 2.07 | 0.000 |  |  |
| Mother's age at booking (years) | 0.95 | 0.94 | 0.95 | 0.000 |  |  |
| Number of previous live births | 1.45 | 1.42 | 1.47 | 0.000 |  |  |
| White | Reference | Reference | Reference | Reference |  |  |
| Mixed | 0.82 | 0.73 | 0.92 | 0.001 |  |  |
| Asian | 0.78 | 0.70 | 0.86 | 0.000 |  |  |
| Black | 0.66 | 0.59 | 0.74 | 0.000 |  |  |
| Other | 0.85 | 0.76 | 0.96 | 0.006 |  |  |
| Missing ethnicity | 0.80 | 0.77 | 0.85 | 0.000 |  |  |

| **Outcome: underweight** | | | | | | |
| --- | --- | --- | --- | --- | --- | --- |
| **Migration status** | **Odds ratio** | **95% LCI** | **95% UCI** | **P value** | **Number of observations** | **Pseudo R squared (Hosmer-Lemeshow)** |
| **Model 1** | | | | | | |
| Probable non-migrant not in vulnerable situations (English as first language without complex social factors) | Reference | Reference | Reference | Reference | 496,267 | 0.009 |
| Probable migrants in vulnerable situations (English not their first language and complex social factors) | 2.07 | 1.92 | 2.22 | 0.000 |  |  |
| Probable migrants not in vulnerable situations (only English not their first language) | 1.35 | 1.28 | 1.42 | 0.000 |  |  |
| Probable non-migrants in vulnerable situations (only complex social factors) | 2.58 | 2.44 | 2.71 | 0.000 |  |  |
| Missing migration status (first language or complex social factor data missing) | 1.11 | 1.07 | 1.16 | 0.000 |  |  |
| **Model 2^** | | | | | | |
| Probable non-migrant not in vulnerable situations (English as first language without complex social factors) | Reference | Reference | Reference | Reference | 421,658 | 0.031 |
| Probable migrants in vulnerable situations (English not their first language and complex social factors) | 1.55 | 1.44 | 1.68 | 0.000 |  |  |
| Probable migrants not in vulnerable situations (only English not their first language) | 1.26 | 1.19 | 1.33 | 0.000 |  |  |
| Probable non-migrants in vulnerable situations (only complex social factors) | 1.60 | 1.51 | 1.70 | 0.000 |  |  |
| Missing migration status (first language or complex social factor data missing) | 1.07 | 1.03 | 1.12 | 0.002 |  |  |
| Not most deprived | Reference | Reference | Reference | Reference |  |  |
| Most deprived | 1.08 | 1.03 | 1.13 | 0.002 |  |  |
| Mother's age at booking (years) | 0.93 | 0.92 | 0.93 | 0.000 |  |  |
| Number of previous live births | 0.97 | 0.95 | 0.99 | 0.002 |  |  |
| White | Reference | Reference | Reference | Reference |  |  |
| Mixed | 1.04 | 0.91 | 1.18 | 0.596 |  |  |
| Asian | 1.55 | 1.47 | 1.63 | 0.000 |  |  |
| Black | 0.72 | 0.64 | 0.80 | 0.000 |  |  |
| Other | 1.09 | 0.98 | 1.21 | 0.113 |  |  |
| Missing ethnicity | 1.14 | 1.08 | 1.20 | 0.000 |  |  |
| **Model 3^** | | | | | | |
| Probable non-migrant not in vulnerable situations (English as first language without complex social factors) | Reference | Reference | Reference | Reference | 421,658 | 0.028 |
| Probable migrants in vulnerable situations (English not their first language and complex social factors) | 1.70 | 1.58 | 1.84 | 0.000 |  |  |
| Probable migrants not in vulnerable situations (only English not their first language) | 1.38 | 1.30 | 1.45 | 0.000 |  |  |
| Probable non-migrants in vulnerable situations (only complex social factors) | 1.58 | 1.49 | 1.68 | 0.000 |  |  |
| Missing migration status (first language or complex social factor data missing) | 1.08 | 1.04 | 1.13 | 0.001 |  |  |
| Not most deprived | Reference | Reference | Reference | Reference |  |  |
| Most deprived | 1.09 | 1.04 | 1.14 | 0.001 |  |  |
| Mother's age at booking (years) | 0.93 | 0.93 | 0.93 | 0.000 |  |  |
| Number of previous live births | 0.96 | 0.95 | 0.98 | 0.000 |  |  |
| **Model 4^** | | | | | | |
| Probable non-migrant not in vulnerable situations (English as first language without complex social factors) | Reference | Reference | Reference | Reference | 286,331 | 0.033 |
| Probable migrants in vulnerable situations (English not their first language and complex social factors) | 1.54 | 1.41 | 1.68 | 0.000 |  |  |
| Probable migrants not in vulnerable situations (only English not their first language) | 1.22 | 1.14 | 1.30 | 0.000 |  |  |
| Probable non-migrants in vulnerable situations (only complex social factors) | 1.61 | 1.50 | 1.72 | 0.000 |  |  |
| Not most deprived | Reference | Reference | Reference | Reference |  |  |
| Most deprived | 1.06 | 1.00 | 1.12 | 0.065 |  |  |
| Mother's age at booking (years) | 0.93 | 0.92 | 0.93 | 0.000 |  |  |
| Number of previous live births | 0.98 | 0.95 | 1.00 | 0.037 |  |  |
| White | Reference | Reference | Reference | Reference |  |  |
| Mixed | 1.03 | 0.89 | 1.19 | 0.667 |  |  |
| Asian | 1.45 | 1.36 | 1.54 | 0.000 |  |  |
| Black | 0.68 | 0.60 | 0.78 | 0.000 |  |  |
| Other | 1.05 | 0.94 | 1.18 | 0.388 |  |  |
| **Model 5^** | | | | | | |
| Probable non-migrant not in vulnerable situations (English as first language without complex social factors) | Reference | Reference | Reference | Reference | 421,658 | 0.031 |
| Probable migrants in vulnerable situations (English not their first language and complex social factors) | 1.55 | 1.44 | 1.68 | 0.000 |  |  |
| Probable migrants not in vulnerable situations (only English not their first language) | 1.26 | 1.19 | 1.33 | 0.000 |  |  |
| Probable non-migrants in vulnerable situations (only complex social factors) | 1.60 | 1.51 | 1.70 | 0.000 |  |  |
| Missing migration status (first language or complex social factor data missing) | 1.07 | 1.03 | 1.12 | 0.002 |  |  |
| Not most deprived | Reference | Reference | Reference | Reference |  |  |
| Most deprived | 1.08 | 1.03 | 1.13 | 0.002 |  |  |
| Mother's age at booking (years) | 0.93 | 0.92 | 0.93 | 0.000 |  |  |
| Number of previous live births | 0.97 | 0.95 | 0.99 | 0.002 |  |  |
| White | Reference | Reference | Reference | Reference |  |  |
| Mixed | 1.04 | 0.91 | 1.18 | 0.596 |  |  |
| Asian | 1.55 | 1.47 | 1.63 | 0.000 |  |  |
| Black | 0.72 | 0.64 | 0.80 | 0.000 |  |  |
| Other | 1.09 | 0.98 | 1.21 | 0.113 |  |  |
| Missing ethnicity | 1.14 | 1.08 | 1.20 | 0.000 |  |  |

| **Outcome: overweight or obesity** | | | | | | |
| --- | --- | --- | --- | --- | --- | --- |
| **Migration status** | **Odds ratio** | **95% LCI** | **95% UCI** | **P value** | **Number of observations** | **Pseudo R squared (Hosmer-Lemeshow)** |
| **Model 1** | | | | | | |
| Probable non-migrant not in vulnerable situations (English as first language without complex social factors) | Reference | Reference | Reference | Reference | 496,267 | 0.001 |
| Probable migrants in vulnerable situations (English not their first language and complex social factors) | 0.79 | 0.77 | 0.82 | 0.000 |  |  |
| Probable migrants not in vulnerable situations (only English not their first language) | 0.81 | 0.79 | 0.82 | 0.000 |  |  |
| Probable non-migrants in vulnerable situations (only complex social factors) | 0.90 | 0.88 | 0.93 | 0.000 |  |  |
| Missing migration status (first language or complex social factor data missing) | 1.03 | 1.01 | 1.04 | 0.000 |  |  |
| **Model 2^** | | | | | | |
| Probable non-migrant not in vulnerable situations (English as first language without complex social factors) | Reference | Reference | Reference | Reference | 421,658 | 0.031 |
| Probable migrants in vulnerable situations (English not their first language and complex social factors) | 0.73 | 0.71 | 0.75 | 0.000 |  |  |
| Probable migrants not in vulnerable situations (only English not their first language) | 0.77 | 0.76 | 0.79 | 0.000 |  |  |
| Probable non-migrants in vulnerable situations (only complex social factors) | 0.89 | 0.87 | 0.92 | 0.000 |  |  |
| Missing migration status (first language or complex social factor data missing) | 1.01 | 1.00 | 1.03 | 0.076 |  |  |
| Not most deprived | Reference | Reference | Reference | Reference |  |  |
| Most deprived | 1.30 | 1.28 | 1.32 | 0.000 |  |  |
| Mother's age at booking (years) | 1.00 | 1.00 | 1.00 | 0.049 |  |  |
| Number of previous live births | 1.23 | 1.23 | 1.24 | 0.000 |  |  |
| White | Reference | Reference | Reference | Reference |  |  |
| Mixed | 1.10 | 1.06 | 1.16 | 0.000 |  |  |
| Asian | 1.04 | 1.02 | 1.06 | 0.000 |  |  |
| Black | 1.97 | 1.91 | 2.04 | 0.000 |  |  |
| Other | 0.96 | 0.92 | 0.99 | 0.021 |  |  |
| Missing ethnicity | 0.89 | 0.87 | 0.91 | 0.000 |  |  |
| **Model 3^** | | | | | | |
| Probable non-migrant not in vulnerable situations (English as first language without complex social factors) | Reference | Reference | Reference | Reference | 421,658 | 0.024 |
| Probable migrants in vulnerable situations (English not their first language and complex social factors) | 0.74 | 0.72 | 0.77 | 0.000 |  |  |
| Probable migrants not in vulnerable situations (only English not their first language) | 0.78 | 0.77 | 0.80 | 0.000 |  |  |
| Probable non-migrants in vulnerable situations (only complex social factors) | 0.90 | 0.87 | 0.92 | 0.000 |  |  |
| Missing migration status (first language or complex social factor data missing) | 1.01 | 1.00 | 1.03 | 0.068 |  |  |
| Not most deprived | Reference | Reference | Reference | Reference |  |  |
| Most deprived | 1.33 | 1.31 | 1.36 | 0.000 |  |  |
| Mother's age at booking (years) | 1.00 | 1.00 | 1.00 | 0.750 |  |  |
| Number of previous live births | 1.24 | 1.24 | 1.25 | 0.000 |  |  |
| **Model 4^** | | | | | | |
| Probable non-migrant not in vulnerable situations (English as first language without complex social factors) | Reference | Reference | Reference | Reference | 286,331 | 0.017 |
| Probable migrants in vulnerable situations (English not their first language and complex social factors) | 0.71 | 0.69 | 0.74 | 0.000 |  |  |
| Probable migrants not in vulnerable situations (only English not their first language) | 0.78 | 0.76 | 0.80 | 0.000 |  |  |
| Probable non-migrants in vulnerable situations (only complex social factors) | 0.89 | 0.86 | 0.92 | 0.000 |  |  |
| Not most deprived | Reference | Reference | Reference | Reference |  |  |
| Most deprived | 1.31 | 1.28 | 1.34 | 0.000 |  |  |
| Mother's age at booking (years) | 1.00 | 1.00 | 1.00 | 0.001 |  |  |
| Number of previous live births | 1.23 | 1.23 | 1.24 | 0.000 |  |  |
| White | Reference | Reference | Reference | Reference |  |  |
| Mixed | 1.11 | 1.05 | 1.17 | 0.000 |  |  |
| Asian | 1.09 | 1.06 | 1.11 | 0.000 |  |  |
| Black | 2.03 | 1.95 | 2.11 | 0.000 |  |  |
| Other | 0.97 | 0.93 | 1.01 | 0.174 |  |  |
| **Model 5^** | | | | | | |
| Probable non-migrant not in vulnerable situations (English as first language without complex social factors) | Reference | Reference | Reference | Reference | 421,658 | 0.017 |
| Probable migrants in vulnerable situations (English not their first language and complex social factors) | 0.73 | 0.71 | 0.75 | 0.000 |  |  |
| Probable migrants not in vulnerable situations (only English not their first language) | 0.77 | 0.76 | 0.79 | 0.000 |  |  |
| Probable non-migrants in vulnerable situations (only complex social factors) | 0.89 | 0.87 | 0.92 | 0.000 |  |  |
| Missing migration status (first language or complex social factor data missing) | 1.01 | 1.00 | 1.03 | 0.076 |  |  |
| Not most deprived | Reference | Reference | Reference | Reference |  |  |
| Most deprived | 1.30 | 1.28 | 1.32 | 0.000 |  |  |
| Mother's age at booking (years) | 1.00 | 1.00 | 1.00 | 0.049 |  |  |
| Number of previous live births | 1.23 | 1.23 | 1.24 | 0.000 |  |  |
| White | Reference | Reference | Reference | Reference |  |  |
| Mixed | 1.10 | 1.06 | 1.16 | 0.000 |  |  |
| Asian | 1.04 | 1.02 | 1.06 | 0.000 |  |  |
| Black | 1.98 | 1.91 | 2.04 | 0.000 |  |  |
| Other | 0.96 | 0.92 | 0.99 | 0.021 |  |  |
| Missing ethnicity | 0.89 | 0.87 | 0.91 | 0.000 |  |  |

| **Outcome: at least one mental or physical condition** | | | | | | |
| --- | --- | --- | --- | --- | --- | --- |
| **Migration status** | **Odds ratio** | **95% LCI** | **95% UCI** | **P value** | **Number of observations** | **Pseudo R squared (Hosmer-Lemeshow)** |
| **Model 1** | | | | | | |
| Probable non-migrant not in vulnerable situations (English as first language without complex social factors) | Reference | Reference | Reference | Reference | 652,880 | 0.049 |
| Probable migrants in vulnerable situations (English not their first language and complex social factors) | 0.44 | 0.43 | 0.45 | 0.000 |  |  |
| Probable migrants not in vulnerable situations (only English not their first language) | 0.72 | 0.71 | 0.74 | 0.000 |  |  |
| Probable non-migrants in vulnerable situations (only complex social factors) | 0.98 | 0.96 | 1.00 | 0.075 |  |  |
| Missing migration status (first language or complex social factor data missing) | 0.27 | 0.27 | 0.28 | 0.000 |  |  |
| **Model 2** | | | | | | |
| Probable non-migrant not in vulnerable situations (English as first language without complex social factors) | Reference | Reference | Reference | Reference | 652,871 | 0.059 |
| Probable migrants in vulnerable situations (English not their first language and complex social factors) | 0.54 | 0.52 | 0.56 | 0.000 |  |  |
| Probable migrants not in vulnerable situations (only English not their first language) | 0.83 | 0.82 | 0.85 | 0.000 |  |  |
| Probable non-migrants in vulnerable situations (only complex social factors) | 1.06 | 1.03 | 1.08 | 0.000 |  |  |
| Missing migration status (first language or complex social factor data missing) | 0.28 | 0.28 | 0.28 | 0.000 |  |  |
| Not most deprived | Reference | Reference | Reference | Reference |  |  |
| Most deprived | 1.24 | 1.22 | 1.26 | 0.000 |  |  |
| Mother's age at booking (years) | 1.02 | 1.02 | 1.02 | 0.000 |  |  |
| White | Reference | Reference | Reference | Reference |  |  |
| Mixed | 0.88 | 0.85 | 0.92 | 0.000 |  |  |
| Asian | 0.53 | 0.52 | 0.54 | 0.000 |  |  |
| Black | 0.69 | 0.66 | 0.71 | 0.000 |  |  |
| Other | 0.52 | 0.50 | 0.54 | 0.000 |  |  |
| Missing ethnicity | 0.64 | 0.63 | 0.65 | 0.000 |  |  |
| **Model 3** | | | | | | |
| Probable non-migrant not in vulnerable situations (English as first language without complex social factors) | Reference | Reference | Reference | Reference | 652,871 | 0.051 |
| Probable migrants in vulnerable situations (English not their first language and complex social factors) | 0.44 | 0.43 | 0.46 | 0.000 |  |  |
| Probable migrants not in vulnerable situations (only English not their first language) | 0.71 | 0.69 | 0.72 | 0.000 |  |  |
| Probable non-migrants in vulnerable situations (only complex social factors) | 1.07 | 1.04 | 1.09 | 0.000 |  |  |
| Missing migration status (first language or complex social factor data missing) | 0.27 | 0.27 | 0.28 | 0.000 |  |  |
| Not most deprived | Reference | Reference | Reference | Reference |  |  |
| Most deprived | 1.19 | 1.17 | 1.21 | 0.000 |  |  |
| Mother's age at booking (years) | 1.02 | 1.02 | 1.02 | 0.000 |  |  |
| **Model 4** | | | | | | |
| Probable non-migrant not in vulnerable situations (English as first language without complex social factors) | Reference | Reference | Reference | Reference | 373,367 | 0.016 |
| Probable migrants in vulnerable situations (English not their first language and complex social factors) | 0.54 | 0.52 | 0.56 | 0.000 |  |  |
| Probable migrants not in vulnerable situations (only English not their first language) | 0.85 | 0.83 | 0.86 | 0.000 |  |  |
| Probable non-migrants in vulnerable situations (only complex social factors) | 1.07 | 1.04 | 1.09 | 0.000 |  |  |
| Not most deprived | Reference | Reference | Reference | Reference |  |  |
| Most deprived | 1.40 | 1.37 | 1.43 | 0.000 |  |  |
| Mother's age at booking (years) | 1.02 | 1.02 | 1.02 | 0.000 |  |  |
| White | Reference | Reference | Reference | Reference |  |  |
| Mixed | 0.87 | 0.83 | 0.91 | 0.000 |  |  |
| Asian | 0.55 | 0.54 | 0.57 | 0.000 |  |  |
| Black | 0.72 | 0.70 | 0.75 | 0.000 |  |  |
| Other | 0.53 | 0.51 | 0.55 | 0.000 |  |  |
| **Model 5** | | | | | | |
| Probable non-migrant not in vulnerable situations (English as first language without complex social factors) | Reference | Reference | Reference | Reference | 622,544 | 0.058 |
| Probable migrants in vulnerable situations (English not their first language and complex social factors) | 0.54 | 0.52 | 0.56 | 0.000 |  |  |
| Probable migrants not in vulnerable situations (only English not their first language) | 0.83 | 0.82 | 0.85 | 0.000 |  |  |
| Probable non-migrants in vulnerable situations (only complex social factors) | 1.05 | 1.02 | 1.08 | 0.003 |  |  |
| Missing migration status (first language or complex social factor data missing) | 0.29 | 0.28 | 0.29 | 0.000 |  |  |
| Not most deprived | Reference | Reference | Reference | Reference |  |  |
| Most deprived | 1.26 | 1.24 | 1.28 | 0.000 |  |  |
| Mother's age at booking (years) | 1.02 | 1.02 | 1.02 | 0.000 |  |  |
| White | Reference | Reference | Reference | Reference |  |  |
| Mixed | 0.88 | 0.84 | 0.92 | 0.000 |  |  |
| Asian | 0.53 | 0.52 | 0.55 | 0.000 |  |  |
| Black | 0.69 | 0.66 | 0.71 | 0.000 |  |  |
| Other | 0.53 | 0.51 | 0.55 | 0.000 |  |  |
| Missing ethnicity | 0.64 | 0.63 | 0.65 | 0.000 |  |  |

| **Outcome: at least one mental health condition** | | | | | | |
| --- | --- | --- | --- | --- | --- | --- |
| **Migration status** | **Odds ratio** | **95% LCI** | **95% UCI** | **P value** | **Number of observations** | **Pseudo R squared (Hosmer-Lemeshow)** |
| **Model 1** | | | | | | |
| Probable non-migrant not in vulnerable situations (English as first language without complex social factors) | Reference | Reference | Reference | Reference | 652,880 | 0.042 |
| Probable migrants in vulnerable situations (English not their first language and complex social factors) | 0.31 | 0.29 | 0.33 | 0.000 |  |  |
| Probable migrants not in vulnerable situations (only English not their first language) | 0.42 | 0.41 | 0.44 | 0.000 |  |  |
| Probable non-migrants in vulnerable situations (only complex social factors) | 1.69 | 1.65 | 1.74 | 0.000 |  |  |
| Missing migration status (first language or complex social factor data missing) | 0.32 | 0.31 | 0.33 | 0.000 |  |  |
| **Model 2** | | | | | | |
| Probable non-migrant not in vulnerable situations (English as first language without complex social factors) | Reference | Reference | Reference | Reference | 652,871 | 0.06 |
| Probable migrants in vulnerable situations (English not their first language and complex social factors) | 0.36 | 0.34 | 0.39 | 0.000 |  |  |
| Probable migrants not in vulnerable situations (only English not their first language) | 0.53 | 0.51 | 0.54 | 0.000 |  |  |
| Probable non-migrants in vulnerable situations (only complex social factors) | 1.41 | 1.37 | 1.46 | 0.000 |  |  |
| Missing migration status (first language or complex social factor data missing) | 0.32 | 0.31 | 0.33 | 0.000 |  |  |
| Not most deprived | Reference | Reference | Reference | Reference |  |  |
| Most deprived | 1.44 | 1.40 | 1.47 | 0.000 |  |  |
| Mother's age at booking (years) | 0.98 | 0.98 | 0.98 | 0.000 |  |  |
| White | Reference | Reference | Reference | Reference |  |  |
| Mixed | 0.90 | 0.85 | 0.96 | 0.001 |  |  |
| Asian | 0.31 | 0.29 | 0.32 | 0.000 |  |  |
| Black | 0.42 | 0.40 | 0.45 | 0.000 |  |  |
| Other | 0.35 | 0.33 | 0.38 | 0.000 |  |  |
| Missing ethnicity | 0.65 | 0.63 | 0.67 | 0.000 |  |  |
| **Model 3** | | | | | | |
| Probable non-migrant not in vulnerable situations (English as first language without complex social factors) | Reference | Reference | Reference | Reference | 652,871 | 0.046 |
| Probable migrants in vulnerable situations (English not their first language and complex social factors) | 0.27 | 0.26 | 0.29 | 0.000 |  |  |
| Probable migrants not in vulnerable situations (only English not their first language) | 0.42 | 0.40 | 0.43 | 0.000 |  |  |
| Probable non-migrants in vulnerable situations (only complex social factors) | 1.42 | 1.38 | 1.46 | 0.000 |  |  |
| Missing migration status (first language or complex social factor data missing) | 0.31 | 0.30 | 0.32 | 0.000 |  |  |
| Not most deprived | Reference | Reference | Reference | Reference |  |  |
| Most deprived | 1.35 | 1.32 | 1.39 | 0.000 |  |  |
| Mother's age at booking (years) | 0.98 | 0.98 | 0.98 | 0.000 |  |  |
| **Model 4** | | | | | | |
| Probable non-migrant not in vulnerable situations (English as first language without complex social factors) | Reference | Reference | Reference | Reference | 373,367 | 0.041 |
| Probable migrants in vulnerable situations (English not their first language and complex social factors) | 0.38 | 0.35 | 0.40 | 0.000 |  |  |
| Probable migrants not in vulnerable situations (only English not their first language) | 0.55 | 0.52 | 0.57 | 0.000 |  |  |
| Probable non-migrants in vulnerable situations (only complex social factors) | 1.42 | 1.37 | 1.46 | 0.000 |  |  |
| Not most deprived | Reference | Reference | Reference | Reference |  |  |
| Most deprived | 1.60 | 1.56 | 1.65 | 0.000 |  |  |
| Mother's age at booking (years) | 0.98 | 0.98 | 0.98 | 0.000 |  |  |
| White | Reference | Reference | Reference | Reference |  |  |
| Mixed | 0.88 | 0.82 | 0.94 | 0.000 |  |  |
| Asian | 0.33 | 0.31 | 0.34 | 0.000 |  |  |
| Black | 0.45 | 0.42 | 0.48 | 0.000 |  |  |
| Other | 0.37 | 0.34 | 0.40 | 0.000 |  |  |
| **Model 5** | | | | | | |
| Probable non-migrant not in vulnerable situations (English as first language without complex social factors) | Reference | Reference | Reference | Reference | 622,544 | 0.057 |
| Probable migrants in vulnerable situations (English not their first language and complex social factors) | 0.33 | 0.31 | 0.35 | 0.000 |  |  |
| Probable migrants not in vulnerable situations (only English not their first language) | 0.53 | 0.51 | 0.55 | 0.000 |  |  |
| Probable non-migrants in vulnerable situations (only complex social factors) | 1.48 | 1.42 | 1.53 | 0.000 |  |  |
| Missing migration status (first language or complex social factor data missing) | 0.32 | 0.32 | 0.33 | 0.000 |  |  |
| Not most deprived | Reference | Reference | Reference | Reference |  |  |
| Most deprived | 1.48 | 1.44 | 1.51 | 0.000 |  |  |
| Mother's age at booking (years) | 0.98 | 0.98 | 0.98 | 0.000 |  |  |
| White | Reference | Reference | Reference | Reference |  |  |
| Mixed | 0.90 | 0.84 | 0.96 | 0.001 |  |  |
| Asian | 0.31 | 0.30 | 0.32 | 0.000 |  |  |
| Black | 0.42 | 0.39 | 0.44 | 0.000 |  |  |
| Other | 0.35 | 0.33 | 0.38 | 0.000 |  |  |
| Missing ethnicity | 0.65 | 0.63 | 0.67 | 0.000 |  |  |

| **Outcome: at least one physical health condition** | | | | | | |
| --- | --- | --- | --- | --- | --- | --- |
| **Migration status** | **Odds ratio** | **95% LCI** | **95% UCI** | **P value** | **Number of observations** | **Pseudo R squared (Hosmer-Lemeshow)** |
| **Model 1** | | | | | | |
| Probable non-migrant not in vulnerable situations (English as first language without complex social factors) | Reference | Reference | Reference | Reference | 652,880 | 0.042 |
| Probable migrants in vulnerable situations (English not their first language and complex social factors) | 0.50 | 0.48 | 0.52 | 0.000 |  |  |
| Probable migrants not in vulnerable situations (only English not their first language) | 0.85 | 0.83 | 0.86 | 0.000 |  |  |
| Probable non-migrants in vulnerable situations (only complex social factors) | 0.69 | 0.67 | 0.71 | 0.000 |  |  |
| Missing migration status (first language or complex social factor data missing) | 0.28 | 0.28 | 0.29 | 0.000 |  |  |
| **Model 2** | | | | | | |
| Probable non-migrant not in vulnerable situations (English as first language without complex social factors) | Reference | Reference | Reference | Reference | 652,871 | 0.053 |
| Probable migrants in vulnerable situations (English not their first language and complex social factors) | 0.62 | 0.60 | 0.64 | 0.000 |  |  |
| Probable migrants not in vulnerable situations (only English not their first language) | 0.94 | 0.92 | 0.96 | 0.000 |  |  |
| Probable non-migrants in vulnerable situations (only complex social factors) | 0.82 | 0.80 | 0.84 | 0.000 |  |  |
| Missing migration status (first language or complex social factor data missing) | 0.29 | 0.29 | 0.30 | 0.000 |  |  |
| Not most deprived | Reference | Reference | Reference | Reference |  |  |
| Most deprived | 1.16 | 1.14 | 1.18 | 0.000 |  |  |
| Mother's age at booking (years) | 1.04 | 1.04 | 1.04 | 0.000 |  |  |
| White | Reference | Reference | Reference | Reference |  |  |
| Mixed | 0.90 | 0.86 | 0.94 | 0.000 |  |  |
| Asian | 0.62 | 0.61 | 0.64 | 0.000 |  |  |
| Black | 0.81 | 0.78 | 0.84 | 0.000 |  |  |
| Other | 0.60 | 0.57 | 0.62 | 0.000 |  |  |
| Missing ethnicity | 0.66 | 0.65 | 0.67 | 0.000 |  |  |
| **Model 3** | | | | | | |
| Probable non-migrant not in vulnerable situations (English as first language without complex social factors) | Reference | Reference | Reference | Reference | 652,871 | 0.048 |
| Probable migrants in vulnerable situations (English not their first language and complex social factors) | 0.53 | 0.51 | 0.55 | 0.000 |  |  |
| Probable migrants not in vulnerable situations (only English not their first language) | 0.83 | 0.81 | 0.84 | 0.000 |  |  |
| Probable non-migrants in vulnerable situations (only complex social factors) | 0.83 | 0.80 | 0.85 | 0.000 |  |  |
| Missing migration status (first language or complex social factor data missing) | 0.28 | 0.28 | 0.29 | 0.000 |  |  |
| Not most deprived | Reference | Reference | Reference | Reference |  |  |
| Most deprived | 1.13 | 1.11 | 1.15 | 0.000 |  |  |
| Mother's age at booking (years) | 1.04 | 1.03 | 1.04 | 0.000 |  |  |
| **Model 4** | | | | | | |
| Probable non-migrant not in vulnerable situations (English as first language without complex social factors) | Reference | Reference | Reference | Reference | 355,517 | 0.013 |
| Probable migrants in vulnerable situations (English not their first language and complex social factors) | 0.61 | 0.58 | 0.64 | 0.000 |  |  |
| Probable migrants not in vulnerable situations (only English not their first language) | 0.95 | 0.93 | 0.97 | 0.000 |  |  |
| Probable non-migrants in vulnerable situations (only complex social factors) | 0.86 | 0.83 | 0.90 | 0.000 |  |  |
| Not most deprived | Reference | Reference | Reference | Reference |  |  |
| Most deprived | 1.28 | 1.26 | 1.31 | 0.000 |  |  |
| Mother's age at booking (years) | 1.04 | 1.04 | 1.04 | 0.000 |  |  |
| White | Reference | Reference | Reference | Reference |  |  |
| Mixed | 0.89 | 0.84 | 0.94 | 0.000 |  |  |
| Asian | 0.65 | 0.64 | 0.67 | 0.000 |  |  |
| Black | 0.85 | 0.82 | 0.88 | 0.000 |  |  |
| Other | 0.61 | 0.58 | 0.64 | 0.000 |  |  |
| **Model 5** | | | | | | |
| Probable non-migrant not in vulnerable situations (English as first language without complex social factors) | Reference | Reference | Reference | Reference | 622,544 | 0.051 |
| Probable migrants in vulnerable situations (English not their first language and complex social factors) | 0.62 | 0.60 | 0.64 | 0.000 |  |  |
| Probable migrants not in vulnerable situations (only English not their first language) | 0.94 | 0.92 | 0.96 | 0.000 |  |  |
| Probable non-migrants in vulnerable situations (only complex social factors) | 0.86 | 0.83 | 0.89 | 0.000 |  |  |
| Missing migration status (first language or complex social factor data missing) | 0.30 | 0.29 | 0.30 | 0.000 |  |  |
| Not most deprived | Reference | Reference | Reference | Reference |  |  |
| Most deprived | 1.17 | 1.14 | 1.19 | 0.000 |  |  |
| Mother's age at booking (years) | 1.04 | 1.03 | 1.04 | 0.000 |  |  |
| White | Reference | Reference | Reference | Reference |  |  |
| Mixed | 0.90 | 0.85 | 0.94 | 0.000 |  |  |
| Asian | 0.63 | 0.61 | 0.64 | 0.000 |  |  |
| Black | 0.81 | 0.78 | 0.83 | 0.000 |  |  |
| Other | 0.60 | 0.57 | 0.62 | 0.000 |  |  |
| Missing ethnicity | 0.66 | 0.64 | 0.67 | 0.000 |  |  |

| **Outcome: diabetes** | | | | | | |
| --- | --- | --- | --- | --- | --- | --- |
| **Migration status** | **Odds ratio** | **95% LCI** | **95% UCI** | **P value** | **Number of observations** | **Pseudo R squared (Hosmer-Lemeshow)** |
| **Model 1** |  |  |  |  |  |  |
| Probable non-migrant not in vulnerable situations (English as first language without complex social factors) | Reference | Reference | Reference | Reference | 652,880 | 0.016 |
| Probable migrants in vulnerable situations (English not their first language and complex social factors) | 1.46 | 1.31 | 1.61 | 0.000 |  |  |
| Probable migrants not in vulnerable situations (only English not their first language) | 1.43 | 1.33 | 1.53 | 0.000 |  |  |
| Probable non-migrants in vulnerable situations (only complex social factors) | 0.93 | 0.83 | 1.03 | 0.174 |  |  |
| Missing migration status (first language or complex social factor data missing) | 0.41 | 0.39 | 0.44 | 0.000 |  |  |
| **Model 2** | | | | | | |
| Probable non-migrant not in vulnerable situations (English as first language without complex social factors) | Reference | Reference | Reference | Reference | 652,871 | 0.034 |
| Probable migrants in vulnerable situations (English not their first language and complex social factors) | 1.30 | 1.17 | 1.45 | 0.000 |  |  |
| Probable migrants not in vulnerable situations (only English not their first language) | 1.18 | 1.10 | 1.27 | 0.000 |  |  |
| Probable non-migrants in vulnerable situations (only complex social factors) | 1.21 | 1.09 | 1.35 | 0.000 |  |  |
| Missing migration status (first language or complex social factor data missing) | 0.41 | 0.39 | 0.44 | 0.000 |  |  |
| Not most deprived | Reference | Reference | Reference | Reference |  |  |
| Most deprived | 1.68 | 1.57 | 1.78 | 0.000 |  |  |
| Mother's age at booking (years) | 1.06 | 1.06 | 1.07 | 0.000 |  |  |
| White | Reference | Reference | Reference | Reference |  |  |
| Mixed | 1.13 | 0.93 | 1.35 | 0.197 |  |  |
| Asian | 1.88 | 1.75 | 2.01 | 0.000 |  |  |
| Black | 1.40 | 1.26 | 1.56 | 0.000 |  |  |
| Other | 0.90 | 0.78 | 1.04 | 0.172 |  |  |
| Missing ethnicity | 0.94 | 0.87 | 1.02 | 0.125 |  |  |
| **Model 3** | | | | | | |
| Probable non-migrant not in vulnerable situations (English as first language without complex social factors) | Reference | Reference | Reference | Reference | 652,871 | 0.029 |
| Probable migrants in vulnerable situations (English not their first language and complex social factors) | 1.46 | 1.31 | 1.62 | 0.000 |  |  |
| Probable migrants not in vulnerable situations (only English not their first language) | 1.33 | 1.24 | 1.42 | 0.000 |  |  |
| Probable non-migrants in vulnerable situations (only complex social factors) | 1.20 | 1.08 | 1.34 | 0.001 |  |  |
| Missing migration status (first language or complex social factor data missing) | 0.42 | 0.39 | 0.45 | 0.000 |  |  |
| Not most deprived | Reference | Reference | Reference | Reference |  |  |
| Most deprived | 1.75 | 1.65 | 1.87 | 0.000 |  |  |
| Mother's age at booking (years) | 1.07 | 1.06 | 1.07 | 0.000 |  |  |
| **Model 4** | | | | | | |
| Probable non-migrant not in vulnerable situations (English as first language without complex social factors) | Reference | Reference | Reference | Reference | 373,367 | 0.025 |
| Probable migrants in vulnerable situations (English not their first language and complex social factors) | 1.18 | 1.05 | 1.33 | 0.006 |  |  |
| Probable migrants not in vulnerable situations (only English not their first language) | 1.13 | 1.04 | 1.22 | 0.003 |  |  |
| Probable non-migrants in vulnerable situations (only complex social factors) | 1.20 | 1.07 | 1.34 | 0.001 |  |  |
| Not most deprived | Reference | Reference | Reference | Reference |  |  |
| Most deprived | 1.83 | 1.70 | 1.97 | 0.000 |  |  |
| Mother's age at booking (years) | 1.06 | 1.06 | 1.07 | 0.000 |  |  |
| White | Reference | Reference | Reference | Reference |  |  |
| Mixed | 1.15 | 0.93 | 1.39 | 0.185 |  |  |
| Asian | 1.96 | 1.82 | 2.11 | 0.000 |  |  |
| Black | 1.52 | 1.35 | 1.70 | 0.000 |  |  |
| Other | 0.94 | 0.80 | 1.09 | 0.421 |  |  |
| **Model 5** | | | | | | |
| Probable non-migrant not in vulnerable situations (English as first language without complex social factors) | Reference | Reference | Reference | Reference | 622,544 | 0.051 |
| Probable migrants in vulnerable situations (English not their first language and complex social factors) | 1.33 | 1.20 | 1.48 | 0.000 |  |  |
| Probable migrants not in vulnerable situations (only English not their first language) | 1.18 | 1.10 | 1.27 | 0.000 |  |  |
| Probable non-migrants in vulnerable situations (only complex social factors) | 1.21 | 1.06 | 1.37 | 0.003 |  |  |
| Missing migration status (first language or complex social factor data missing) | 0.42 | 0.39 | 0.45 | 0.000 |  |  |
| Not most deprived | Reference | Reference | Reference | Reference |  |  |
| Most deprived | 1.69 | 1.58 | 1.80 | 0.000 |  |  |
| Mother's age at booking (years) | 1.06 | 1.06 | 1.07 | 0.000 |  |  |
| White | Reference | Reference | Reference | Reference |  |  |
| Mixed | 1.15 | 0.95 | 1.38 | 0.149 |  |  |
| Asian | 1.89 | 1.76 | 2.02 | 0.000 |  |  |
| Black | 1.40 | 1.26 | 1.56 | 0.000 |  |  |
| Other | 0.90 | 0.77 | 1.04 | 0.175 |  |  |
| Missing ethnicity | 0.95 | 0.88 | 1.03 | 0.203 |  |  |

| **Outcome: hypertension** | | | | | | |
| --- | --- | --- | --- | --- | --- | --- |
| **Migration status** | **Odds ratio** | **95% LCI** | **95% UCI** | **P value** | **Number of observations** | **Pseudo R squared (Hosmer-Lemeshow)** |
| **Model 1** |  |  |  |  |  |  |
| Probable non-migrant not in vulnerable situations (English as first language without complex social factors) | Reference | Reference | Reference | Reference | 652,880 | 0.02 |
| Probable migrants in vulnerable situations (English not their first language and complex social factors) | 0.60 | 0.52 | 0.68 | 0.000 |  |  |
| Probable migrants not in vulnerable situations (only English not their first language) | 0.89 | 0.82 | 0.95 | 0.001 |  |  |
| Probable non-migrants in vulnerable situations (only complex social factors) | 0.62 | 0.55 | 0.69 | 0.000 |  |  |
| Missing migration status (first language or complex social factor data missing) | 0.30 | 0.28 | 0.32 | 0.000 |  |  |
| **Model 2** | | | | | | |
| Probable non-migrant not in vulnerable situations (English as first language without complex social factors) | Reference | Reference | Reference | Reference | 652,871 | 0.039 |
| Probable migrants in vulnerable situations (English not their first language and complex social factors) | 0.67 | 0.58 | 0.77 | 0.000 |  |  |
| Probable migrants not in vulnerable situations (only English not their first language) | 0.89 | 0.82 | 0.95 | 0.002 |  |  |
| Probable non-migrants in vulnerable situations (only complex social factors) | 0.83 | 0.74 | 0.93 | 0.001 |  |  |
| Missing migration status (first language or complex social factor data missing) | 0.31 | 0.29 | 0.33 | 0.000 |  |  |
| Not most deprived | Reference | Reference | Reference | Reference |  |  |
| Most deprived | 1.44 | 1.34 | 1.54 | 0.000 |  |  |
| Mother's age at booking (years) | 1.07 | 1.07 | 1.08 | 0.000 |  |  |
| White | Reference | Reference | Reference | Reference |  |  |
| Mixed | 1.02 | 0.86 | 1.21 | 0.792 |  |  |
| Asian | 0.85 | 0.78 | 0.92 | 0.000 |  |  |
| Black | 1.68 | 1.53 | 1.84 | 0.000 |  |  |
| Other | 0.72 | 0.62 | 0.84 | 0.000 |  |  |
| Missing ethnicity | 0.57 | 0.52 | 0.62 | 0.000 |  |  |
| **Model 3** | | | | | | |
| Probable non-migrant not in vulnerable situations (English as first language without complex social factors) | Reference | Reference | Reference | Reference | 652,871 | 0.034 |
| Probable migrants in vulnerable situations (English not their first language and complex social factors) | 0.63 | 0.55 | 0.72 | 0.000 |  |  |
| Probable migrants not in vulnerable situations (only English not their first language) | 0.84 | 0.78 | 0.90 | 0.000 |  |  |
| Probable non-migrants in vulnerable situations (only complex social factors) | 0.85 | 0.75 | 0.95 | 0.005 |  |  |
| Missing migration status (first language or complex social factor data missing) | 0.30 | 0.28 | 0.32 | 0.000 |  |  |
| Not most deprived | Reference | Reference | Reference | Reference |  |  |
| Most deprived | 1.47 | 1.38 | 1.57 | 0.000 |  |  |
| Mother's age at booking (years) | 1.07 | 1.07 | 1.08 | 0.000 |  |  |
| **Model 4** | | | | | | |
| Probable non-migrant not in vulnerable situations (English as first language without complex social factors) | Reference | Reference | Reference | Reference | 373,367 | 0.018 |
| Probable migrants in vulnerable situations (English not their first language and complex social factors) | 0.62 | 0.53 | 0.72 | 0.000 |  |  |
| Probable migrants not in vulnerable situations (only English not their first language) | 0.89 | 0.82 | 0.96 | 0.004 |  |  |
| Probable non-migrants in vulnerable situations (only complex social factors) | 0.82 | 0.73 | 0.92 | 0.001 |  |  |
| Not most deprived | Reference | Reference | Reference | Reference |  |  |
| Most deprived | 1.53 | 1.41 | 1.64 | 0.000 |  |  |
| Mother's age at booking (years) | 1.07 | 1.06 | 1.07 | 0.000 |  |  |
| White | Reference | Reference | Reference | Reference |  |  |
| Mixed | 0.91 | 0.74 | 1.10 | 0.341 |  |  |
| Asian | 0.87 | 0.80 | 0.96 | 0.003 |  |  |
| Black | 1.70 | 1.54 | 1.87 | 0.000 |  |  |
| Other | 0.73 | 0.62 | 0.86 | 0.000 |  |  |
| **Model 5** | | | | | | |
| Probable non-migrant not in vulnerable situations (English as first language without complex social factors) | Reference | Reference | Reference | Reference | 622,544 | 0.036 |
| Probable migrants in vulnerable situations (English not their first language and complex social factors) | 0.69 | 0.59 | 0.79 | 0.000 |  |  |
| Probable migrants not in vulnerable situations (only English not their first language) | 0.89 | 0.82 | 0.96 | 0.002 |  |  |
| Probable non-migrants in vulnerable situations (only complex social factors) | 0.88 | 0.77 | 1.00 | 0.051 |  |  |
| Missing migration status (first language or complex social factor data missing) | 0.32 | 0.30 | 0.34 | 0.000 |  |  |
| Not most deprived | Reference | Reference | Reference | Reference |  |  |
| Most deprived | 1.45 | 1.35 | 1.55 | 0.000 |  |  |
| Mother's age at booking (years) | 1.07 | 1.06 | 1.07 | 0.000 |  |  |
| White | Reference | Reference | Reference | Reference |  |  |
| Mixed | 1.02 | 0.85 | 1.21 | 0.847 |  |  |
| Asian | 0.85 | 0.78 | 0.92 | 0.000 |  |  |
| Black | 1.69 | 1.54 | 1.85 | 0.000 |  |  |
| Other | 0.70 | 0.60 | 0.82 | 0.000 |  |  |
| Missing ethnicity | 0.57 | 0.52 | 0.62 | 0.000 |  |  |

| **Outcome: cardiac disease** | | | | | | |
| --- | --- | --- | --- | --- | --- | --- |
| **Migration status** | **Odds ratio** | **95% LCI** | **95% UCI** | **P value** | **Number of observations** | **Pseudo R squared (Hosmer-Lemeshow)** |
| **Model 1** | | | | | | |
| Probable non-migrant not in vulnerable situations (English as first language without complex social factors) | Reference | Reference | Reference | Reference | 652,880 | 0.012 |
| Probable migrants in vulnerable situations (English not their first language and complex social factors) | 0.50 | 0.42 | 0.59 | 0.000 |  |  |
| Probable migrants not in vulnerable situations (only English not their first language) | 0.82 | 0.75 | 0.90 | 0.000 |  |  |
| Probable non-migrants in vulnerable situations (only complex social factors) | 0.84 | 0.75 | 0.94 | 0.003 |  |  |
| Missing migration status (first language or complex social factor data missing) | 0.41 | 0.38 | 0.44 | 0.000 |  |  |
| **Model 2** | | | | | | |
| Probable non-migrant not in vulnerable situations (English as first language without complex social factors) | Reference | Reference | Reference | Reference | 652,871 | 0.019 |
| Probable migrants in vulnerable situations (English not their first language and complex social factors) | 0.61 | 0.51 | 0.72 | 0.000 |  |  |
| Probable migrants not in vulnerable situations (only English not their first language) | 1.00 | 0.91 | 1.09 | 0.994 |  |  |
| Probable non-migrants in vulnerable situations (only complex social factors) | 0.79 | 0.70 | 0.89 | 0.000 |  |  |
| Missing migration status (first language or complex social factor data missing) | 0.42 | 0.39 | 0.45 | 0.000 |  |  |
| Not most deprived | Reference | Reference | Reference | Reference |  |  |
| Most deprived | 1.14 | 1.05 | 1.24 | 0.001 |  |  |
| Mother's age at booking (years) | 1.00 | 0.99 | 1.00 | 0.227 |  |  |
| White | Reference | Reference | Reference | Reference |  |  |
| Mixed | 0.71 | 0.56 | 0.88 | 0.002 |  |  |
| Asian | 0.35 | 0.30 | 0.40 | 0.000 |  |  |
| Black | 0.56 | 0.47 | 0.66 | 0.000 |  |  |
| Other | 0.60 | 0.49 | 0.71 | 0.000 |  |  |
| Missing ethnicity | 0.63 | 0.58 | 0.69 | 0.000 |  |  |
| **Model 3** | | | | | | |
| Probable non-migrant not in vulnerable situations (English as first language without complex social factors) | Reference | Reference | Reference | Reference | 652,871 | 0.012 |
| Probable migrants in vulnerable situations (English not their first language and complex social factors) | 0.48 | 0.40 | 0.57 | 0.000 |  |  |
| Probable migrants not in vulnerable situations (only English not their first language) | 0.82 | 0.75 | 0.89 | 0.000 |  |  |
| Probable non-migrants in vulnerable situations (only complex social factors) | 0.80 | 0.70 | 0.89 | 0.000 |  |  |
| Missing migration status (first language or complex social factor data missing) | 0.40 | 0.37 | 0.43 | 0.000 |  |  |
| Not most deprived | Reference | Reference | Reference | Reference |  |  |
| Most deprived | 1.08 | 1.00 | 1.17 | 0.052 |  |  |
| Mother's age at booking (years) | 0.99 | 0.99 | 1.00 | 0.005 |  |  |
| **Model 4** | | | | | | |
| Probable non-migrant not in vulnerable situations (English as first language without complex social factors) | Reference | Reference | Reference | Reference | 373,367 | 0.009 |
| Probable migrants in vulnerable situations (English not their first language and complex social factors) | 0.59 | 0.48 | 0.71 | 0.000 |  |  |
| Probable migrants not in vulnerable situations (only English not their first language) | 1.00 | 0.90 | 1.10 | 0.956 |  |  |
| Probable non-migrants in vulnerable situations (only complex social factors) | 0.77 | 0.68 | 0.87 | 0.000 |  |  |
| Not most deprived | Reference | Reference | Reference | Reference |  |  |
| Most deprived | 1.29 | 1.17 | 1.41 | 0.000 |  |  |
| Mother's age at booking (years) | 1.00 | 0.99 | 1.00 | 0.198 |  |  |
| White | Reference | Reference | Reference | Reference |  |  |
| Mixed | 0.75 | 0.59 | 0.95 | 0.020 |  |  |
| Asian | 0.37 | 0.32 | 0.43 | 0.000 |  |  |
| Black | 0.63 | 0.52 | 0.75 | 0.000 |  |  |
| Other | 0.64 | 0.52 | 0.77 | 0.000 |  |  |
| **Model 5** | | | | | | |
| Probable non-migrant not in vulnerable situations (English as first language without complex social factors) | Reference | Reference | Reference | Reference | 622,544 | 0.019 |
| Probable migrants in vulnerable situations (English not their first language and complex social factors) | 0.59 | 0.48 | 0.71 | 0.000 |  |  |
| Probable migrants not in vulnerable situations (only English not their first language) | 1.00 | 0.91 | 1.10 | 0.957 |  |  |
| Probable non-migrants in vulnerable situations (only complex social factors) | 0.75 | 0.64 | 0.88 | 0.000 |  |  |
| Missing migration status (first language or complex social factor data missing) | 0.43 | 0.40 | 0.46 | 0.000 |  |  |
| Not most deprived | Reference | Reference | Reference | Reference |  |  |
| Most deprived | 1.16 | 1.06 | 1.25 | 0.001 |  |  |
| Mother's age at booking (years) | 1.00 | 0.99 | 1.00 | 0.093 |  |  |
| White | Reference | Reference | Reference | Reference |  |  |
| Mixed | 0.72 | 0.57 | 0.90 | 0.005 |  |  |
| Asian | 0.34 | 0.30 | 0.39 | 0.000 |  |  |
| Black | 0.53 | 0.44 | 0.63 | 0.000 |  |  |
| Other | 0.61 | 0.50 | 0.73 | 0.000 |  |  |
| Missing ethnicity | 0.63 | 0.57 | 0.69 | 0.000 |  |  |

| **Outcome: thromboembolic condition** | | | | | | |
| --- | --- | --- | --- | --- | --- | --- |
| **Migration status** | **Odds ratio** | **95% LCI** | **95% UCI** | **P value** | **Number of observations** | **Pseudo R squared (Hosmer-Lemeshow)** |
| **Model 1** | | | | | | |
| Probable non-migrant not in vulnerable situations (English as first language without complex social factors) | Reference | Reference | Reference | Reference | 652,880 | 0.017 |
| Probable migrants in vulnerable situations (English not their first language and complex social factors) | 0.59 | 0.49 | 0.70 | 0.000 |  |  |
| Probable migrants not in vulnerable situations (only English not their first language) | 0.95 | 0.87 | 1.05 | 0.333 |  |  |
| Probable non-migrants in vulnerable situations (only complex social factors) | 0.69 | 0.60 | 0.79 | 0.000 |  |  |
| Missing migration status (first language or complex social factor data missing) | 0.31 | 0.29 | 0.34 | 0.000 |  |  |
| **Model 2** | | | | | | |
| Probable non-migrant not in vulnerable situations (English as first language without complex social factors) | Reference | Reference | Reference | Reference | 652,871 | 0.032 |
| Probable migrants in vulnerable situations (English not their first language and complex social factors) | 0.80 | 0.66 | 0.96 | 0.017 |  |  |
| Probable migrants not in vulnerable situations (only English not their first language) | 1.12 | 1.02 | 1.24 | 0.019 |  |  |
| Probable non-migrants in vulnerable situations (only complex social factors) | 0.88 | 0.76 | 1.02 | 0.091 |  |  |
| Missing migration status (first language or complex social factor data missing) | 0.34 | 0.31 | 0.37 | 0.000 |  |  |
| Not most deprived | Reference | Reference | Reference | Reference |  |  |
| Most deprived | 1.36 | 1.24 | 1.49 | 0.000 |  |  |
| Mother's age at booking (years) | 1.06 | 1.05 | 1.07 | 0.000 |  |  |
| White | Reference | Reference | Reference | Reference |  |  |
| Mixed | 0.69 | 0.53 | 0.89 | 0.005 |  |  |
| Asian | 0.37 | 0.32 | 0.43 | 0.000 |  |  |
| Black | 0.55 | 0.45 | 0.65 | 0.000 |  |  |
| Other | 0.71 | 0.58 | 0.85 | 0.000 |  |  |
| Missing ethnicity | 0.49 | 0.44 | 0.55 | 0.000 |  |  |
| **Model 3** | | | | | | |
| Probable non-migrant not in vulnerable situations (English as first language without complex social factors) | Reference | Reference | Reference | Reference | 652,871 | 0.024 |
| Probable migrants in vulnerable situations (English not their first language and complex social factors) | 0.62 | 0.51 | 0.74 | 0.000 |  |  |
| Probable migrants not in vulnerable situations (only English not their first language) | 0.92 | 0.83 | 1.01 | 0.074 |  |  |
| Probable non-migrants in vulnerable situations (only complex social factors) | 0.89 | 0.77 | 1.03 | 0.132 |  |  |
| Missing migration status (first language or complex social factor data missing) | 0.32 | 0.29 | 0.35 | 0.000 |  |  |
| Not most deprived | Reference | Reference | Reference | Reference |  |  |
| Most deprived | 1.28 | 1.17 | 1.40 | 0.000 |  |  |
| Mother's age at booking (years) | 1.06 | 1.05 | 1.06 | 0.000 |  |  |
| **Model 4** | | | | | | |
| Probable non-migrant not in vulnerable situations (English as first language without complex social factors) | Reference | Reference | Reference | Reference | 373,367 | 0.016 |
| Probable migrants in vulnerable situations (English not their first language and complex social factors) | 0.80 | 0.65 | 0.97 | 0.031 |  |  |
| Probable migrants not in vulnerable situations (only English not their first language) | 1.14 | 1.03 | 1.27 | 0.012 |  |  |
| Probable non-migrants in vulnerable situations (only complex social factors) | 0.88 | 0.75 | 1.02 | 0.092 |  |  |
| Not most deprived | Reference | Reference | Reference | Reference |  |  |
| Most deprived | 1.42 | 1.28 | 1.57 | 0.000 |  |  |
| Mother's age at booking (years) | 1.06 | 1.05 | 1.07 | 0.000 |  |  |
| White | Reference | Reference | Reference | Reference |  |  |
| Mixed | 0.70 | 0.52 | 0.91 | 0.010 |  |  |
| Asian | 0.38 | 0.33 | 0.45 | 0.000 |  |  |
| Black | 0.54 | 0.44 | 0.65 | 0.000 |  |  |
| Other | 0.70 | 0.57 | 0.85 | 0.000 |  |  |
| **Model 5** | | | | | | |
| Probable non-migrant not in vulnerable situations (English as first language without complex social factors) | Reference | Reference | Reference | Reference | 622,544 | 0.03 |
| Probable migrants in vulnerable situations (English not their first language and complex social factors) | 0.81 | 0.66 | 0.98 | 0.031 |  |  |
| Probable migrants not in vulnerable situations (only English not their first language) | 1.13 | 1.03 | 1.25 | 0.012 |  |  |
| Probable non-migrants in vulnerable situations (only complex social factors) | 0.91 | 0.77 | 1.08 | 0.304 |  |  |
| Missing migration status (first language or complex social factor data missing) | 0.35 | 0.31 | 0.38 | 0.000 |  |  |
| Not most deprived | Reference | Reference | Reference | Reference |  |  |
| Most deprived | 1.35 | 1.23 | 1.48 | 0.000 |  |  |
| Mother's age at booking (years) | 1.06 | 1.05 | 1.06 | 0.000 |  |  |
| White | Reference | Reference | Reference | Reference |  |  |
| Mixed | 0.69 | 0.53 | 0.89 | 0.006 |  |  |
| Asian | 0.37 | 0.32 | 0.43 | 0.000 |  |  |
| Black | 0.55 | 0.45 | 0.65 | 0.000 |  |  |
| Other | 0.71 | 0.58 | 0.85 | 0.000 |  |  |
| Missing ethnicity | 0.48 | 0.43 | 0.54 | 0.000 |  |  |

| **Outcome: renal disease** | | | | | | |
| --- | --- | --- | --- | --- | --- | --- |
| **Migration status** | **Odds ratio** | **95% LCI** | **95% UCI** | **P value** | **Number of observations** | **Pseudo R squared (Hosmer-Lemeshow)** |
| **Model 1** | | | | | | |
| Probable non-migrant not in vulnerable situations (English as first language without complex social factors) | Reference | Reference | Reference | Reference | 652,880 | 0.004 |
| Probable migrants in vulnerable situations (English not their first language and complex social factors) | 0.79 | 0.68 | 0.92 | 0.002 |  |  |
| Probable migrants not in vulnerable situations (only English not their first language) | 1.02 | 0.93 | 1.11 | 0.677 |  |  |
| Probable non-migrants in vulnerable situations (only complex social factors) | 0.87 | 0.77 | 0.98 | 0.022 |  |  |
| Missing migration status (first language or complex social factor data missing) | 0.61 | 0.57 | 0.65 | 0.000 |  |  |
| **Model 2** |  |  |  |  |  |  |
| Probable non-migrant not in vulnerable situations (English as first language without complex social factors) | Reference | Reference | Reference | Reference | 652,871 | 0.012 |
| Probable migrants in vulnerable situations (English not their first language and complex social factors) | 0.97 | 0.83 | 1.13 | 0.714 |  |  |
| Probable migrants not in vulnerable situations (only English not their first language) | 1.25 | 1.14 | 1.36 | 0.000 |  |  |
| Probable non-migrants in vulnerable situations (only complex social factors) | 0.79 | 0.69 | 0.89 | 0.000 |  |  |
| Missing migration status (first language or complex social factor data missing) | 0.63 | 0.59 | 0.68 | 0.000 |  |  |
| Not most deprived | Reference | Reference | Reference | Reference |  |  |
| Most deprived | 0.83 | 0.76 | 0.91 | 0.000 |  |  |
| Mother's age at booking (years) | 0.98 | 0.98 | 0.99 | 0.000 |  |  |
| White | Reference | Reference | Reference | Reference |  |  |
| Mixed | 0.76 | 0.60 | 0.94 | 0.013 |  |  |
| Asian | 0.42 | 0.37 | 0.48 | 0.000 |  |  |
| Black | 0.37 | 0.30 | 0.46 | 0.000 |  |  |
| Other | 0.74 | 0.63 | 0.87 | 0.000 |  |  |
| Missing ethnicity | 0.58 | 0.53 | 0.64 | 0.000 |  |  |
| **Model 3** | | | | | | |
| Probable non-migrant not in vulnerable situations (English as first language without complex social factors) | Reference | Reference | Reference | Reference | 652,871 | 0.005 |
| Probable migrants in vulnerable situations (English not their first language and complex social factors) | 0.79 | 0.67 | 0.91 | 0.002 |  |  |
| Probable migrants not in vulnerable situations (only English not their first language) | 1.04 | 0.95 | 1.14 | 0.345 |  |  |
| Probable non-migrants in vulnerable situations (only complex social factors) | 0.79 | 0.70 | 0.90 | 0.000 |  |  |
| Missing migration status (first language or complex social factor data missing) | 0.61 | 0.57 | 0.65 | 0.000 |  |  |
| Not most deprived | Reference | Reference | Reference | Reference |  |  |
| Most deprived | 0.79 | 0.72 | 0.86 | 0.000 |  |  |
| Mother's age at booking (years) | 0.98 | 0.98 | 0.99 | 0.000 |  |  |
| **Model 4** |  |  |  |  |  |  |
| Probable non-migrant not in vulnerable situations (English as first language without complex social factors) | Reference | Reference | Reference | Reference | 373,367 | 0.009 |
| Probable migrants in vulnerable situations (English not their first language and complex social factors) | 0.95 | 0.79 | 1.12 | 0.523 |  |  |
| Probable migrants not in vulnerable situations (only English not their first language) | 1.25 | 1.13 | 1.37 | 0.000 |  |  |
| Probable non-migrants in vulnerable situations (only complex social factors) | 0.79 | 0.70 | 0.90 | 0.001 |  |  |
| Not most deprived | Reference | Reference | Reference | Reference |  |  |
| Most deprived | 0.80 | 0.72 | 0.89 | 0.000 |  |  |
| Mother's age at booking (years) | 0.99 | 0.98 | 0.99 | 0.000 |  |  |
| White | Reference | Reference | Reference | Reference |  |  |
| Mixed | 0.70 | 0.53 | 0.89 | 0.006 |  |  |
| Asian | 0.42 | 0.36 | 0.48 | 0.000 |  |  |
| Black | 0.34 | 0.27 | 0.43 | 0.000 |  |  |
| Other | 0.76 | 0.63 | 0.91 | 0.003 |  |  |
| **Model 5** |  |  |  |  |  |  |
| Probable non-migrant not in vulnerable situations (English as first language without complex social factors) | Reference | Reference | Reference | Reference | 622,544 | 0.013 |
| Probable migrants in vulnerable situations (English not their first language and complex social factors) | 1.01 | 0.86 | 1.19 | 0.867 |  |  |
| Probable migrants not in vulnerable situations (only English not their first language) | 1.25 | 1.14 | 1.36 | 0.000 |  |  |
| Probable non-migrants in vulnerable situations (only complex social factors) | 0.83 | 0.70 | 0.97 | 0.026 |  |  |
| Missing migration status (first language or complex social factor data missing) | 0.64 | 0.59 | 0.68 | 0.000 |  |  |
| Not most deprived | Reference | Reference | Reference | Reference |  |  |
| Most deprived | 0.85 | 0.77 | 0.93 | 0.000 |  |  |
| Mother's age at booking (years) | 0.98 | 0.98 | 0.99 | 0.000 |  |  |
| White | Reference | Reference | Reference | Reference |  |  |
| Mixed | 0.76 | 0.60 | 0.94 | 0.015 |  |  |
| Asian | 0.42 | 0.37 | 0.48 | 0.000 |  |  |
| Black | 0.37 | 0.30 | 0.46 | 0.000 |  |  |
| Other | 0.73 | 0.62 | 0.87 | 0.000 |  |  |
| Missing ethnicity | 0.58 | 0.53 | 0.63 | 0.000 |  |  |

| **Outcome: hepatitis b** | | | | | | |
| --- | --- | --- | --- | --- | --- | --- |
| **Migration status** | **Odds ratio** | **95% LCI** | **95% UCI** | **P value** | **Number of observations** | **Pseudo R squared (Hosmer-Lemeshow)** |
| **Model 1** | | | | | | |
| Probable non-migrant not in vulnerable situations (English as first language without complex social factors) | Reference | Reference | Reference | Reference | 652,880 | 0.065 |
| Probable migrants in vulnerable situations (English not their first language and complex social factors) | 7.39 | 6.12 | 8.90 | 0.000 |  |  |
| Probable migrants not in vulnerable situations (only English not their first language) | 5.88 | 5.05 | 6.86 | 0.000 |  |  |
| Probable non-migrants in vulnerable situations (only complex social factors) | 1.00 | 0.69 | 1.40 | 0.997 |  |  |
| Missing migration status (first language or complex social factor data missing) | 0.56 | 0.45 | 0.69 | 0.000 |  |  |
| **Model 2** | | | | | | |
| Probable non-migrant not in vulnerable situations (English as first language without complex social factors) | Reference | Reference | Reference | Reference | 652,871 | 0.099 |
| Probable migrants in vulnerable situations (English not their first language and complex social factors) | 5.40 | 4.43 | 6.58 | 0.000 |  |  |
| Probable migrants not in vulnerable situations (only English not their first language) | 4.44 | 3.78 | 5.21 | 0.000 |  |  |
| Probable non-migrants in vulnerable situations (only complex social factors) | 1.20 | 0.83 | 1.69 | 0.307 |  |  |
| Missing migration status (first language or complex social factor data missing) | 0.54 | 0.44 | 0.67 | 0.000 |  |  |
| Not most deprived | Reference | Reference | Reference | Reference |  |  |
| Most deprived | 1.28 | 1.10 | 1.49 | 0.001 |  |  |
| Mother's age at booking (years) | 1.04 | 1.03 | 1.06 | 0.000 |  |  |
| White | Reference | Reference | Reference | Reference |  |  |
| Mixed | 1.82 | 1.12 | 2.79 | 0.009 |  |  |
| Asian | 2.03 | 1.69 | 2.44 | 0.000 |  |  |
| Black | 6.82 | 5.70 | 8.14 | 0.000 |  |  |
| Other | 2.55 | 1.99 | 3.24 | 0.000 |  |  |
| Missing ethnicity | 1.31 | 1.06 | 1.60 | 0.010 |  |  |
| **Model 3** | | | | | | |
| Probable non-migrant not in vulnerable situations (English as first language without complex social factors) | Reference | Reference | Reference | Reference | 652,871 | 0.073 |
| Probable migrants in vulnerable situations (English not their first language and complex social factors) | 7.55 | 6.23 | 9.13 | 0.000 |  |  |
| Probable migrants not in vulnerable situations (only English not their first language) | 5.56 | 4.77 | 6.49 | 0.000 |  |  |
| Probable non-migrants in vulnerable situations (only complex social factors) | 1.27 | 0.88 | 1.79 | 0.183 |  |  |
| Missing migration status (first language or complex social factor data missing) | 0.56 | 0.45 | 0.69 | 0.000 |  |  |
| Not most deprived | Reference | Reference | Reference | Reference |  |  |
| Most deprived | 1.54 | 1.32 | 1.79 | 0.000 |  |  |
| Mother's age at booking (years) | 1.06 | 1.05 | 1.07 | 0.000 |  |  |
| **Model 4** | | | | | | |
| Probable non-migrant not in vulnerable situations (English as first language without complex social factors) | Reference | Reference | Reference | Reference | 373,367 | 0.1 |
| Probable migrants in vulnerable situations (English not their first language and complex social factors) | 5.85 | 4.72 | 7.24 | 0.000 |  |  |
| Probable migrants not in vulnerable situations (only English not their first language) | 4.87 | 4.10 | 5.80 | 0.000 |  |  |
| Probable non-migrants in vulnerable situations (only complex social factors) | 1.34 | 0.92 | 1.90 | 0.112 |  |  |
| Not most deprived | Reference | Reference | Reference | Reference |  |  |
| Most deprived | 1.26 | 1.05 | 1.49 | 0.010 |  |  |
| Mother's age at booking (years) | 1.04 | 1.03 | 1.06 | 0.000 |  |  |
| White | Reference | Reference | Reference | Reference |  |  |
| Mixed | 1.82 | 1.09 | 2.84 | 0.014 |  |  |
| Asian | 1.96 | 1.61 | 2.38 | 0.000 |  |  |
| Black | 6.46 | 5.33 | 7.80 | 0.000 |  |  |
| Other | 2.40 | 1.85 | 3.09 | 0.000 |  |  |
| **Model 5** | | | | | | |
| Probable non-migrant not in vulnerable situations (English as first language without complex social factors) | Reference | Reference | Reference | Reference | 622,544 | 0.098 |
| Probable migrants in vulnerable situations (English not their first language and complex social factors) | 5.37 | 4.38 | 6.56 | 0.000 |  |  |
| Probable migrants not in vulnerable situations (only English not their first language) | 4.46 | 3.80 | 5.23 | 0.000 |  |  |
| Probable non-migrants in vulnerable situations (only complex social factors) | 1.28 | 0.83 | 1.87 | 0.231 |  |  |
| Missing migration status (first language or complex social factor data missing) | 0.56 | 0.45 | 0.69 | 0.000 |  |  |
| Not most deprived | Reference | Reference | Reference | Reference |  |  |
| Most deprived | 1.28 | 1.09 | 1.49 | 0.002 |  |  |
| Mother's age at booking (years) | 1.04 | 1.03 | 1.05 | 0.000 |  |  |
| White | Reference | Reference | Reference | Reference |  |  |
| Mixed | 1.90 | 1.17 | 2.91 | 0.006 |  |  |
| Asian | 2.03 | 1.68 | 2.44 | 0.000 |  |  |
| Black | 6.86 | 5.72 | 8.20 | 0.000 |  |  |
| Other | 2.47 | 1.91 | 3.16 | 0.000 |  |  |
| Missing ethnicity | 1.32 | 1.07 | 1.62 | 0.008 |  |  |

| **Outcome: cancer** | | | | | | |
| --- | --- | --- | --- | --- | --- | --- |
| **Migration status** | **Odds ratio** | **95% LCI** | **95% UCI** | **P value** | **Number of observations** | **Pseudo R squared (Hosmer-Lemeshow)** |
| **Model 1** | | | | | | |
| Probable non-migrant not in vulnerable situations (English as first language without complex social factors) | Reference | Reference | Reference | Reference | 652,880 | 0.015 |
| Probable migrants in vulnerable situations (English not their first language and complex social factors) | 0.33 | 0.20 | 0.49 | 0.000 |  |  |
| Probable migrants not in vulnerable situations (only English not their first language) | 0.62 | 0.50 | 0.76 | 0.000 |  |  |
| Probable non-migrants in vulnerable situations (only complex social factors) | 0.58 | 0.44 | 0.76 | 0.000 |  |  |
| Missing migration status | 0.31 | 0.26 | 0.37 | 0.000 |  |  |
| **Model 2** | | | | | | |
| Probable non-migrant not in vulnerable situations (English as first language without complex social factors) | Reference | Reference | Reference | Reference | 652,871 | 0.035 |
| Probable migrants in vulnerable situations (English not their first language and complex social factors) | 0.53 | 0.33 | 0.81 | 0.005 |  |  |
| Probable migrants not in vulnerable situations (only English not their first language) | 0.77 | 0.62 | 0.95 | 0.016 |  |  |
| Probable non-migrants in vulnerable situations (only complex social factors) | 0.87 | 0.64 | 1.14 | 0.327 |  |  |
| Missing migration status | 0.35 | 0.29 | 0.41 | 0.000 |  |  |
| Not most deprived | Reference | Reference | Reference | Reference |  |  |
| Most deprived | 0.84 | 0.68 | 1.03 | 0.102 |  |  |
| Mother's age at booking (years) | 1.08 | 1.07 | 1.09 | 0.000 |  |  |
| White | Reference | Reference | Reference | Reference |  |  |
| Mixed | 0.66 | 0.39 | 1.05 | 0.104 |  |  |
| Asian | 0.40 | 0.30 | 0.52 | 0.000 |  |  |
| Black | 0.25 | 0.15 | 0.41 | 0.000 |  |  |
| Other | 0.47 | 0.29 | 0.71 | 0.001 |  |  |
| Missing ethnicity | 0.49 | 0.39 | 0.60 | 0.000 |  |  |
| **Model 3** | | | | | | |
| Probable non-migrant not in vulnerable situations (English as first language without complex social factors) | Reference | Reference | Reference | Reference | 652,781 | 0.026 |
| Probable migrants in vulnerable situations (English not their first language and complex social factors) | 0.39 | 0.24 | 0.59 | 0.000 |  |  |
| Probable migrants not in vulnerable situations (only English not their first language) | 0.62 | 0.50 | 0.76 | 0.000 |  |  |
| Probable non-migrants in vulnerable situations (only complex social factors) | 0.87 | 0.64 | 1.14 | 0.327 |  |  |
| Missing migration status | 0.33 | 0.28 | 0.39 | 0.000 |  |  |
| Not most deprived | Reference | Reference | Reference | Reference |  |  |
| Most deprived | 0.77 | 0.62 | 0.95 | 0.016 |  |  |
| Mother's age at booking (years) | 1.07 | 1.06 | 1.09 | 0.000 |  |  |
| **Model 4** | | | | | | |
| Probable non-migrant not in vulnerable situations (English as first language without complex social factors) | Reference | Reference | Reference | Reference | 373,367 | 0.021 |
| Probable migrants in vulnerable situations (English not their first language and complex social factors) | 0.52 | 0.31 | 0.82 | 0.008 |  |  |
| Probable migrants not in vulnerable situations (only English not their first language) | 0.80 | 0.63 | 0.99 | 0.048 |  |  |
| Probable non-migrants in vulnerable situations (only complex social factors) | 0.89 | 0.66 | 1.18 | 0.440 |  |  |
| Not most deprived | Reference | Reference | Reference | Reference |  |  |
| Most deprived | 0.94 | 0.74 | 1.18 | 0.610 |  |  |
| Mother's age at booking (years) | 1.08 | 1.06 | 1.09 | 0.000 |  |  |
| White | Reference | Reference | Reference | Reference |  |  |
| Mixed | 0.73 | 0.42 | 1.17 | 0.226 |  |  |
| Asian | 0.40 | 0.30 | 0.53 | 0.000 |  |  |
| Black | 0.29 | 0.17 | 0.47 | 0.000 |  |  |
| Other | 0.51 | 0.31 | 0.79 | 0.004 |  |  |
| **Model 5** | | | | | | |
| Probable non-migrant not in vulnerable situations (English as first language without complex social factors) | Reference | Reference | Reference | Reference | 622,544 | 0.033 |
| Probable migrants in vulnerable situations (English not their first language and complex social factors) | 0.48 | 0.28 | 0.75 | 0.003 |  |  |
| Probable migrants not in vulnerable situations (only English not their first language) | 0.77 | 0.62 | 0.95 | 0.017 |  |  |
| Probable non-migrants in vulnerable situations (only complex social factors) | 0.88 | 0.62 | 1.20 | 0.442 |  |  |
| Missing migration status | 0.35 | 0.30 | 0.42 | 0.000 |  |  |
| Not most deprived | Reference | Reference | Reference | Reference |  |  |
| Most deprived | 0.84 | 0.67 | 1.04 | 0.114 |  |  |
| Mother's age at booking (years) | 1.08 | 1.07 | 1.09 | 0.000 |  |  |
| White | Reference | Reference | Reference | Reference |  |  |
| Mixed | 0.60 | 0.33 | 0.97 | 0.055 |  |  |
| Asian | 0.40 | 0.30 | 0.52 | 0.000 |  |  |
| Black | 0.26 | 0.15 | 0.41 | 0.000 |  |  |
| Other | 0.48 | 0.29 | 0.72 | 0.001 |  |  |
| Missing ethnicity | 0.48 | 0.39 | 0.60 | 0.000 |  |  |

| **Outcome: family history of inherited disease** | | | | | | |
| --- | --- | --- | --- | --- | --- | --- |
| **Migration status** | **Odds ratio** | **95% LCI** | **95% UCI** | **P value** | **Number of observations** | **Pseudo R squared (Hosmer-Lemeshow)** |
| **Model 1** | | | | | | |
| Probable non-migrant not in vulnerable situations (English as first language without complex social factors) | Reference | Reference | Reference | Reference | 652,880 | 0.008 |
| Probable migrants in vulnerable situations (English not their first language and complex social factors) | 0.36 | 0.31 | 0.41 | 0.000 |  |  |
| Probable migrants not in vulnerable situations (only English not their first language) | 0.71 | 0.67 | 0.75 | 0.000 |  |  |
| Probable non-migrants in vulnerable situations (only complex social factors) | 1.15 | 1.07 | 1.22 | 0.000 |  |  |
| Missing migration status (first language or complex social factor data missing) | 0.59 | 0.56 | 0.61 | 0.000 |  |  |
| **Model 2** | | | | | | |
| Probable non-migrant not in vulnerable situations (English as first language without complex social factors) | Reference | Reference | Reference | Reference | 652,871 | 0.015 |
| Probable migrants in vulnerable situations (English not their first language and complex social factors) | 0.44 | 0.39 | 0.51 | 0.000 |  |  |
| Probable migrants not in vulnerable situations (only English not their first language) | 0.85 | 0.80 | 0.91 | 0.000 |  |  |
| Probable non-migrants in vulnerable situations (only complex social factors) | 1.08 | 1.00 | 1.15 | 0.039 |  |  |
| Missing migration status (first language or complex social factor data missing) | 0.61 | 0.58 | 0.64 | 0.000 |  |  |
| Not most deprived | Reference | Reference | Reference | Reference |  |  |
| Most deprived | 0.98 | 0.93 | 1.03 | 0.498 |  |  |
| Mother's age at booking (years) | 0.99 | 0.99 | 1.00 | 0.000 |  |  |
| White | Reference | Reference | Reference | Reference |  |  |
| Mixed | 0.83 | 0.73 | 0.95 | 0.006 |  |  |
| Asian | 0.53 | 0.49 | 0.57 | 0.000 |  |  |
| Black | 0.43 | 0.38 | 0.49 | 0.000 |  |  |
| Other | 0.41 | 0.36 | 0.48 | 0.000 |  |  |
| Missing ethnicity | 0.60 | 0.57 | 0.64 | 0.000 |  |  |
| **Model 3** | | | | | | |
| Probable non-migrant not in vulnerable situations (English as first language without complex social factors) | Reference | Reference | Reference | Reference | 652,871 | 0.008 |
| Probable migrants in vulnerable situations (English not their first language and complex social factors) | 0.35 | 0.31 | 0.40 | 0.000 |  |  |
| Probable migrants not in vulnerable situations (only English not their first language) | 0.72 | 0.67 | 0.76 | 0.000 |  |  |
| Probable non-migrants in vulnerable situations (only complex social factors) | 1.08 | 1.01 | 1.15 | 0.033 |  |  |
| Missing migration status (first language or complex social factor data missing) | 0.58 | 0.56 | 0.61 | 0.000 |  |  |
| Not most deprived | Reference | Reference | Reference | Reference |  |  |
| Most deprived | 0.94 | 0.89 | 0.98 | 0.011 |  |  |
| Mother's age at booking (years) | 0.99 | 0.99 | 0.99 | 0.000 |  |  |
| **Model 4** | | | | | | |
| Probable non-migrant not in vulnerable situations (English as first language without complex social factors) | Reference | Reference | Reference | Reference | 373,367 | 0.009 |
| Probable migrants in vulnerable situations (English not their first language and complex social factors) | 0.46 | 0.40 | 0.53 | 0.000 |  |  |
| Probable migrants not in vulnerable situations (only English not their first language) | 0.86 | 0.81 | 0.93 | 0.000 |  |  |
| Probable non-migrants in vulnerable situations (only complex social factors) | 1.08 | 1.01 | 1.17 | 0.030 |  |  |
| Not most deprived | Reference | Reference | Reference | Reference |  |  |
| Most deprived | 1.09 | 1.02 | 1.16 | 0.007 |  |  |
| Mother's age at booking (years) | 0.99 | 0.99 | 1.00 | 0.000 |  |  |
| White | Reference | Reference | Reference | Reference |  |  |
| Mixed | 0.84 | 0.72 | 0.97 | 0.020 |  |  |
| Asian | 0.61 | 0.56 | 0.66 | 0.000 |  |  |
| Black | 0.51 | 0.45 | 0.58 | 0.000 |  |  |
| Other | 0.45 | 0.38 | 0.52 | 0.000 |  |  |
| **Model 5** | | | | | | |
| Probable non-migrant not in vulnerable situations (English as first language without complex social factors) | Reference | Reference | Reference | Reference | 622,544 | 0.014 |
| Probable migrants in vulnerable situations (English not their first language and complex social factors) | 0.42 | 0.36 | 0.49 | 0.000 |  |  |
| Probable migrants not in vulnerable situations (only English not their first language) | 0.85 | 0.80 | 0.91 | 0.000 |  |  |
| Probable non-migrants in vulnerable situations (only complex social factors) | 1.04 | 0.95 | 1.14 | 0.370 |  |  |
| Missing migration status (first language or complex social factor data missing) | 0.63 | 0.60 | 0.66 | 0.000 |  |  |
| Not most deprived | Reference | Reference | Reference | Reference |  |  |
| Most deprived | 0.99 | 0.94 | 1.04 | 0.658 |  |  |
| Mother's age at booking (years) | 0.99 | 0.99 | 1.00 | 0.000 |  |  |
| White | Reference | Reference | Reference | Reference |  |  |
| Mixed | 0.84 | 0.73 | 0.95 | 0.009 |  |  |
| Asian | 0.53 | 0.49 | 0.57 | 0.000 |  |  |
| Black | 0.43 | 0.38 | 0.49 | 0.000 |  |  |
| Other | 0.42 | 0.36 | 0.48 | 0.000 |  |  |
| Missing ethnicity | 0.61 | 0.57 | 0.64 | 0.000 |  |  |

| **Outcome: family history of diabetes** | | | | | | |
| --- | --- | --- | --- | --- | --- | --- |
| **Migration status** | **Odds ratio** | **95% LCI** | **95% UCI** | **P value** | **Number of observations** | **Pseudo R squared (Hosmer-Lemeshow)** |
| **Model 1** | | | | | | |
| Probable non-migrant not in vulnerable situations (English as first language without complex social factors) | Reference | Reference | Reference | Reference | 652,880 | 0.052 |
| Probable migrants in vulnerable situations (English not their first language and complex social factors) | 1.13 | 1.10 | 1.17 | 0.000 |  |  |
| Probable migrants not in vulnerable situations (only English not their first language) | 1.27 | 1.24 | 1.29 | 0.000 |  |  |
| Probable non-migrants in vulnerable situations (only complex social factors) | 0.97 | 0.94 | 0.99 | 0.007 |  |  |
| Missing migration status | 0.26 | 0.26 | 0.27 | 0.000 |  |  |
| **Model 2** | | | | | | |
| Probable non-migrant not in vulnerable situations (English as first language without complex social factors) | Reference | Reference | Reference | Reference | 652,871 | 0.072 |
| Probable migrants in vulnerable situations (English not their first language and complex social factors) | 0.89 | 0.86 | 0.92 | 0.000 |  |  |
| Probable migrants not in vulnerable situations (only English not their first language) | 1.01 | 0.99 | 1.03 | 0.401 |  |  |
| Probable non-migrants in vulnerable situations (only complex social factors) | 1.00 | 0.97 | 1.02 | 0.888 |  |  |
| Missing migration status | 0.25 | 0.25 | 0.26 | 0.000 |  |  |
| Not most deprived | Reference | Reference | Reference | Reference |  |  |
| Most deprived | 1.28 | 1.26 | 1.31 | 0.000 |  |  |
| Mother's age at booking (years) | 1.00 | 1.00 | 1.01 | 0.000 |  |  |
| White | Reference | Reference | Reference | Reference |  |  |
| Mixed | 1.55 | 1.48 | 1.62 | 0.000 |  |  |
| Asian | 2.85 | 2.80 | 2.90 | 0.000 |  |  |
| Black | 1.41 | 1.37 | 1.45 | 0.000 |  |  |
| Other | 1.31 | 1.26 | 1.35 | 0.000 |  |  |
| Missing ethnicity | 0.99 | 0.97 | 1.00 | 0.123 |  |  |
| **Model 3** | | | | | | |
| Probable non-migrant not in vulnerable situations (English as first language without complex social factors) | Reference | Reference | Reference | Reference | 652,871 | 0.054 |
| Probable migrants in vulnerable situations (English not their first language and complex social factors) | 1.10 | 1.06 | 1.13 | 0.000 |  |  |
| Probable migrants not in vulnerable situations (only English not their first language) | 1.23 | 1.21 | 1.26 | 0.000 |  |  |
| Probable non-migrants in vulnerable situations (only complex social factors) | 0.99 | 0.96 | 1.01 | 0.301 |  |  |
| Missing migration status | 0.26 | 0.26 | 0.27 | 0.000 |  |  |
| Not most deprived | Reference | Reference | Reference | Reference |  |  |
| Most deprived | 1.35 | 1.33 | 1.38 | 0.000 |  |  |
| Mother's age at booking (years) | 1.01 | 1.01 | 1.01 | 0.000 |  |  |
| **Model 4** | | | | | | |
| Probable non-migrant not in vulnerable situations (English as first language without complex social factors) | Reference | Reference | Reference | Reference | 373,367 | 0.032 |
| Probable migrants in vulnerable situations (English not their first language and complex social factors) | 0.83 | 0.80 | 0.86 | 0.000 |  |  |
| Probable migrants not in vulnerable situations (only English not their first language) | 0.98 | 0.96 | 1.00 | 0.046 |  |  |
| Probable non-migrants in vulnerable situations (only complex social factors) | 1.02 | 0.99 | 1.05 | 0.129 |  |  |
| Not most deprived | Reference | Reference | Reference | Reference |  |  |
| Most deprived | 1.37 | 1.34 | 1.40 | 0.000 |  |  |
| Mother's age at booking (years) | 1.00 | 1.00 | 1.01 | 0.000 |  |  |
| White | Reference | Reference | Reference | Reference |  |  |
| Mixed | 1.58 | 1.51 | 1.66 | 0.000 |  |  |
| Asian | 3.21 | 3.15 | 3.28 | 0.000 |  |  |
| Black | 1.53 | 1.49 | 1.59 | 0.000 |  |  |
| Other | 1.38 | 1.33 | 1.43 | 0.000 |  |  |
| **Model 5** | | | | | | |
| Probable non-migrant not in vulnerable situations (English as first language without complex social factors) | Reference | Reference | Reference | Reference | 622,544 | 0.073 |
| Probable migrants in vulnerable situations (English not their first language and complex social factors) | 0.92 | 0.89 | 0.95 | 0.000 |  |  |
| Probable migrants not in vulnerable situations (only English not their first language) | 1.01 | 0.99 | 1.03 | 0.458 |  |  |
| Probable non-migrants in vulnerable situations (only complex social factors) | 1.09 | 1.05 | 1.13 | 0.000 |  |  |
| Missing migration status | 0.26 | 0.25 | 0.26 | 0.000 |  |  |
| Not most deprived | Reference | Reference | Reference | Reference |  |  |
| Most deprived | 1.30 | 1.28 | 1.32 | 0.000 |  |  |
| Mother's age at booking (years) | 1.00 | 1.00 | 1.00 | 0.001 |  |  |
| White | Reference | Reference | Reference | Reference |  |  |
| Mixed | 1.56 | 1.49 | 1.63 | 0.000 |  |  |
| Asian | 2.86 | 2.80 | 2.91 | 0.000 |  |  |
| Black | 1.41 | 1.37 | 1.45 | 0.000 |  |  |
| Other | 1.32 | 1.27 | 1.37 | 0.000 |  |  |
| Missing ethnicity | 0.99 | 0.97 | 1.01 | 0.325 |  |  |

| **Outcome: booking after 10 weeks gestations** | | | | | | |
| --- | --- | --- | --- | --- | --- | --- |
| **Migration status** | **Odds ratio** | **95% LCI** | **95% UCI** | **P value** | **Number of observations** | **Pseudo R squared (Hosmer-Lemeshow)** |
| **Model 1** | | | | | | |
| Probable non-migrant not in vulnerable situations (English as first language without complex social factors) | Reference | Reference | Reference | Reference | 652,309 | 0.008 |
| Probable migrants in vulnerable situations (English not their first language and complex social factors) | 2.66 | 2.59 | 2.73 | 0.000 |  |  |
| Probable migrants not in vulnerable situations (only English not their first language) | 1.35 | 1.33 | 1.38 | 0.000 |  |  |
| Probable non-migrants in vulnerable situations (only complex social factors) | 1.44 | 1.41 | 1.47 | 0.000 |  |  |
| Missing migration status (first language or complex social factor data missing) | 1.19 | 1.18 | 1.21 | 0.000 |  |  |
| **Model 2^** | | | | | | |
| Probable non-migrant not in vulnerable situations (English as first language without complex social factors) | Reference | Reference | Reference | Reference | 500,439 | 0.018 |
| Probable migrants in vulnerable situations (English not their first language and complex social factors) | 2.25 | 2.18 | 2.32 | 0.000 |  |  |
| Probable migrants not in vulnerable situations (only English not their first language) | 1.22 | 1.20 | 1.24 | 0.000 |  |  |
| Probable non-migrants in vulnerable situations (only complex social factors) | 1.38 | 1.34 | 1.42 | 0.000 |  |  |
| Missing migration status (first language or complex social factor data missing) | 1.15 | 1.14 | 1.17 | 0.000 |  |  |
| Not most deprived | Reference | Reference | Reference | Reference |  |  |
| Most deprived | 1.07 | 1.06 | 1.09 | 0.000 |  |  |
| Mother's age at booking (years) | 1.00 | 1.00 | 1.00 | 0.000 |  |  |
| Number of previous live births | 1.14 | 1.13 | 1.14 | 0.000 |  |  |
| White | Reference | Reference | Reference | Reference |  |  |
| Mixed | 1.35 | 1.29 | 1.41 | 0.000 |  |  |
| Asian | 1.29 | 1.27 | 1.32 | 0.000 |  |  |
| Black | 1.99 | 1.94 | 2.05 | 0.000 |  |  |
| Other | 1.69 | 1.63 | 1.74 | 0.000 |  |  |
| Missing ethnicity | 1.18 | 1.16 | 1.20 | 0.000 |  |  |
| **Model 3^** | | | | | | |
| Probable non-migrant not in vulnerable situations (English as first language without complex social factors) | Reference | Reference | Reference | Reference | 500,439 | 0.013 |
| Probable migrants in vulnerable situations (English not their first language and complex social factors) | 2.54 | 2.47 | 2.62 | 0.000 |  |  |
| Probable migrants not in vulnerable situations (only English not their first language) | 1.33 | 1.31 | 1.35 | 0.000 |  |  |
| Probable non-migrants in vulnerable situations (only complex social factors) | 1.39 | 1.35 | 1.44 | 0.000 |  |  |
| Missing migration status (first language or complex social factor data missing) | 1.16 | 1.15 | 1.18 | 0.000 |  |  |
| Not most deprived | Reference | Reference | Reference | Reference |  |  |
| Most deprived | 1.12 | 1.10 | 1.14 | 0.000 |  |  |
| Mother's age at booking (years) | 1.01 | 1.00 | 1.01 | 0.000 |  |  |
| Number of previous live births | 1.14 | 1.13 | 1.14 | 0.000 |  |  |
| **Model 4^** | | | | | | |
| Probable non-migrant not in vulnerable situations (English as first language without complex social factors) | Reference | Reference | Reference | Reference | 322,673 | 0.02 |
| Probable migrants in vulnerable situations (English not their first language and complex social factors) | 2.27 | 2.20 | 2.35 | 0.000 |  |  |
| Probable migrants not in vulnerable situations (only English not their first language) | 1.21 | 1.18 | 1.23 | 0.000 |  |  |
| Probable non-migrants in vulnerable situations (only complex social factors) | 1.37 | 1.33 | 1.42 | 0.000 |  |  |
| Not most deprived | Reference | Reference | Reference | Reference |  |  |
| Most deprived | 1.00 | 0.98 | 1.02 | 0.801 |  |  |
| Mother's age at booking (years) | 1.00 | 1.00 | 1.01 | 0.000 |  |  |
| Number of previous live births | 1.13 | 1.12 | 1.13 | 0.000 |  |  |
| White | Reference | Reference | Reference | Reference |  |  |
| Mixed | 1.28 | 1.22 | 1.35 | 0.000 |  |  |
| Asian | 1.27 | 1.24 | 1.30 | 0.000 |  |  |
| Black | 1.92 | 1.86 | 1.98 | 0.000 |  |  |
| Other | 1.68 | 1.62 | 1.74 | 0.000 |  |  |
| **Model 5^** |  |  |  |  |  |  |
| Probable non-migrant not in vulnerable situations (English as first language without complex social factors) | Reference | Reference | Reference | Reference | 500,439 | 0.018 |
| Probable migrants in vulnerable situations (English not their first language and complex social factors) | 2.25 | 2.18 | 2.32 | 0.000 |  |  |
| Probable migrants not in vulnerable situations (only English not their first language) | 1.22 | 1.20 | 1.24 | 0.000 |  |  |
| Probable non-migrants in vulnerable situations (only complex social factors) | 1.38 | 1.34 | 1.42 | 0.000 |  |  |
| Missing migration status (first language or complex social factor data missing) | 1.15 | 1.14 | 1.17 | 0.000 |  |  |
| Not most deprived | Reference | Reference | Reference | Reference |  |  |
| Most deprived | 1.07 | 1.06 | 1.09 | 0.000 |  |  |
| Mother's age at booking (years) | 1.00 | 1.00 | 1.00 | 0.000 |  |  |
| Number of previous live births | 1.14 | 1.13 | 1.14 | 0.000 |  |  |
| White | Reference | Reference | Reference | Reference |  |  |
| Mixed | 1.35 | 1.29 | 1.41 | 0.000 |  |  |
| Asian | 1.29 | 1.27 | 1.32 | 0.000 |  |  |
| Black | 1.99 | 1.94 | 2.05 | 0.000 |  |  |
| Other | 1.69 | 1.63 | 1.74 | 0.000 |  |  |
| Missing ethnicity | 1.18 | 1.16 | 1.20 | 0.000 |  |  |

| **Outcome: booking after 16 weeks gestation** | | | | | | |
| --- | --- | --- | --- | --- | --- | --- |
| **Migration status** | **Odds ratio** | **95% LCI** | **95% UCI** | **P value** | **Number of observations** | **Pseudo R squared (Hosmer-Lemeshow)** |
| **Model 1** | | | | | | |
| Probable non-migrant not in vulnerable situations (English as first language without complex social factors) | Reference | Reference | Reference | Reference | 652,309 | 0.026 |
| Probable migrants in vulnerable situations (English not their first language and complex social factors) | 5.04 | 4.88 | 5.22 | 0.000 |  |  |
| Probable migrants not in vulnerable situations (only English not their first language) | 1.62 | 1.57 | 1.67 | 0.000 |  |  |
| Probable non-migrants in vulnerable situations (only complex social factors) | 2.43 | 2.35 | 2.52 | 0.000 |  |  |
| Missing migration status (first language or complex social factor data missing) | 1.92 | 1.88 | 1.96 | 0.000 |  |  |
| **Model 2^** | | | | | | |
| Probable non-migrant not in vulnerable situations (English as first language without complex social factors) | Reference | Reference | Reference | Reference | 500,439 | 0.042 |
| Probable migrants in vulnerable situations (English not their first language and complex social factors) | 3.69 | 3.54 | 3.83 | 0.000 |  |  |
| Probable migrants not in vulnerable situations (only English not their first language) | 1.43 | 1.38 | 1.48 | 0.000 |  |  |
| Probable non-migrants in vulnerable situations (only complex social factors) | 2.23 | 2.12 | 2.34 | 0.000 |  |  |
| Missing migration status (first language or complex social factor data missing) | 1.65 | 1.61 | 1.70 | 0.000 |  |  |
| Not most deprived | Reference | Reference | Reference | Reference |  |  |
| Most deprived | 1.09 | 1.06 | 1.13 | 0.000 |  |  |
| Mother's age at booking (years) | 0.97 | 0.97 | 0.97 | 0.000 |  |  |
| Number of previous live births | 1.19 | 1.18 | 1.20 | 0.000 |  |  |
| White | Reference | Reference | Reference | Reference |  |  |
| Mixed | 1.54 | 1.43 | 1.66 | 0.000 |  |  |
| Asian | 1.19 | 1.15 | 1.23 | 0.000 |  |  |
| Black | 2.28 | 2.18 | 2.37 | 0.000 |  |  |
| Other | 2.05 | 1.95 | 2.15 | 0.000 |  |  |
| Missing ethnicity | 1.71 | 1.66 | 1.76 | 0.000 |  |  |
| **Model 3^** | | | | | | |
| Probable non-migrant not in vulnerable situations (English as first language without complex social factors) | Reference | Reference | Reference | Reference | 500,439 | 0.032 |
| Probable migrants in vulnerable situations (English not their first language and complex social factors) | 4.39 | 4.22 | 4.55 | 0.000 |  |  |
| Probable migrants not in vulnerable situations (only English not their first language) | 1.61 | 1.55 | 1.66 | 0.000 |  |  |
| Probable non-migrants in vulnerable situations (only complex social factors) | 2.25 | 2.14 | 2.36 | 0.000 |  |  |
| Missing migration status (first language or complex social factor data missing) | 1.70 | 1.66 | 1.74 | 0.000 |  |  |
| Not most deprived | Reference | Reference | Reference | Reference |  |  |
| Most deprived | 1.16 | 1.13 | 1.19 | 0.000 |  |  |
| Mother's age at booking (years) | 0.98 | 0.97 | 0.98 | 0.000 |  |  |
| Number of previous live births | 1.18 | 1.17 | 1.19 | 0.000 |  |  |
| **Model 4^** | | | | | | |
| Probable non-migrant not in vulnerable situations (English as first language without complex social factors) | Reference | Reference | Reference | Reference | 322,673 | 0.049 |
| Probable migrants in vulnerable situations (English not their first language and complex social factors) | 3.85 | 3.68 | 4.03 | 0.000 |  |  |
| Probable migrants not in vulnerable situations (only English not their first language) | 1.47 | 1.42 | 1.53 | 0.000 |  |  |
| Probable non-migrants in vulnerable situations (only complex social factors) | 2.21 | 2.09 | 2.32 | 0.000 |  |  |
| Not most deprived | Reference | Reference | Reference | Reference |  |  |
| Most deprived | 1.02 | 0.98 | 1.06 | 0.433 |  |  |
| Mother's age at booking (years) | 0.97 | 0.97 | 0.97 | 0.000 |  |  |
| Number of previous live births | 1.19 | 1.17 | 1.20 | 0.000 |  |  |
| White | Reference | Reference | Reference | Reference |  |  |
| Mixed | 1.55 | 1.42 | 1.69 | 0.000 |  |  |
| Asian | 1.14 | 1.10 | 1.19 | 0.000 |  |  |
| Black | 2.31 | 2.20 | 2.43 | 0.000 |  |  |
| Other | 1.94 | 1.84 | 2.06 | 0.000 |  |  |
| **Model 5^** | | | | | | |
| Probable non-migrant not in vulnerable situations (English as first language without complex social factors) | Reference | Reference | Reference | Reference | 500,439 | 0.042 |
| Probable migrants in vulnerable situations (English not their first language and complex social factors) | 3.69 | 3.54 | 3.83 | 0.000 |  |  |
| Probable migrants not in vulnerable situations (only English not their first language) | 1.43 | 1.38 | 1.48 | 0.000 |  |  |
| Probable non-migrants in vulnerable situations (only complex social factors) | 2.23 | 2.12 | 2.34 | 0.000 |  |  |
| Missing migration status (first language or complex social factor data missing) | 1.65 | 1.61 | 1.70 | 0.000 |  |  |
| Not most deprived | Reference | Reference | Reference | Reference |  |  |
| Most deprived | 1.09 | 1.06 | 1.13 | 0.000 |  |  |
| Mother's age at booking (years) | 0.97 | 0.97 | 0.97 | 0.000 |  |  |
| Number of previous live births | 1.19 | 1.18 | 1.20 | 0.000 |  |  |
| White | Reference | Reference | Reference | Reference |  |  |
| Mixed | 1.54 | 1.43 | 1.66 | 0.000 |  |  |
| Asian | 1.19 | 1.15 | 1.23 | 0.000 |  |  |
| Black | 2.28 | 2.18 | 2.37 | 0.000 |  |  |
| Other | 2.05 | 1.95 | 2.15 | 0.000 |  |  |
| Missing ethnicity | 1.71 | 1.66 | 1.76 | 0.000 |  |  |

| **Outcome: booking after 20 weeks gestation** | | | | | | |
| --- | --- | --- | --- | --- | --- | --- |
| **Migration status** | **Odds ratio** | **95% LCI** | **95% UCI** | **P value** | **Number of observations** | **Pseudo R squared (Hosmer-Lemeshow)** |
| **Model 1** | | | | | | |
| Probable non-migrant not in vulnerable situations (English as first language without complex social factors) | Reference | Reference | Reference | Reference | 652,309 | 0.025 |
| Probable migrants in vulnerable situations (English not their first language and complex social factors) | 4.69 | 4.50 | 4.88 | 0.000 |  |  |
| Probable migrants not in vulnerable situations (only English not their first language) | 1.45 | 1.39 | 1.51 | 0.000 |  |  |
| Probable non-migrants in vulnerable situations (only complex social factors) | 2.39 | 2.29 | 2.49 | 0.000 |  |  |
| Missing migration status (first language or complex social factor data missing) | 2.17 | 2.11 | 2.22 | 0.000 |  |  |
| **Model 2^** | | | | | | |
| Probable non-migrant not in vulnerable situations (English as first language without complex social factors) | Reference | Reference | Reference | Reference | 524,215 | 0.038 |
| Probable migrants in vulnerable situations (English not their first language and complex social factors) | 3.69 | 3.53 | 3.86 | 0.000 |  |  |
| Probable migrants not in vulnerable situations (only English not their first language) | 1.30 | 1.24 | 1.35 | 0.000 |  |  |
| Probable non-migrants in vulnerable situations (only complex social factors) | 2.13 | 2.03 | 2.23 | 0.000 |  |  |
| Missing migration status (first language or complex social factor data missing) | 1.84 | 1.78 | 1.89 | 0.000 |  |  |
| Not most deprived | Reference | Reference | Reference | Reference |  |  |
| Most deprived | 1.03 | 1.00 | 1.07 | 0.057 |  |  |
| Mother's age at booking (years) | 0.98 | 0.97 | 0.98 | 0.000 |  |  |
| Number of previous live births | 1.11 | 1.10 | 1.12 | 0.000 |  |  |
| White | Reference | Reference | Reference | Reference |  |  |
| Mixed | 1.45 | 1.33 | 1.58 | 0.000 |  |  |
| Asian | 1.13 | 1.08 | 1.18 | 0.000 |  |  |
| Black | 2.10 | 2.00 | 2.20 | 0.000 |  |  |
| Other | 2.04 | 1.93 | 2.15 | 0.000 |  |  |
| Missing ethnicity | 1.81 | 1.75 | 1.87 | 0.000 |  |  |
| **Model 3^** | | | | | | |
| Probable non-migrant not in vulnerable situations (English as first language without complex social factors) | Reference | Reference | Reference | Reference | 524,215 | 0.028 |
| Probable migrants in vulnerable situations (English not their first language and complex social factors) | 4.36 | 4.18 | 4.55 | 0.000 |  |  |
| Probable migrants not in vulnerable situations (only English not their first language) | 1.44 | 1.39 | 1.51 | 0.000 |  |  |
| Probable non-migrants in vulnerable situations (only complex social factors) | 2.14 | 2.04 | 2.24 | 0.000 |  |  |
| Missing migration status (first language or complex social factor data missing) | 1.89 | 1.83 | 1.95 | 0.000 |  |  |
| Not most deprived | Reference | Reference | Reference | Reference |  |  |
| Most deprived | 1.09 | 1.05 | 1.13 | 0.000 |  |  |
| Mother's age at booking (years) | 0.98 | 0.98 | 0.98 | 0.000 |  |  |
| Number of previous live births | 1.10 | 1.09 | 1.11 | 0.000 |  |  |
| **Model 4^** | | | | | | |
| Probable non-migrant not in vulnerable situations (English as first language without complex social factors) | Reference | Reference | Reference | Reference | 337,622 | 0.042 |
| Probable migrants in vulnerable situations (English not their first language and complex social factors) | 3.92 | 3.73 | 4.13 | 0.000 |  |  |
| Probable migrants not in vulnerable situations (only English not their first language) | 1.33 | 1.26 | 1.39 | 0.000 |  |  |
| Probable non-migrants in vulnerable situations (only complex social factors) | 2.15 | 2.04 | 2.27 | 0.000 |  |  |
| Not most deprived | Reference | Reference | Reference | Reference |  |  |
| Most deprived | 0.96 | 0.92 | 1.00 | 0.076 |  |  |
| Mother's age at booking (years) | 0.98 | 0.97 | 0.98 | 0.000 |  |  |
| Number of previous live births | 1.11 | 1.09 | 1.12 | 0.000 |  |  |
| White | Reference | Reference | Reference | Reference |  |  |
| Mixed | 1.51 | 1.37 | 1.67 | 0.000 |  |  |
| Asian | 1.08 | 1.03 | 1.14 | 0.002 |  |  |
| Black | 2.14 | 2.02 | 2.26 | 0.000 |  |  |
| Other | 1.96 | 1.84 | 2.09 | 0.000 |  |  |
| **Model 5^** | | | | | | |
| Probable non-migrant not in vulnerable situations (English as first language without complex social factors) | Reference | Reference | Reference | Reference | 500,439 | 0.035 |
| Probable migrants in vulnerable situations (English not their first language and complex social factors) | 3.52 | 3.36 | 3.69 | 0.000 |  |  |
| Probable migrants not in vulnerable situations (only English not their first language) | 1.29 | 1.24 | 1.35 | 0.000 |  |  |
| Probable non-migrants in vulnerable situations (only complex social factors) | 2.36 | 2.23 | 2.50 | 0.000 |  |  |
| Missing migration status (first language or complex social factor data missing) | 1.82 | 1.77 | 1.88 | 0.000 |  |  |
| Not most deprived | Reference | Reference | Reference | Reference |  |  |
| Most deprived | 1.03 | 0.99 | 1.07 | 0.101 |  |  |
| Mother's age at booking (years) | 0.98 | 0.97 | 0.98 | 0.000 |  |  |
| Number of previous live births | 1.10 | 1.09 | 1.11 | 0.000 |  |  |
| White | Reference | Reference | Reference | Reference |  |  |
| Mixed | 1.51 | 1.38 | 1.65 | 0.000 |  |  |
| Asian | 1.16 | 1.11 | 1.21 | 0.000 |  |  |
| Black | 2.13 | 2.02 | 2.24 | 0.000 |  |  |
| Other | 2.06 | 1.94 | 2.18 | 0.000 |  |  |
| Missing ethnicity | 1.84 | 1.77 | 1.90 | 0.000 |  |  |
